# The protective value of miR-204-5p for prognosis and its potential gene network in various malignancies: a comprehensive exploration based on RNA-seq high-throughput data and bioinformatics

**DOI:** 10.18632/oncotarget.21950

**Published:** 2017-10-23

**Authors:** Zhi-Hua Ye, Dong-Yue Wen, Xiao-Yong Cai, Liang Liang, Pei-Rong Wu, Hui Qin, Hong Yang, Yun He, Gang Chen

**Affiliations:** ^1^ Department of Pathology, First Affiliated Hospital of Guangxi Medical University, Nanning, Guangxi Zhuang Autonomous Region, People's Republic of China; ^2^ Department of Ultrasonography, First Affiliated Hospital of Guangxi Medical University, Nanning, Guangxi Zhuang Autonomous Region, People's Republic of China; ^3^ Department of General Surgery, First Affiliated Hospital of Guangxi Medical University (West), Nanning, Guangxi Zhuang Autonomous Region, People's Republic of China

**Keywords:** miR-204-5p, TCGA, prognostic, malignancies, bioinformatics

## Abstract

**Purpose:**

The prognostic role of miR-204-5p (previous ID: miR-204) is varied and inconclusive in diverse types of malignant neoplasm. Therefore, the purposes of the study comprehensively explore the overall prognostic role of miR-204-5p based on high-throughput microRNA sequencing data, and to investigate the potential role of miR-204-5p via bioinformatics approaches.

**Materials and Methods:**

The data of microRNA sequencing and survival were downloaded from The Cancer Genome Atlas (TCGA), and the prognostic value of miR-204-5p was analyzed by using Kaplan-Meier and univariate cox regressions. Then a meta-analysis was conducted with all TCGA data and relevant studies collected from literature. Pooled hazard ratios (HRs) with 95% confidence intervals (CIs) were calculated. The prospective molecular mechanism of miR-204-5p was also assessed at a functional level with Gene Ontology (GO), Kyoto Encyclopedia of Genes and Genomes (KEGG), and protein-to-protein interactions (PPI) network.

**Results:**

From TCGA data, the prognostic value of miR-204-5p obviously varied among 20 types of cancers. The pooled HR was 0.928 (95% CI: 0.774–1.113, *P* = 0.386, 6203 cases of malignancies). For the meta-analysis based on 15 studies from literature, the pooled HR was 0.420 (95% CI: 0.306–0.576, *P* < 0.001, 1783 cases of malignancies) for overall survival (OS). Furthermore, the combined HR from both TCGA and literature was 0.708 (95% CI: 0.600–0.834, *P* < 0.001, 7986 cases of malignancies). Subgroup analyses revealed that miR-204-5p could act as a prognostic marker in cancers of respiratory system and digestive system. Functional analysis was conducted on genes predicted as targets (*n* = 2057) after the overlay genes from six out of twelve software were extracted. Two significant KEGG pathways were enriched (hsa04360: Axon guidance and hsa04722: Neurotrophin signaling pathway). PPI network revealed some hub genes/proteins (CDC42, SOS1, PIK3R1, MAPK1, PLCG1, ESR1, MAPK11, and AR).

**Conclusions:**

The current study demonstrates that over-expression of miR-204-5p could be a protective factor for a certain group of cancers. Clinically, the low miR-204-5p level could gain a predictive value for a poor survival in cancers of respiratory system and digestive system. The detailed molecular mechanisms of miR-204-5p remain to be verified.

## INTRODUCTION

MicroRNAs (miRNAs) are a class of non-coding RNAs with about 18–25 nucleotides. One of the mechanisms through which miRNAs regulate gene expression involves the base-pairing primarily with the 3′-untranslated regions (3′-UTRs), and more rarely with 5′-end of their target mRNAs through the “seed” sequences of the miRNAs [1]. The base-pairing can cause translational inhibition, mRNA destabilization and/or degradation. Hence, miRNAs can function as a switch and a fine-tuner of the gene regulatory network, which may consist of hundreds, even thousands of potential target genes [2]. Since the first miRNA Lin-4 was discovered, it has become increasingly clear that miRNAs exert its functions via various biological processes. Increasing evidence has also shown that imbalance of miRNA expression can lead to a wide variety of diseases, including inheriting diseases, obesity, cardiovascular diseases, anxiety disorder and cancers. Previous scientific evidence indicates that aberrant miRNAs expression exists in numerous cancers, and miRNAs participate in tumorigenesis and progression, functioning as oncogenes or tumor suppressors [3, 4]. Moreover, aberrant expression of miRNAs, being tissue-specific, can be detected both in tissue and body fluid. And the abnormal expression of miRNAs is apparently related to deviant cancerous outcomes, suggesting that miRNAs are potential substantial biomarkers of cancers [5–13].

MiR-204-5p (previous ID: miR-204), one of the most promising miRNAs, has a considerable impact on the tumorigenesis and progression of cancers through different mechanisms [14–16]. In recent years, a large number of studies have reported that the reduced expression of miR-204-5p correlated with worse survival of numerous cancers, including nasopharyngeal carcinoma (NPC), gastric cancer (GC), non-small cell lung cancer (NSCLC), breast cancer (BC), neuroblastoma, acute myeloid leukemia (AML), hepatocellular carcinoma (HCC) and oral squamous cell carcinomas(OSCC), which indicates that miR-204-5p serves as a tumor suppressor [17–26]. However, one study focusing on uterine corpus endometroid carcinoma (UCEC) revealed no significant association between miR-204-5p down-regulation and overall survival (OS) [16]. The correlation between miR-204-5p expression and OS of pancreatic cancer in the non-gemcitabine and gemcitabine groups was contrary [27]. And the prognostic value of miR-204-5p for colorectal cancer (CRC) was controversial [28–30]. So, opinions were split on the prognostic role of miR-204-5p based on all publications.

The aim of The Cancer Genome Atlas (TCGA) pilot project is to evaluate the value of large-scale multidimensional analysis of molecular characteristics in TCGA can deepen the understanding of the molecular foundation of different malignancies, including the information of the prognostic value of a certain marker in various cancers [31]. Therefore, we investigated the prognostic value of miR-204-5p in all cancers available based on TCGA data. We also conducted a meta-analysis and systematic review to clarify the comprehensive prognostic significance of miR-204-5p with a comparatively refined result by combining data of TCGA and literatures.

## RESULTS

### The expression and prognostic value of miR-204-5p of various types of cancer based on TCGA data

**Figure 1:**
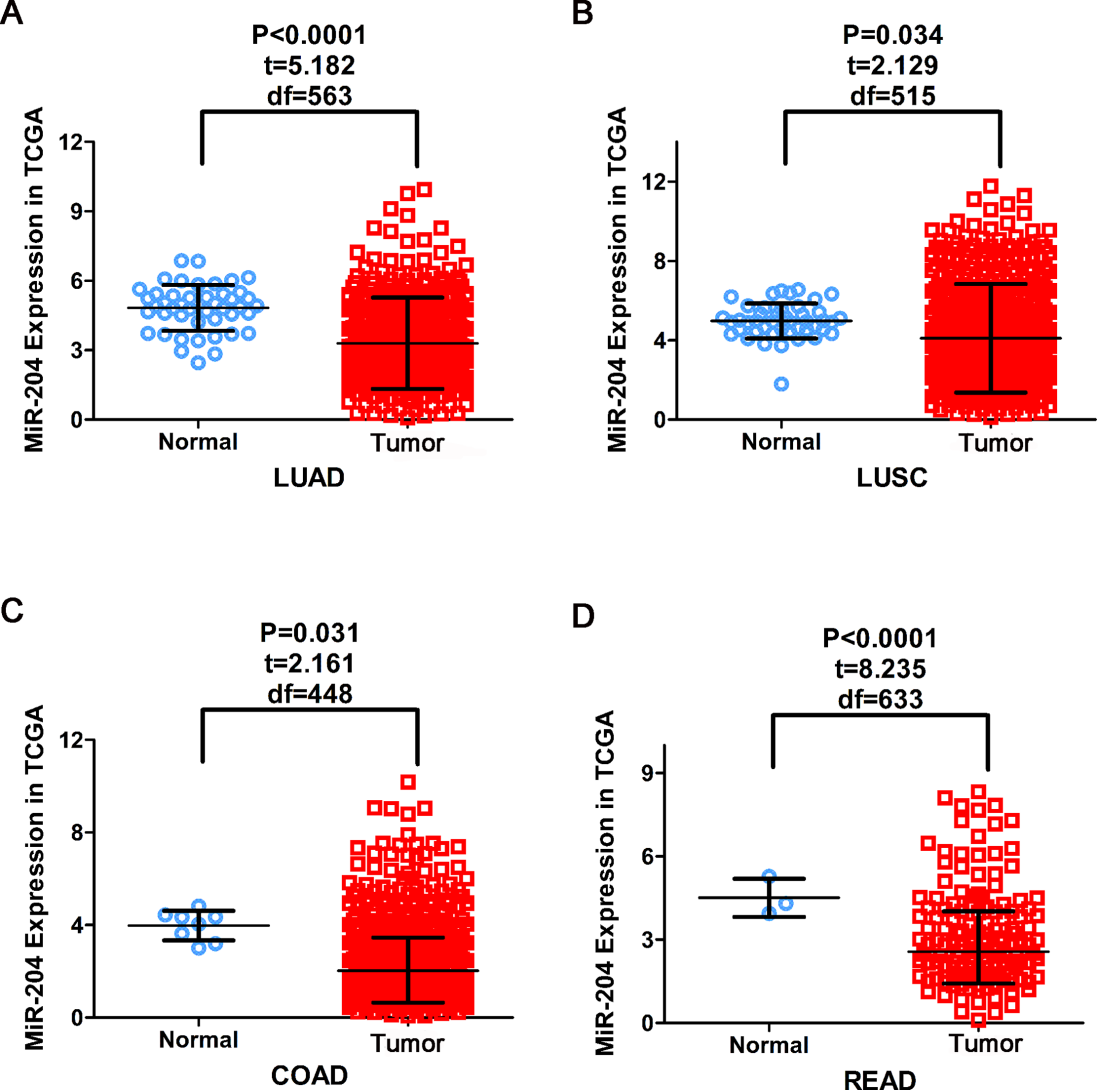
The expression of miR-204-5p in cancers in TCGA Down-regulation of miR-204-5p was detected in LUSC, LUAD, COAD and READ compared with corresponding non-cancerous tissues. LUSC (lung squamous cell carcinoma); LUAD (lung adenocarcinoma); COAD (colon adenocarcinoma); READ (rectum adenocarcinoma).

MiR-204-5p expression was analyzed in a total of 17 categories of malignancies and decreased miR-204-5p was detected in 13 cancers compared with corresponding non-cancerous tissues (*P* < 0.05, Figure 1, Supplementary Figures 1, 2).

**Figure 2:**
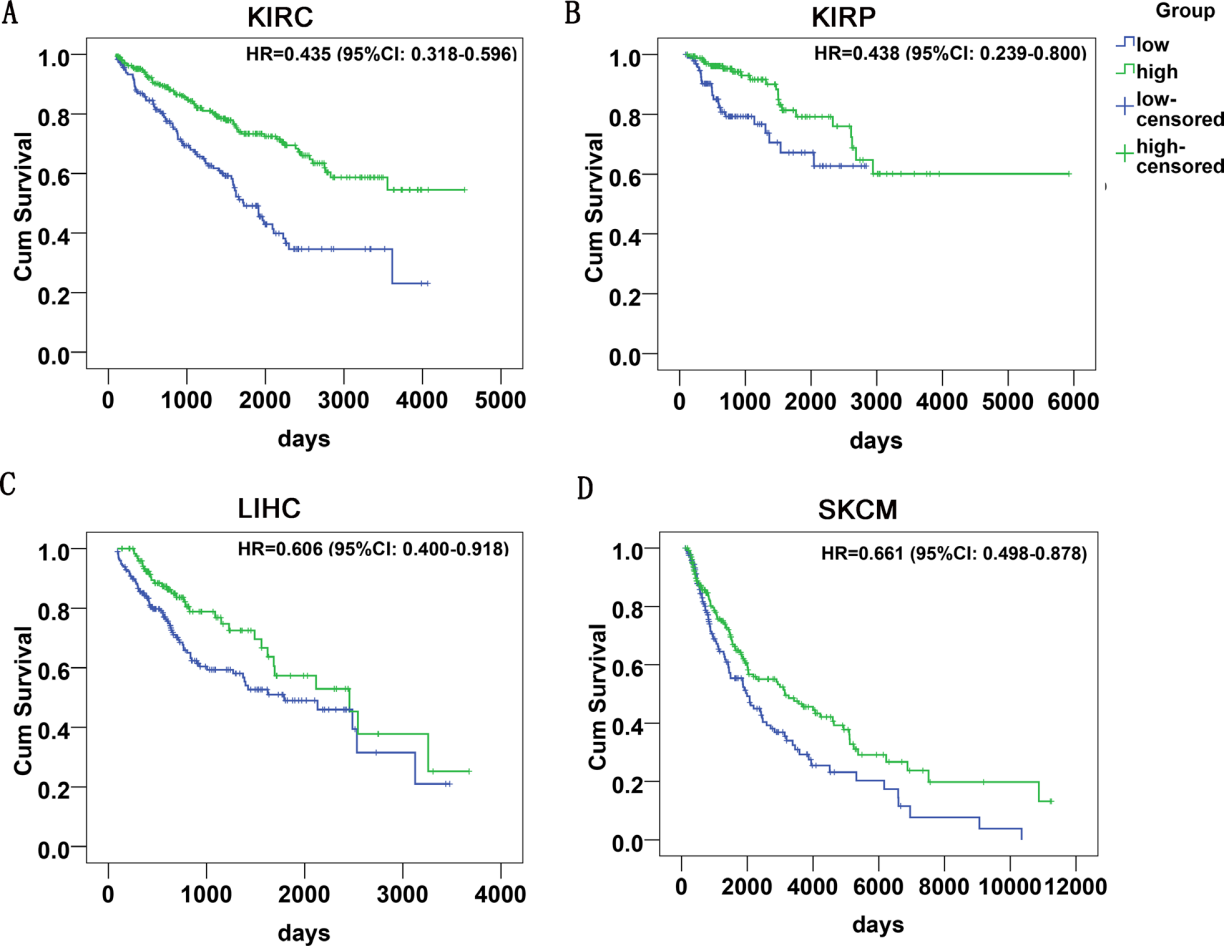
The survival curves of miR-204-5p in cancers in TCGA HR: hazard ratio; CI: confidence interval; KIRC: kidney renal clear cell carcinoma; KIRP: kidney renal papillary cell carcinoma; LIHC: liver hepatocellular carcinoma; SKCM: skin cutaneous melanoma.

**Figure 3:**
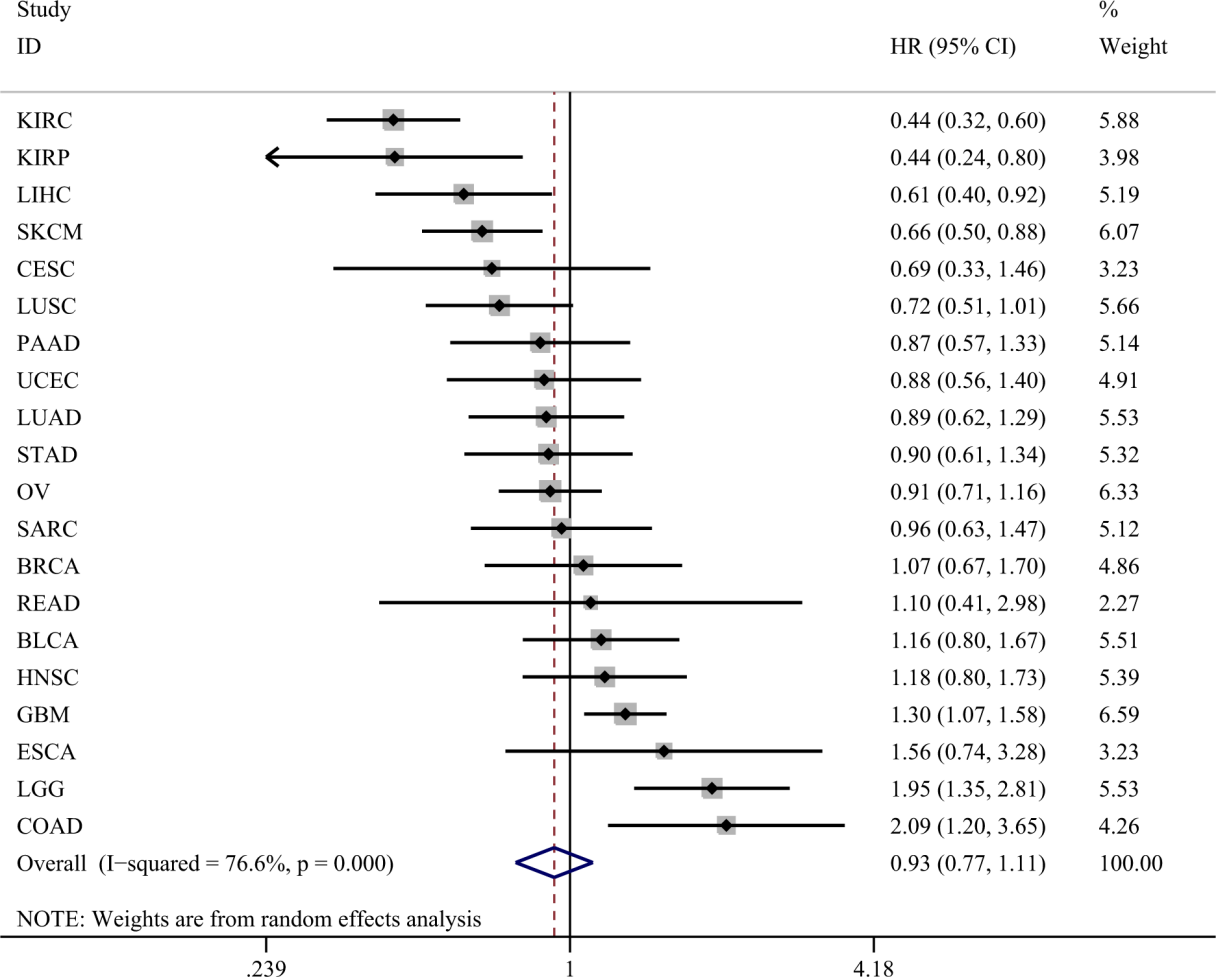
Forest plots of miR-204-5p expression and OS rate in 20 cancers in TCGA HR: hazard ratio; CI: confidence interval; KIRC: kidney renal clear cell carcinoma; KIRP: kidney renal papillary cell carcinoma; LIHC: liver hepatocellular carcinoma; SKCM: skin cutaneous melanoma; CESC: cervical squamous cell carcinama and endocervical adenocarcinoma; LUSC: lung squamous ce ll carcinoma; PAAD: pancreatic adenocarcinoma; UCEC: uterine corpus endometrial carcinoma; LUAD: lung adenocarcinoma; STAD: stomach adenocarcinoma; OV: ovarian serous cystadenocarcinoma; SARC: sarcoma; BRCA: breast invasive carcinoma; READ: rectum adenocarcinoma; BLCA: bladder urothelial carcinoma; HNSC: head and neck squamous cell carcinoma; GBM: glioblastoma multiforme; ESCA: esophageal carcinoma; LGG: brain lower grade glioma; COAD: colon adenocarcinoma.

Altogether, the survival data could be achieved from 20 categories of malignancies (Table 1). The Kaplan-Meier curves were shown in Figure 2 (Supplementary Figure 3). Furthermore, HR of miR-204-5p in each tumor was calculated with univariate cox regression analysis (Table 1). Distinct HRs were noted for various cancers. For instance, in kidney renal clear cell carcinoma (KIRC), the HR was 0.435 (95% CI: 0.318–0.596, *P* < 0.001, *n* = 477), and in colon adenocarcinoma (COAD), the HR was 2.089 (95% CI: 1.196–3.648, *P* = 0.01, *n* = 223). We intended to use a meta-analysis to pool the HR to probe an overall prognostic value of miR-204-5p in all cancers and the summarized HR was 0.928 (95% CI: 0.774–1.113, *P* = 0.422, 6203 cases of malignancies, Figure 3) with random-effects model (*P* < 0.001, I^2^ = 76.8%).

**Table 1:** Characteristics of the included studies for the overall survival (OS) analysis in TCGA

Cancers	Sample size	Sampling site	Detection	Cut off	Follow-up (days)	Outcome	Risk evaluation method	HR	95% CI		P
KIRC	477	Tissue	MicroRNA sequencing	Median	90–5925	OS	univariate	0.435	0.318	0.596	< 0.001
KIRP	270	Tissue	MicroRNA sequencing	Median	90–4537	OS	univariate	0.438	0.239	0.8	0.007
LIHC	324	Tissue	MicroRNA sequencing	Median	90–6408	OS	univariate	0.606	0.4	0.918	0.018
SKCM	400	Tissue	MicroRNA sequencing	Median	90–11252	OS	univariate	0.661	0.498	0.878	0.004
CESC	146	Tissue	MicroRNA sequencing	Median	90–3675	OS	univariate	0.692	0.328	1.459	0.333
LUSC	338	Tissue	MicroRNA sequencing	Median	90–4765	OS	univariate	0.717	0.507	1.014	0.06
PAAD	163	Tissue	MicroRNA sequencing	Median	90–3720	OS	univariate	0.869	0.569	1.327	0.516
UCEC	443	Tissue	MicroRNA sequencing	Median	90–7428	OS	univariate	0.885	0.56	1.397	0.599
LUAD	363	Tissue	MicroRNA sequencing	Median	90–2741	OS	univariate	0.894	0.62	1.29	0.549
STAD	249	Tissue	MicroRNA sequencing	Median	90–5651	OS	univariate	0.904	0.608	1.343	0.616
OV	422	Tissue	MicroRNA sequencing	Median	90–5481	OS	univariate	0.911	0.715	1.161	0.452
SARC	222	Tissue	MicroRNA sequencing	Median	90–5723	OS	univariate	0.961	0.627	1.471	0.854
BRCA	563	Tissue	MicroRNA sequencing	Median	90–3846	OS	univariate	1.065	0.669	1.695	0.79
READ	99	Tissue	MicroRNA sequencing	Median	90–7106	OS	univariate	1.102	0.407	2.985	0.849
BLCA	237	Tissue	MicroRNA sequencing	Median	90–5480	OS	univariate	1.158	0.801	1.674	0.436
HNSC	246	Tissue	MicroRNA sequencing	Median	90–5050	OS	univariate	1.178	0.8	1.734	0.407
GBM	500	Tissue	MicroRNA sequencing	Median	90–3881	OS	univariate	1.298	1.069	1.576	0.009
ESCA	64	Tissue	MicroRNA sequencing	Median	90–2532	OS	univariate	1.557	0.738	3.282	0.245
LGG	454	Tissue	MicroRNA sequencing	Median	90–6423	OS	univariate	1.952	1.353	2.814	< 0.001
COAD	223	Tissue	MicroRNA sequencing	Median	90–4270	OS	univariate	2.089	1.196	3.648	0.01

The patients from TCGA were selected with survival more than 90 days. HR: hazard ratio; CI: confidence interval; KIRC: kidney renal clear cell carcinoma; KIRP: kidney renal papillary cell carcinoma; LIHC: liver hepatocellular carcinoma; SKCM: skin cutaneous melanoma; CESC: cervical squamous cell carcinama and endocervical adenocarcinoma; LUSC: lung squamous cell carcinoma; PAAD: pancreatic adenocarcinoma; UCEC: uterine corpus endometrial carcinoma; LUAD: lung adenocarcinoma; STAD: stomach adenocarcinoma; OV: ovarian serous cystadenocarcinoma; SARC: sarcoma; BR CA: breast invasive carcinoma; READ: rectum adenocarcinoma; BLCA: bladder urothelial carcinoma; HNSC: head and neck squamous cell carcinoma; GBM: glioblastoma multiforme; ESCA: esophageal carcinoma; LGG: brain lower grade glioma; COAD: colon adenocarcinoma.

### Meta-analysis results of literature

### Eligible studies of the meta-analysis based on literature

A total of 841 records were preliminarily identified according to the search strategy, and 91 articles remained candidates after titles and abstracts were screened. After the full text of the rest 91 studies was checked, 74 records were excluded due to absence of necessary information. Consequently, a total of 14 papers conformed to our selection criteria and one study, which was performed by our group to investigate the relationship between miR-204-5p and survival of HCC, were finally accepted in this research, and the publication time were between 2012 and 2016 [17–26, 32]. Papers evaluating the risk of survival according to different clinical stages and different populations were listed twice in Table 2 [20, 28], which were regarded as two individual studies. The process of study selection was presented in a flow diagram (Supplementary Figure 4).

**Table 2:** Characteristics of the included studies of literatures for the overall survival (OS) analysis

Study	Year	patient origin	Tumor type	N	Sample	Detection	Cut off	Follow-up (months)	Outcome	HR (95% CI)	Risk evaluation method	NOS score
Sümbül	2014	Turkey	CRC	66	Tissue	qRT-PCR	Median	0–80	OS	1.326 (0.625–2.815)	univariate	7
Yin	2014	China	CRC	94	Tissue	qRT-PCR	Quarter	0–80	OS	0.303 (0.147–0.622)	multivariate	8
Boisen	2014	USA	CRC	276	Tissue	TaqMan Human MicroRNA array	Mean	0–75	OS	0.850 (0.720–1.010)	multivariate	8
	2014	USA	CRC	127	Tissue	TaqMan Human MicroRNA array	Mean	0–75	OS	1.010 (0.850–1.210)	multivariate	8
Ma	2014	China	NPC	275	Tissue	qRT-PCR	Median	0–55	OS	0.323 (0.202–0.515)	survival curve	6
Peng	2014	China	NPC	50	Tissue	qRT-PCR	Mean	0–60	OS	0.271 (0.077–0.962)	survival curve	6
Shi	2014	China	NSCLC(I/II)	47	Tissue	qRT-PCR	Median	0–60	OS	0.348 (0.123–0.990)	survival curve	7 7
	2014	China	NSCLC(III/IV)	48	Tissue	qRT-PCR	Median	0–60	OS	0.169 (0.030–0.935)	survival curve	
Guo	2015	China	NSCLC	126	Plasma	qRT-PCR	Median	0–60	OS, DFS	OS0.584 (0.354–0.963)	multivariate	8
										DFS0.476 (0.233–0.980)	survival curve	
Sacconi	2012	Italy	GC	69	Tissue	qRT-PCR	Median	0–80	OS	0.256 (0.085–0.769)	multivariate	8
Chen	2016	China	GC	115	Serum	qRT-PCR	Median	0–60	OS	0.276 (0.123–0.354)	multivariate	8
Ge	2015	China	HCC	48	Tissue	qRT-PCR	Median	0–80	OS	0.427 (0.193–0.943)	survival curve	7
Our data	2016	China	HCC	70	Tissue	qRT-PCR	Median	0–68	OS	0.355 (0.093–1.357)	univariate	7
Li	2014	China	BC	129	Tissue	qRT-PCR	Median	0–55	OS, DFS	OS 0.418 (0.198–0.885) DFS 0.465 (0.221–0.980)	survival curve survival curve	6
Yu	2016	Taiwan	OSCC	60	Tissue	qRT-PCR	NA	0–60	OS	0.204 (0.062–0.667)	survival curve	7
Ryan	2012	USA	Neuroblastoma	143	Tissue	qRT-PCR	Median	0–60	OS	0.179 (0.075–0.426)	univariate	8
Butrym	2015	Poland	AML	40	Plasma	qRT-PCR	Median	0–55	OS	0.159 (0.028–0.917)	survival curve	6

NA not available; HR: hazard ratio; OS: overall survival; DFS: disease free survival; NOS: Newcastle-Ottawa Scale; CRC: colorectal cancer; NPC: Nasopharyngeal carcinoma; NSCLC: non-small cell lung cancer; GC: gastric cancer; HCC: hepatocellular carcinoma; BC: breast cancer; OSCC: oral squamous cell carcinomas; AML: acute myeloid leukemia.

TaqMan Human MicroRNA array: The TaqMan Human MicroRNA array A and B Cards Set v3.0 (Applied Biosystems) was used to quantify expression of human miRNAs with single determinations in the study reported by Boisen et al.

The primary characteristics of the 15 studies were shown in Table 2. A total of 1783 participants were recruited in this meta-analysis, from Turkey, China, Italy, USA and Poland, respectively. Cancer types covered CRC, NPC, NSCLC, GC, HCC, Neuroblastoma, AML, OSCC and BC. Among the 15 articles, there were 3 studies (*n* = 563) for CRC; two studies (*n* = 325) for NPC, three studies (*n* = 221) for NSCLC, two studies for GC (*n* = 184) and two studies for HCC (*n* = 118). In the rest of studies, BC, GC, OSCC, neuroblastoma, HCC and AML were mentioned once only. All included studies measured miR-204-5p in tissues except two studies in plasma [21, 24]. Quantitative real-time PCR (qRT-PCR) was performed for 14 studies, and TaqMan Human MicroRNA array was used for only one study by Boisen et al. [28].

### Association of miR-204-5p level with survival based on literature

**Figure 4:**
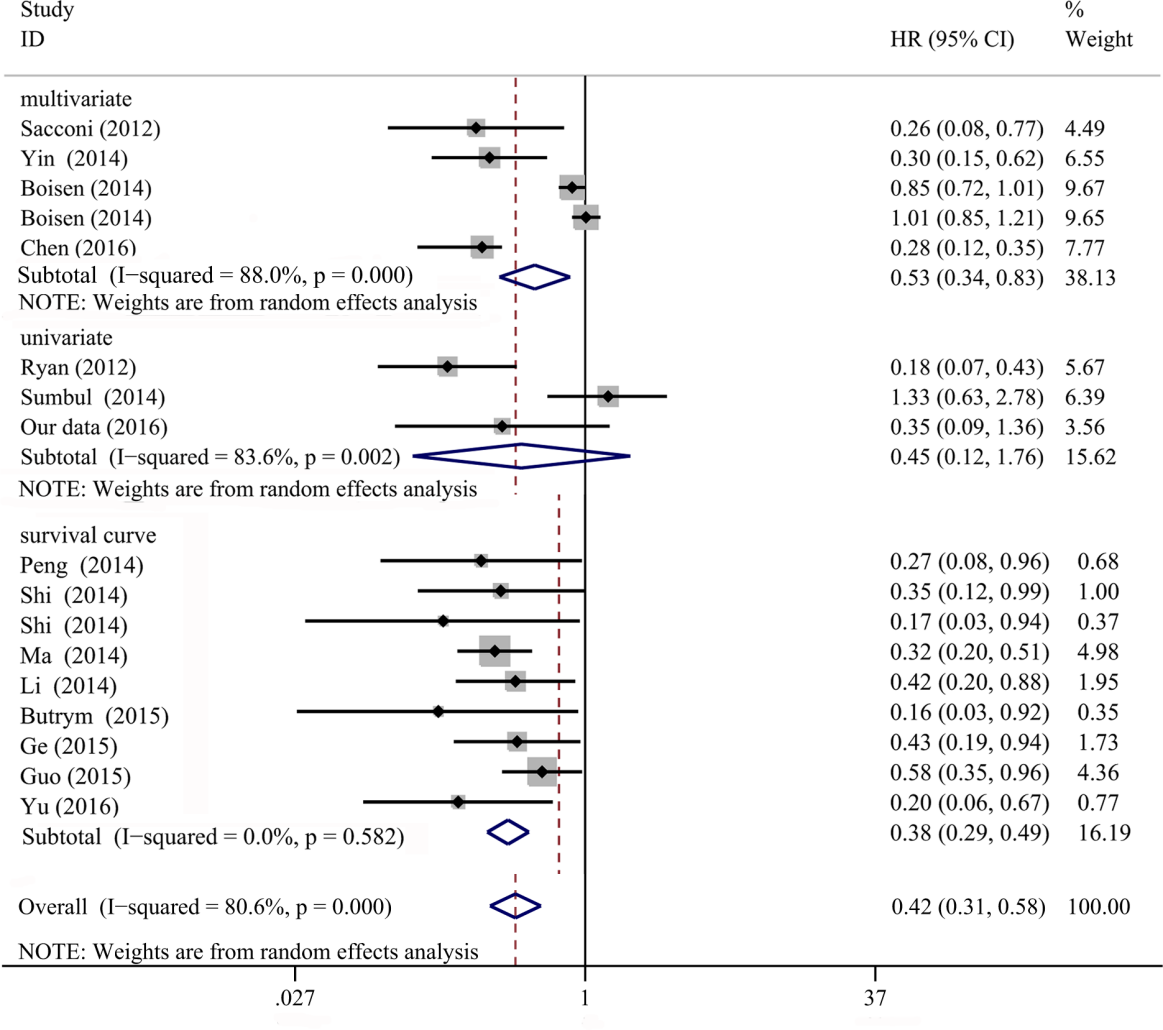
Forest plots of miR-204-5p expression and OS in cancers of literatures The mete-analysis of the 15 studies (17 cohorts, 1783 cases) showed a significant result that miR-204-5p was considered to be a protective factor (HR = 0.420, 95% CI: 0.306–0.576, *P* < 0.001).

**Figure 5:**
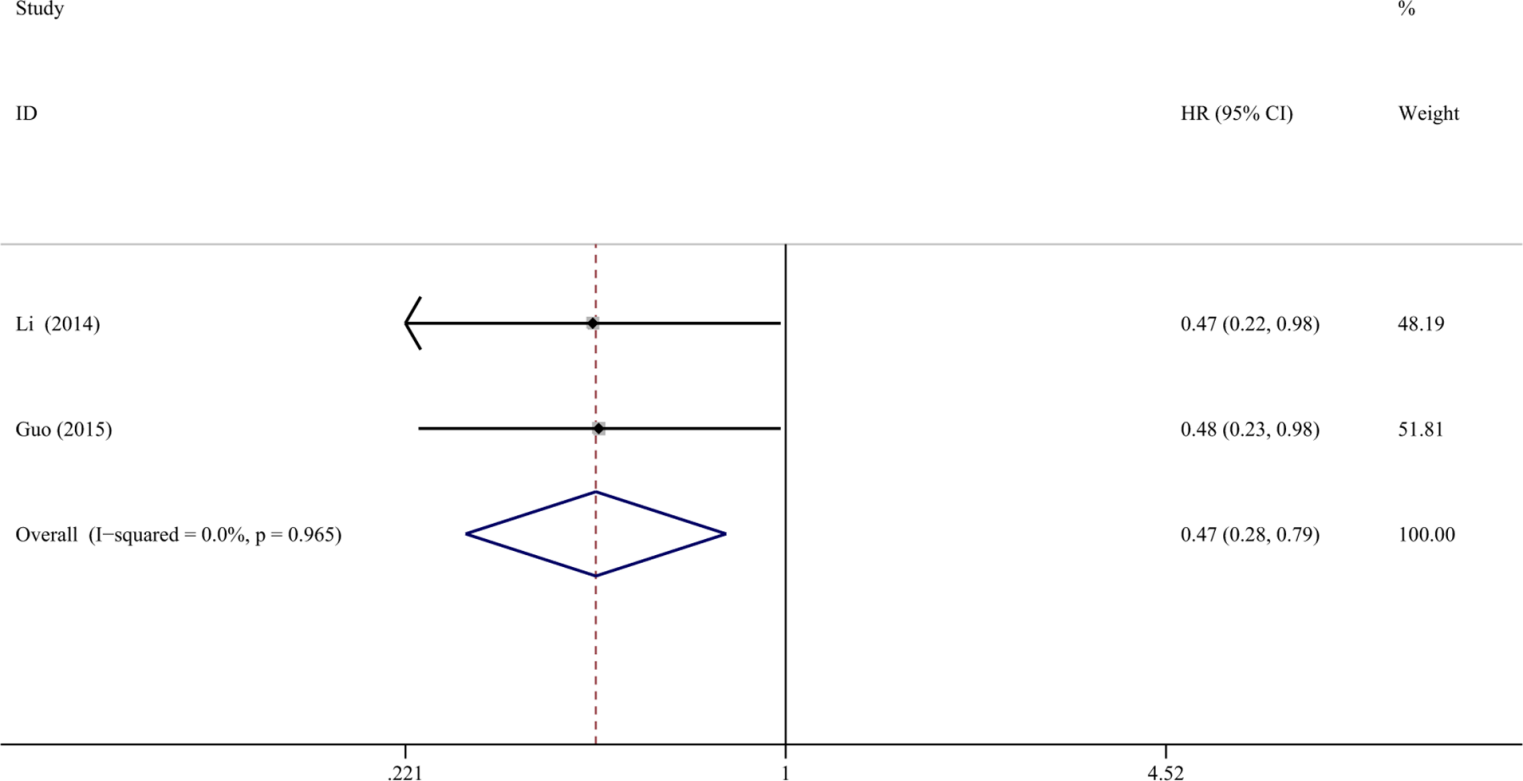
Forest plots of miR-204-5p expression and DFS in cancers of literatures Two studies (255 cases) were included for DFS, and the pooled HR was 0.471 (95% CI: 0.281–0.789, *P* = 0.004).

In total, 15 studies were included for OS and a random-effects model was applied due to the high level of heterogeneity (*P* < 0.001, I^2^ = 81%). The pooled HR of OS was 0.420 (95% CI: 0.306–0.576, *P* < 0.001, 1783 cases of malignancies, Figure 4). Moreover, two studies were analyzed for disease free survival (DFS) and the combined HR was 0.471 (95% CI: 0.281–0.789, *P* = 0.004, 255 cases of malignancies, Figure 5). Therefore, miR-204-5p could act as a protective factor for various cancers in general.

### Sensitivity analysis and publication bias

The sensitivity analysis revealed a stable combined HR after the removal of certain studies (Supplementary Figure 5). Begg's test and Egger's test revealed apparent publication bias among all the included studies for OS (Begg's test: *P* = 0.434, Egger's test: *P* < 0.001, Supplementary Figure 5). The result did not change at all after the adjustment of the trim and filling method, which suggested that the publication bias did not affect the conclusions obviously. Therefore, the HR of 0.420 indicated a significant correlation of poorer OS with low expression levels of miR-204-5p.

### Comprehensive meta-analysis combing TCGA and literature sources

**Figure 6:**
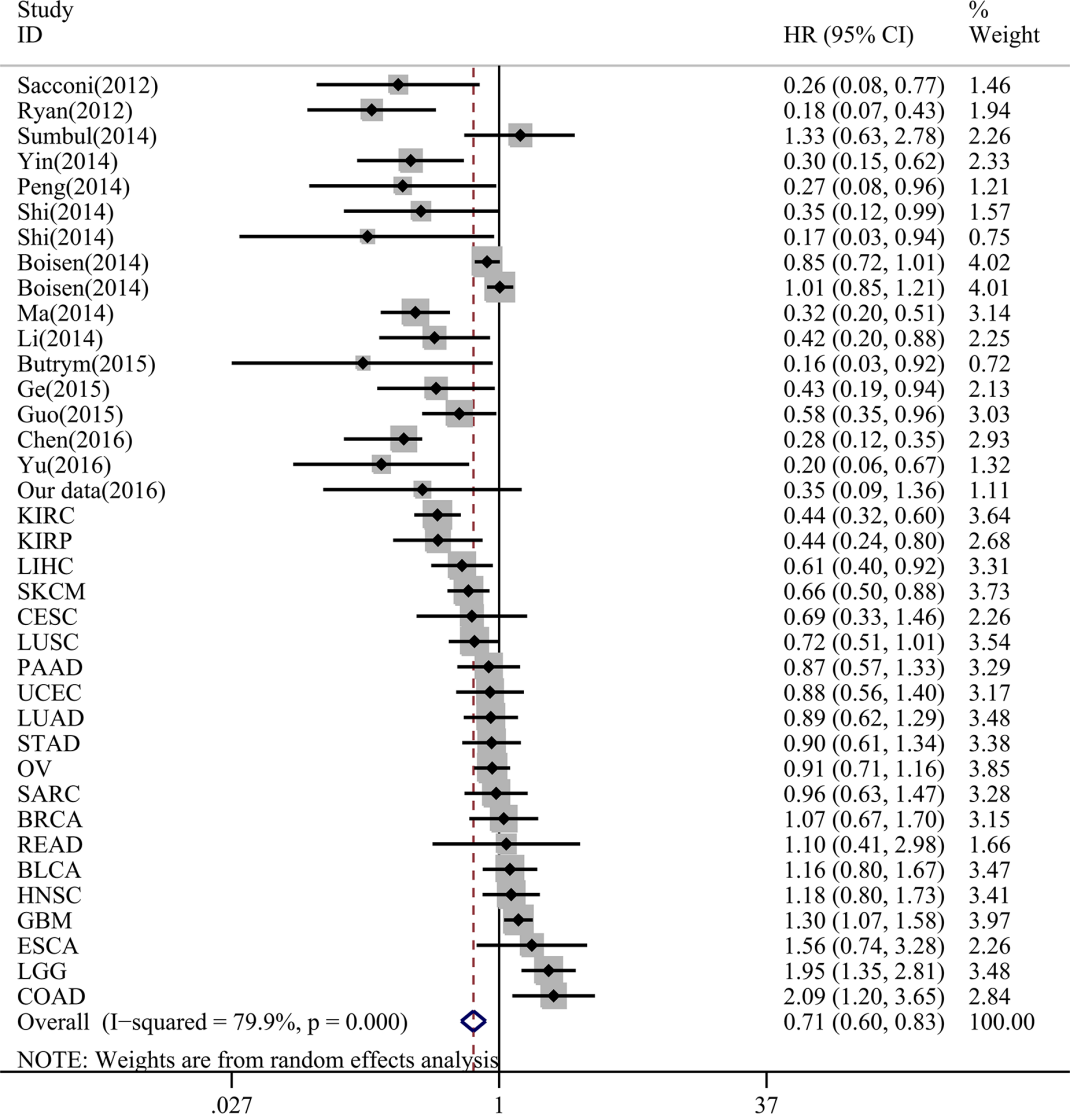
Forest plots of miR-204-5p expression and OS rate in TCGA and literatures KIRC: kidney renal clear cell carcinoma; KIRP: kidney renal papillary cell carcinoma; LIHC: liver hepatocellular carcinoma; SKCM: skin cutaneous melanoma; CESC: cervical squamous cell carcinama and endocervical adenocarcinoma; LUSC: lung squamous cell carcinoma; PAAD: pancreatic adenocarcinoma; UCEC: uterine corpus endometrial carcinoma; LUAD: lung adenocarcinoma; STAD: stomach adenocarcinoma; OV: ovarian s erous cystadenocarcinoma; SARC: sarcoma; BRCA: breast invasive carcinoma; READ: rectum adenocarcinoma; BLCA: bladder urothelial carcinoma; HNSC: head and neck squamous cell carcinoma; GBM: glioblastoma multiforme; ESCA: esophageal carcinoma; LGG: brain lower grade glioma; COAD: colon adenocarcinoma.

To better understand the comprehensive prognostic value of miR-204-5p based on both TCGA and literature data, we further performed a meta-analysis by combining all available information. The combined HR from both TCGA and literature was 0.708 (95% CI: 0.600–0.834, *P* < 0.001, 7986 cases of malignancies, Figure 6). This was calculated by using random-effects model (I^2^ = 80%, *P* < 0.001). The result further confirmed the predictive value of low miR-204-5p for a poor survival of various cancers.

**Figure 7:**
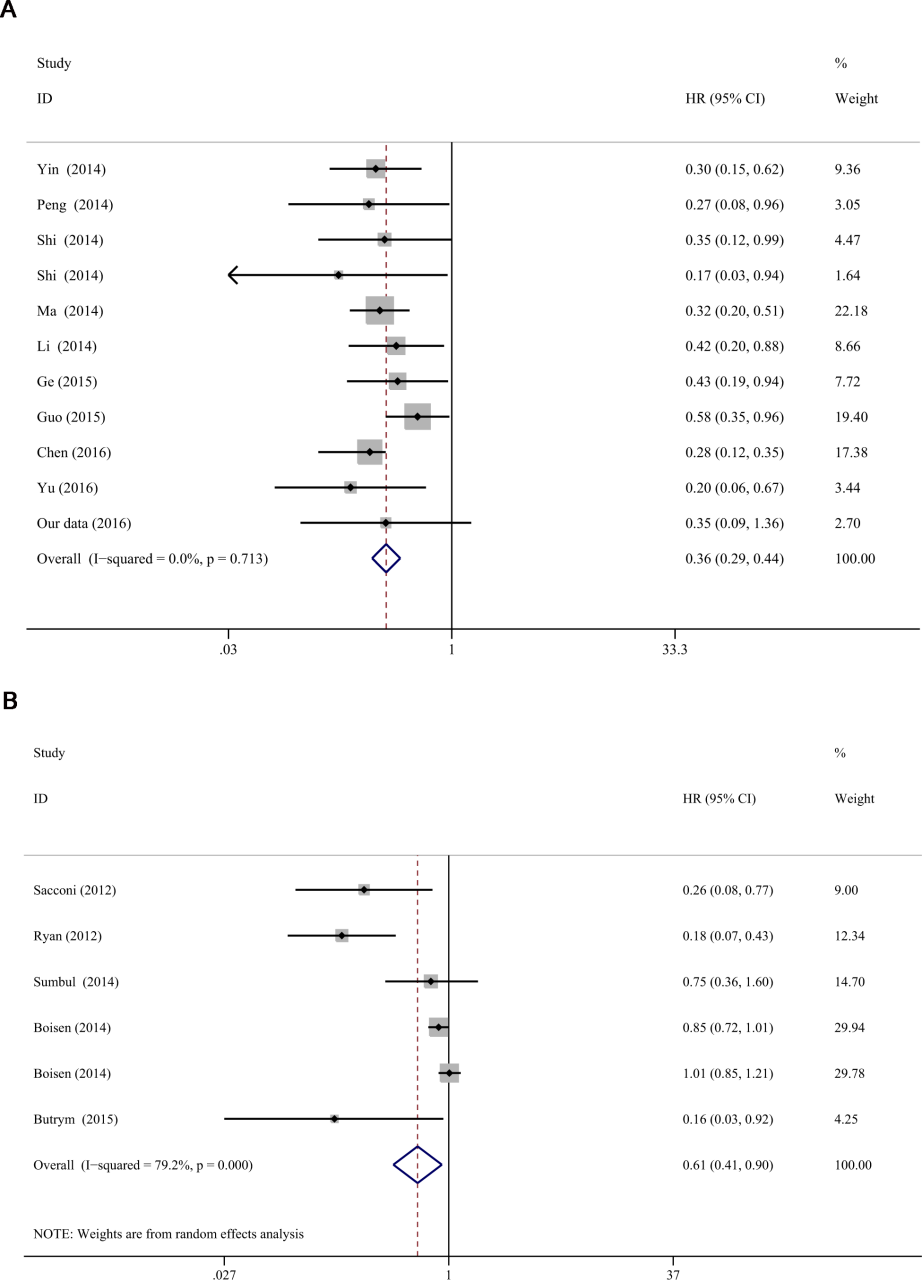
Subgroup analyses of region of miR-204-5p expression and OS in cancers (**A**) the included studies of China; (**B**) the included studies of non-China.

**Figure 8:**
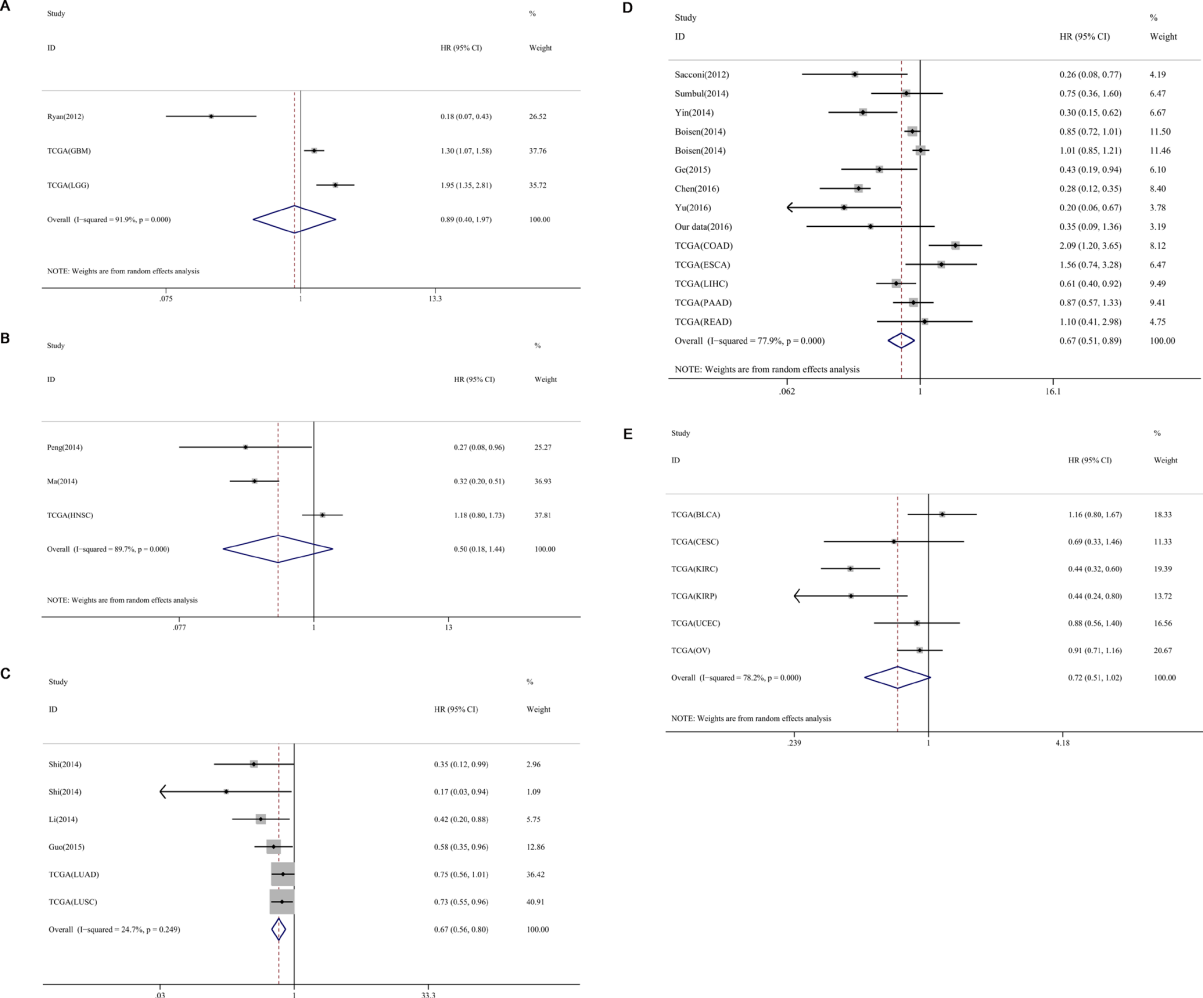
Subgroup analyses of cancer types of miR-204-5p expression and OS in cancers (**A**) nervous system; (**B**) head and neck squamous cell carcinoma; (**C**) respiratory system; (**D**) gastrointestinal system; (**E**) urinary and reproductive system. KIRC: kidney renal clear cell carcinoma; KIRP: kidney renal papillary cell carcinoma; LIHC: liver hepatocellular carcinoma; SKCM: skin cutaneous melanoma; CESC: cervical squamous cell carcinama and endocervical adenocarcinoma; LUSC: lung squamous cell carcinoma; PAAD: pancreatic adenocarcinoma; UCEC: uterine corpus endometrial carcinoma; LUAD: lung adenocarcinoma; STAD: stomach adenocarcinoma; OV: ovarian serous cystadenocarcinoma; SARC: sarcoma; BRCA: breast invasive carcinoma; READ: rectum adenocarcinoma; BLCA: bladder urothelial carcinoma; HNSC: head and neck squamous cell carcinoma; GB M: glioblastoma multiforme; ESCA: esophageal carcinoma; LGG: brain lower grade glioma; COAD: colon adenocarcinoma.

Different subgroup analyses were further performed on the basis of countries of the studies and types of cancers (Table 3). For the subgroup analysis for patient origin, only 15 published studies were analyzed. Eleven studies were from China (HR = 0.357, 95% CI: 0.286–0.445, *P* < 0.001, *n* = 1062, Figure 7A), and six studies were not from China (HR = 0.605, 95% CI = 0.410–0.895, *P* = 0.012, *n* = 721, Figure 7B). These included studies were split up into five groups: nervous system (HR = 0.888, 95% CI: 0.399–1.974, *P* = 0.771, *n* = 1097, Figure 8A), head and neck neoplasms (HR = 0.504, 95% CI: 0.177–1.400, *P* = 0.201, *n* = 571, Figure 8B), respiratory system (HR = 0.666, 95% CI: 0.557–0.797, *P* < 0.001, *n* = 1051, Figure 8C), digestive system disease (HR = 0.674, 95% CI: 0.510–0.892, *P* = 0.006, *n* = 1798, Figure 8D) and urinary and reproductive system (HR = 0.720, 95% CI: 0.505–1.025, *P* = 0.068, *n* = 1552, Figure 8E).

**Table 3:** Subgroup analysis of HR in overall survival (OS)

Analysis	No. of studies	No.of patients	HR(95% CI)	*P* value	Model	Heterogeneity	
						*I*^2^ (%)	*P*
Subgroup1: patient origin							
China	11	1062	0.357 (0.286–0.445)	< 0.001	Fixed-effect	0.0%	0.713
Non-China	6	721	0.605 (0.410–0.895)	0.012	Random-effect	79%	< 0.001
Subgroup 2 tumor types							
Nervous system	3	1097	0.888 (0.399–1.974)	0.771	Random-effect	92%	< 0.001
Head and neck squamous cell carcinoma	3	571	0.504 (0.177–1.440)	0.206	Random-effect	90%	< 0.001
Respiratory system	6	1051	0.666 (0.557–0.797)	< 0.001	Fixed-effect	25%	0.249
Gastrointestinal system	14	1798	0.674 (0.510–0.892)	0.006	Random-effect	78%	< 0.001
Urinary and reproductive system	6	1995	0.720 (0.505–1.025)	0.068	Random-effect	78%	< 0.001

### Targets prediction, functional enrichment analysis and PPI network

**Figure 9:**
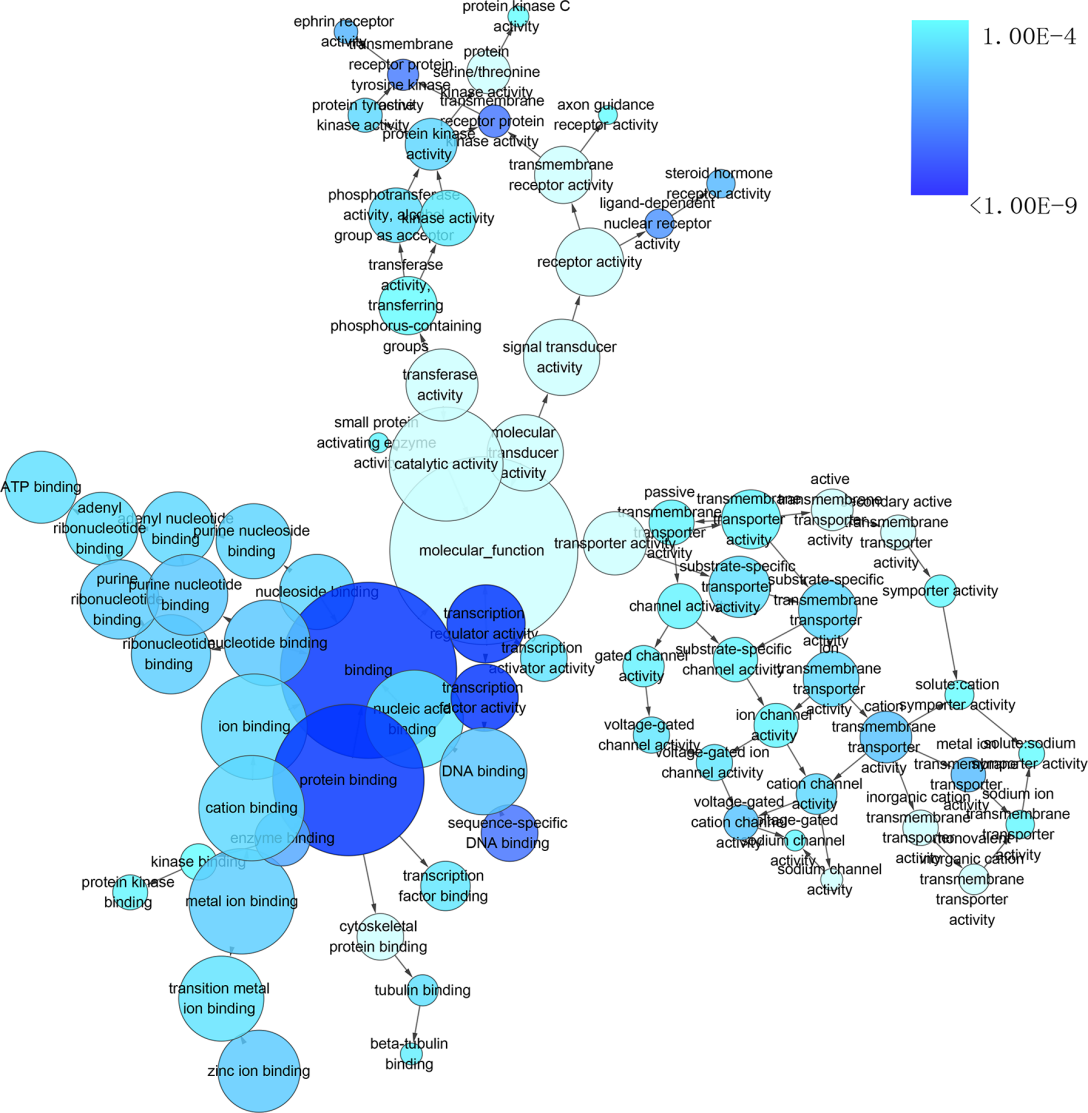
The network of enriched gene ontology (GO) terms of biological process The intensity of the color indicates *p*-value size (a smaller *P* value owns a deeper color), node refers to pathways and the node size is representative of the number of genes (the larger node owns more genes). The GO terms of biological process were presented with *P* < 0.0001.

**Figure 10:**
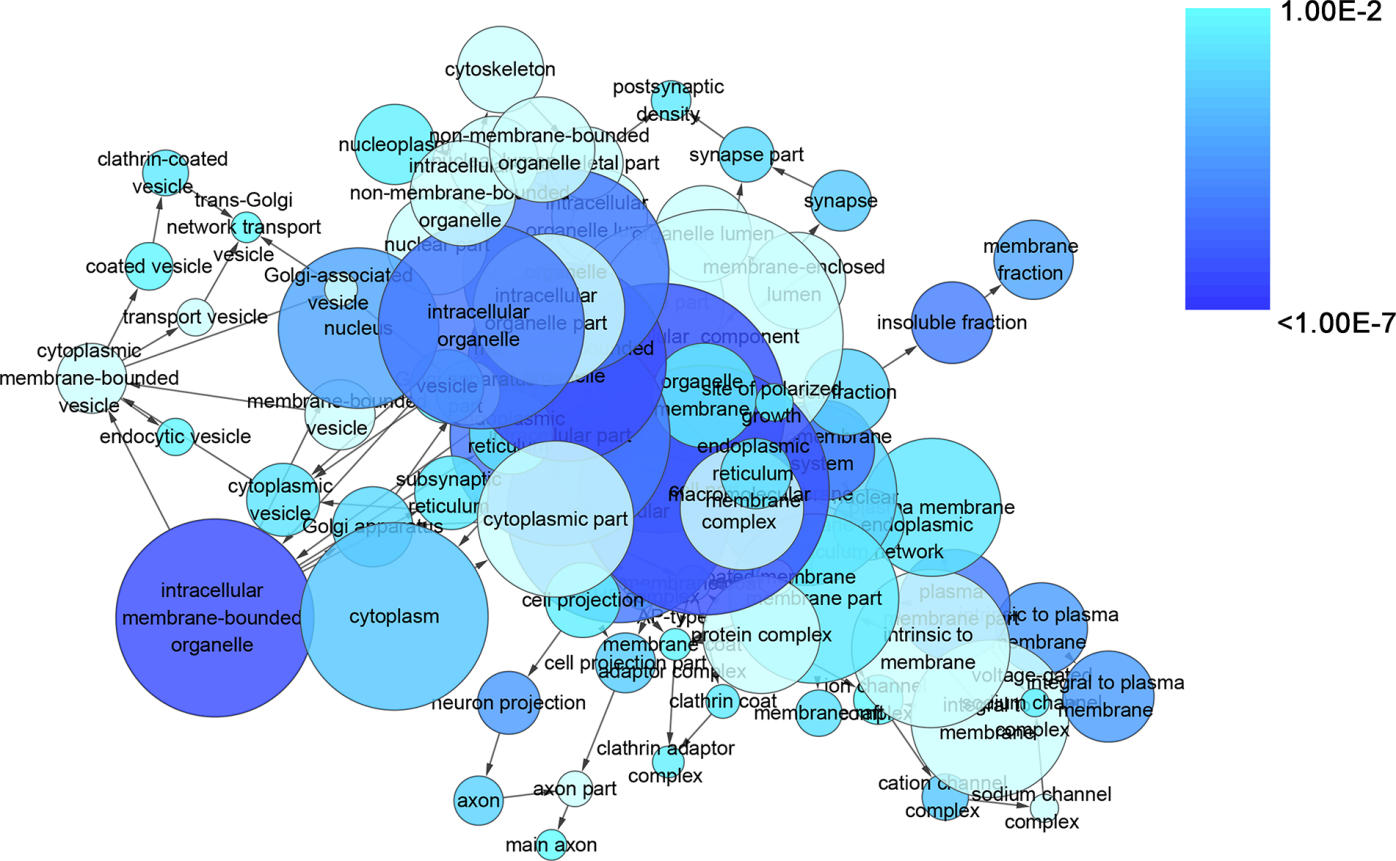
The network of enriched gene ontology terms of cellular component The intensity of the color indicates *p*-value size (a smaller *P* value owns a deeper color), node refers to pathways and the node size is representative of the number of genes (the larger node owns more genes). The GO terms of cellular component was showed with *P* < 0.01.

**Figure 11:**
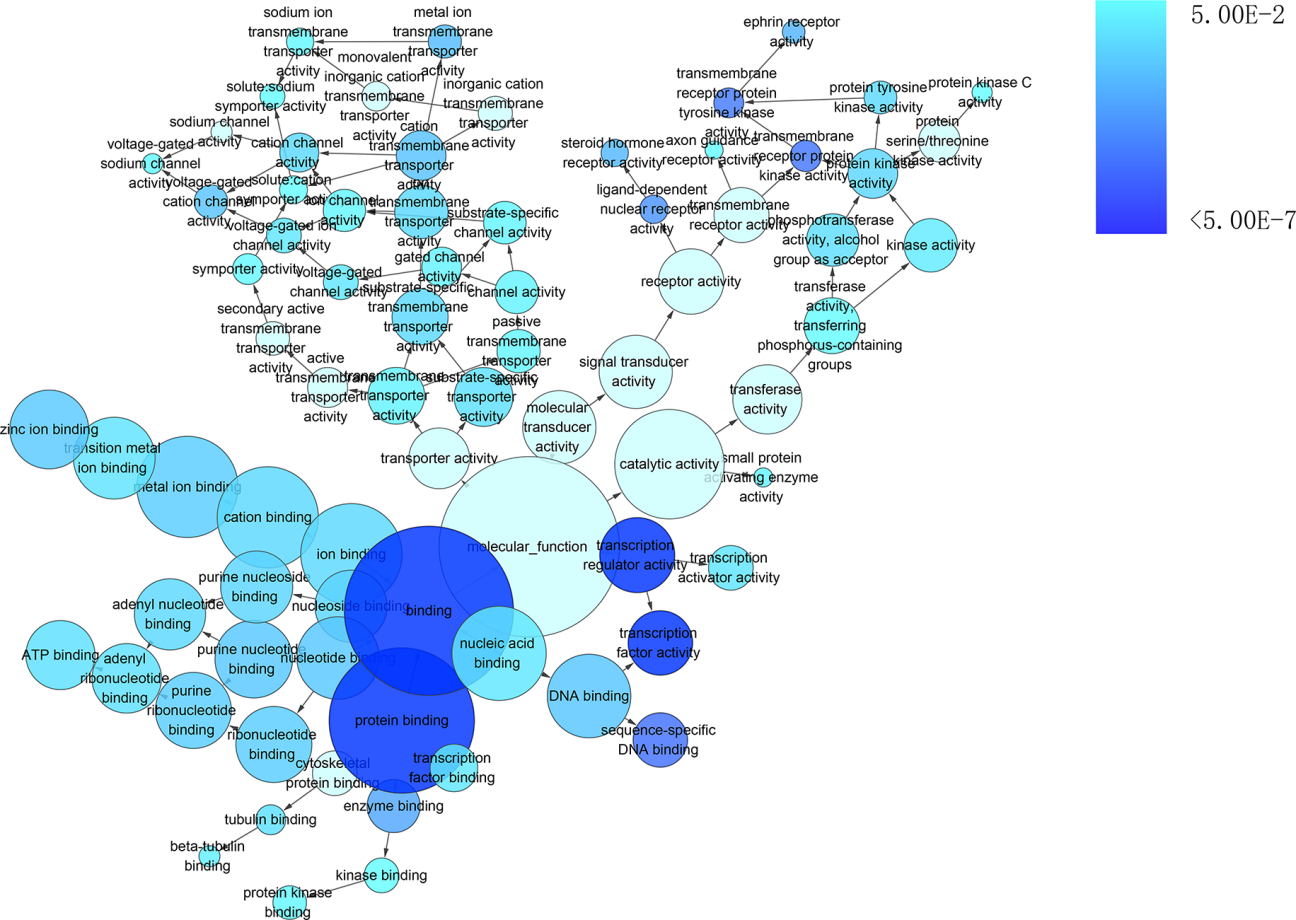
The network of enriched gene ontology terms of molecular function The intensity of the color indicates *p*-value size (a smaller *P* value owns a deeper color), node refers to pathways and the node size is representative of the number of genes (the larger node owns more genes). The GO terms of molecular function was showed with *P* < 0.05.

**Figure 12:**
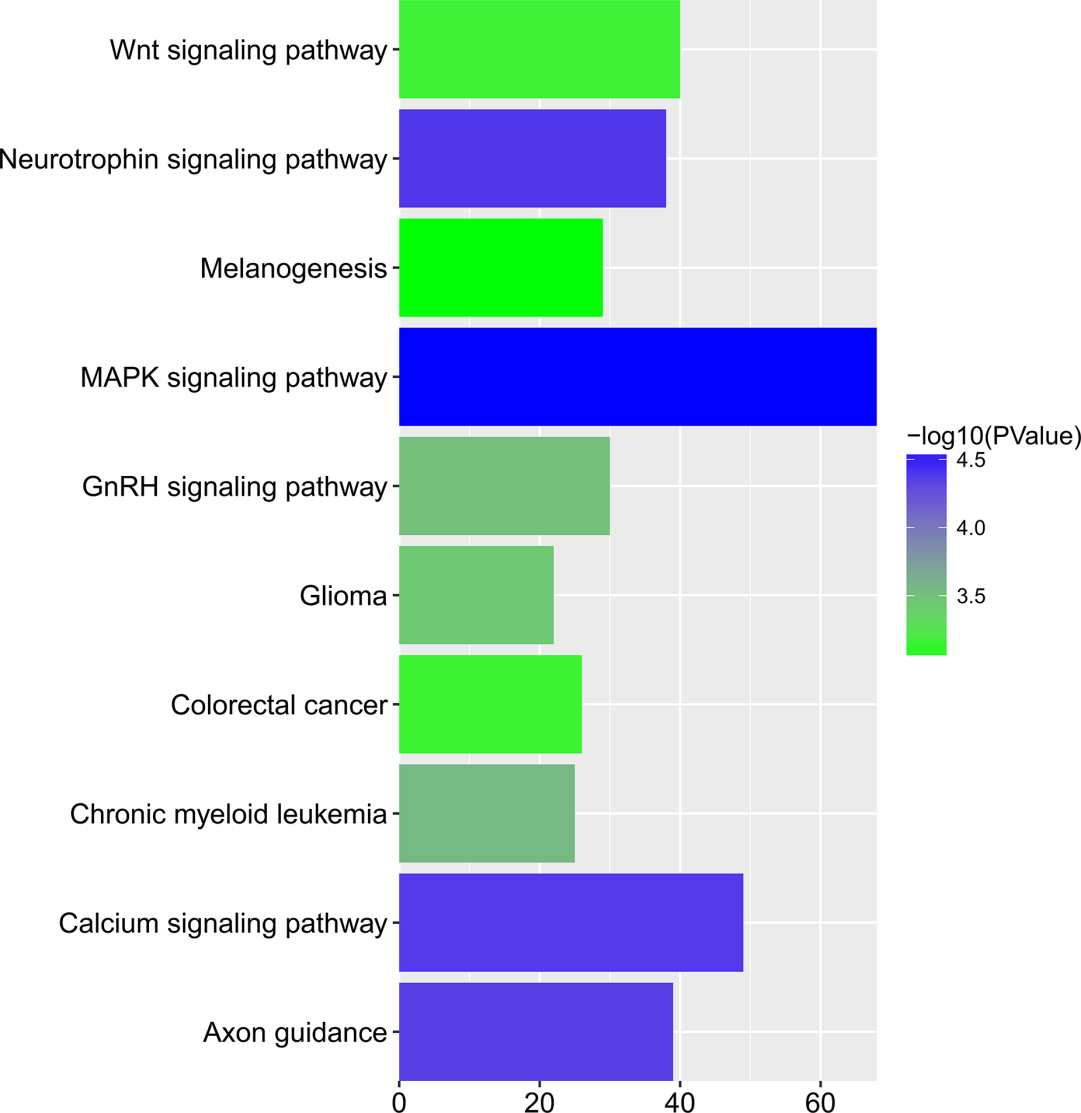
Top 10 KEGG pathways of miR-204a-5p prospective targets The bar schematic was drawn with R ggplot. High *P*-value was in blue and low *P*-value in green. Horizontal axis indicated the gene number in each pathway. MAPK signaling pathway was the most significant one among all 10 pathways, which contained 68 genes.

**Figure 13:**
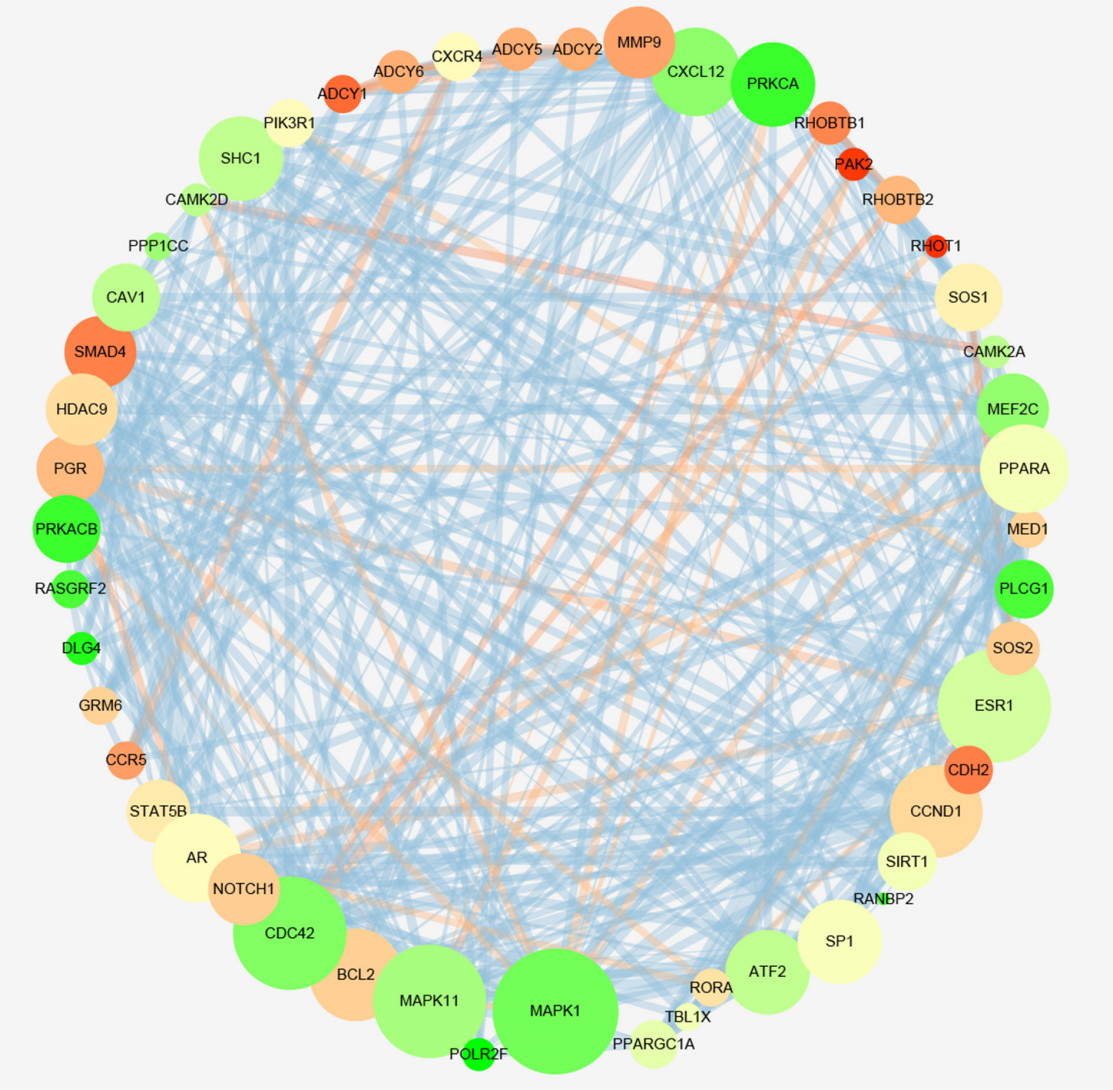
The network of protein-protein interaction (PPI) of miR-204a-5p prospective target genes Cytoscape (version 3.4.0) was used for the analysis of PPI network. The size of nodes expressed feature with a degree of connectivity. The color of nodes showed their clustering coefficient, representing the connectedness with high level in red and low level in green. High value to large size of edge was in red.

To gain insights into the prophetic role and molecular mechanism of miR-204-5p in various cancers, we predicted the target genes of miR-204-5p with 12 individual programs. Then, functional analysis was conducted on genes predicted as targets (*n* = 2057) after the overlay genes from six software were extracted. Gene ontology (GO) analysis unveiled that 29 pathways were associated with biological process (BP) (Figure 9), nine pathways with cellular component (CC) (Figure 10), and four pathways with molecular function (MF) (Figure 11), respectively (FDR < 0.05). The top four pathways of BP, CC and MF enriched functional analyses were listed in Table 4. Furthermore, two KEGG pathways were enriched (hsa04360:Axon guidance, hsa04722: Neurotrophin signaling pathway, FDR < 0.05, Table 5, Figure 12). No Panther pathway was achieved with FDR < 0.05. At last, PPI network of the target genes was shown in Figure 13. In this network, eight hub gene/proteins with more than 30 interactions with other gene/proteins were determined, including cell division CDC42, SOS1, PIK3R1, MAPK1, PLCG1, ESR1, MAPK11 and AR.

**Table 4:** The gene ontology (GO) analysis of the potential targets of miR-204-5p

ID	Term	FDR	Genes
	BP		
GO:0030182	Neuron differentiation	3.21E-07	ALS2, NRP2, NRP1, CNP, GRIN3A, RORA, PAX2, PRKG1, GDNF, CXCL12
GO:0048666	neuron development	5.90E-07	ALS2, NRP2, NRP1, CNP, GRIN3A, PAX2, PRKG1, GDNF, CXCL12, ZFP91
GO:0031175	neuron projection development	2.27E-06	NRP2, ALS2, NRP1, CNP, GRIN3A, PAX2, PRKG1, CXCL12, GDNF, BDNF
GO:0048812	neuron projection morphogenesis	4.34E-05	NRP2, ALS2, NRP1, LPPR4, ADORA2A, ERBB3, CNP, PAX2, CXCL12, EPHB1
	MF		
GO:0003700	transcription factor activity	1.02E-05	MEF2C, MEF2A, HMX2, HMX1, THRB, STAT5B, RORA, PGR, TAF5L, HOXC8
GO:0030528	transcription regulator activity	2.00E-05	MEF2C, MEF2A, STAT5B, MED22, RORA, MXI1, PGR, TAF5L, CREB3L2, ZNF396
GO:0004714	transmembrane receptor protein tyrosine kinase activity	0.002602197	NRP2, RET, NRP1, ERBB4, EFNB3, ERBB3, EFNA3, EPHA10, EPHA1, EPHB1
GO:0043565	sequence-specific DNA binding	0.022779507	ISX, MEF2C, PPARA, HMX2, MEF2A, BACH2, ELF2, HMX1, FOXK1, THRB
	CC		
GO:0044459	plasma membrane part	4.98E-06	VAPA, IL6ST, RP2, EFNA3, SYT6, GRIN3A, ZNRF1, AQP2, ATP2B1, ATP2B4
GO:0005887	integral to plasma membrane	2.50E-04	MPZL1, KCNC4, IL6ST, SLC6A20, EFNA3, GRIN3A, TLR6, VIPR2, ATP2B1, ATP2B4
GO:0031226	intrinsic to plasma membrane	3.67E-04	MPZL1, KCNC4, IL6ST, SLC6A20, EFNA3, GRIN3A, TLR6, VIPR2, ATP2B1, ATP2B4
GO:0005626	insoluble fraction	0.00195022	ALS2, SEPT3, VAPA, HMGCR, VAPB, ADCY6, SYNCRIP, LEMD3, CNP, RAB1A

Three parts of GO analysis was included (BP:biological process; MF: molecular function; CC: cellular component). The enriched terms with FDR less than 0.05 of the top 4 were presented.

**Table 5:** KEGG pathway: the top 10 FDR from small to large order of KEGG pathway

ID	Term	FDR	Genes
hsa04360	Axon guidance	6.66E-04	NRP1, PLXNA2, EFNA3, PPP3R1, CXCL12, EPHB1, SEMA5A, CDC42, PAK7, EPHB6
hsa04722	Neurotrophin signaling pathway	0.008788241	YWHAZ, ZNF274, NFKBIE, CDC42, IRAK3, BDNF, MAP3K3, BCL2, SOS1, SOS2
hsa04010	MAPK signaling pathway	1.087388423	MEF2C, FGF5, FGF18, IL1R1, PPP3R1, CACNB1, ATF2, CDC42, BDNF, MAP3K3
hsa04012	ErbB signaling pathway	3.053736293	PRKCA, ERBB4, ERBB3, STAT5B, PRKCB, MAPK1, PAK7, CRKL, PLCG1, PAK2
hsa05214	Glioma	5.792493391	PRKCA, E2F3, PRKCB, MAPK1, CCND1, PLCG1, SOS1, SOS2, CAMK2D, PDGFRB
hsa04020	Calcium signaling pathway	7.877057658	ADCY1, GNA15, ADCY2, ERBB4, ADORA2A, ERBB3, PPP3R1, ATP2B1, ATP2B4, GRPR
hsa05220	Chronic myeloid leukemia	10.40115864	E2F3, TGFBR1, STAT5B, TGFBR2, SMAD4, BCL2L1, MAPK1, CCND1, CRKL, GAB2
hsa04720	Long-term potentiation	10.9789348	PRKCA, MAPK1, RPS6KA3, ADCY1, GRIN2B, CAMK2D, PPP3R1, GRIN2A, PRKACB, PPP1CC
hsa04910	Insulin signaling pathway	15.39204299	SOCS3, PRKAB2, HK2, PRKCI, ACACA, PDE3A, SOCS4, PPP1CC, PPARGC1A, PCK1
hsa04210	Apoptosis	16.17575426	IL1R1, PPP3R1, ENDOD1, BCL2L1, BIRC2, CASP10, IRAK3, TNFRSF10D, BCL2, RIPK1

## DISCUSSION

In the current study, we investigated the prognostic role of miR-204-5p based on TCGA data for individual cancers and achieved heterogeneous results. The pooled HR indicated that miR-204-5p could not predict the survival, which could be due to the small size of cases and simple detecting method of microRNA-sequencing. To explore the prognostic role of miR-204-5p in cancers, we further performed meta-analyses with literature, as well as the combination of literature and TCGA. Interestingly, low miR-204-5p level could have predictive value for a poor survival of specific types of cancers. To have a better comprehension of the potential mechanism of miR-204-5p, we performed functional analysis with predicted target genes and found that the signaling pathways of Axon guidance and Neurotrophin pathway were closely related to the function of miR-204-5p. Finally, PPI network also revealed several hub gene/proteins among all the predicted targets.

From TCGA, we analyzed the prognostic value of miR-204-5p in 20 classes of cancers with Kaplan-Meier and univariate cox regression analysis. The results showed that miR-204-5p might play apparently different roles in various malignancies. To acquire overall prognostic significance of miR-204-5p for all cancers, we performed a meta-analysis and found that the summarized HR was 0.928 (*P* > 0.05), which suggested that miR-204-5p could not act as a prognostic marker for all the cancers based on TCGA. Since TCGA data were obtained only from one single technique of microRNA sequencing, we further added information from literature to further explore the prognostic value of miR-204-5p. The pooled HR from literature was 0.420 (95% CI: 0.306–0.576, *P* < 0.001) for OS and 0.471 (95% CI: 0.281–0.789, *P* = 0.004) for DFS. Thus, down-regulation of miR-204-5p could have the potential to predict poorer survival in cancers. Interestingly, when we combined the data from both TCGA and the literature, the final HR was 0.708 (95% CI = 0.600–0.834, *P* < 0.001), which indicated that miR-204-5p could act as a protective factor for various cancers in general. Considering the heterogeneity among different cancers, we conducted subgroup analyses according to cancer types. The subgroup analyses revealed that low miR-204-5p could predict shorter survival in cancers of respiratory system and digestive system. Meanwhile, in cancers of respiratory system, we observed that miR-204-5p (miR-204) level was significantly decreased both in LUAD and LUSC tissues compared with non-cancerous tissues (both *P* < 0.05). The aberrant expression of miR-204-5p in cancers of respiratory system suggests that miR-204-5p participates in carcinogenesis and progression, which supports its prognostic role. Thus, significant down-regulatory expression of miR-204-5p contributes to its predictive value of prognosis in cancers of respiratory system. As for caners of digestive system, we observed similar trend of miR-204-5p expression in cancer tissues (COAD, ESCA, LIHC, PAAD and READ). But, the HRs of COAD, ESCA and READ are not as expected, which might be resulted from limited sample size and the other confounding factors, such as age, different treatments, etc. The confounding factors should be further investigated in future to better clarify the prognostic role of miR-204-5p. In a word, as a biomarker of prognosis, miR-204-5p could be clinically employed in cancers of respiratory system and digestive system based on the subgroup analyses.

In the subgroup analyses of the meta-analysis, we observed that low miR-204-5p expression predicted a worse survival in cancers of respiratory system, digestive system, but not in nervous system, head and neck neoplasms or urinary and reproductive system. The reasons may be that 1) miR-204-5p expressed heterogeneously in different cancers, 2) the regulatory mechanisms which miR-204-5p participated in may be cancer-specific, 3) the aberrant genes in different cancers may be inconsistent, since APC in colorectal cancer and p16 in glioma, which may lead to a different status of miR-204-5p in cancers.

After finding miR-204-5p gained the clinical significance to predict the prognosis of cancers, we wonder how miR-204-5p performs its function. A qualitative study reviewed the role of miR-204-5p in cancers, including its expression, regulation and biological functions, especially focusing on its role in tumor development and progression. In this review, Li et al. summarized the targets identified in previous studies and showed the regulatory mechanisms of miR-204-5p to promote tumor development and progression, such as induction of cell apoptosis, inhibition of epithelial-mesenchymal transition, control of long non-coding RNA, acting as a suppressor in cancer stem cell and increasing sensitivity of chemotherapy. Several targets of miR-204-5p have been confirmed in various cancers, such as EZRIN [33, 34], BCL-2 [23], FOXC1 [35], MEIS1 [36], RAB22A [37], NAUKI [20], SIRT1 [38], LC3B [39], PRKR-Like ER Kinase (PERK) [40], MX1 and TXNIP [41], snai1 [42], FOXM1 [43], ATF2 [44], JAK2 [45], etc. Notwithstanding, a single miRNA always functions through numerous targets, even an extremely large gene network. For a better comprehension of the potential signaling pathway and gene network of miR-204-5p, target genes were predicted by 12 softwares. Next, the bioinformatics studies of the functional pathway category and integrated network were performed, including GO, KEGG, Panther enrichment analysis and PPI. Interestingly, the top three terms in KEGG analysis were all closely related to tumorigenesis and tumor progression, including “Axon guidance” [46, 47], “Neurotrophin signaling pathway” [48, 49] and “MAPK signaling pathway” [50]. Among all the potential targets, PPI revealed that several hub genes were strongly associated with different process of malignancies, including CDC42 [51], SOS1 [52], PIK3R1 [53], MAPK1 [54], PLCG1 [55], ESR1 [56], MAPK11 [57] and AR [58].

In tumors, differentially-expressed microRNAs were obvious in plasma; thus, it was non-invasive and economical to detect the plasma rather than the tissue [59]. Unfortunately, few studies, to date, have evaluated the prognostic value of miR-204-5p in plasma [21, 24]. More studies focusing on other cancers are needed to test plasma detection of miR-204-5p efficiency.

Furthermore, experimental studies as well as clinical researches have been launched to investigate the relationship of miR-204-5p expression difference with chemotherapy sensitivity [19, 22–24]. Inspiringly, it was reported that miR-204-5p might increase sensitivity of some chemotherapy drugs in gastric cancer, neuroblastoma, and colorectal cancer [19, 23, 29].

However, our results should be interpreted meticulously due to the following limitations. Firstly, the publication bias of OS existed as the value of Egger's test pointed out. But the publication bias did not lead to different conclusion according to adjusted results by trim and fill method, which suggested our meta-analysis results were relatively robust. Secondly, the number of eligible studies, especially from literatures (*n* = 15), was relatively small, which prevented us from drawing convincing conclusions. Thirdly, the inconsistency of the cut off values may influence our findings. Fourthly, HRs were extracted from the Kaplan-Meier survival curves, which could unavoidably result in slight statistical errors. Fifthly, another possible bias could result from the fact that some negative findings could not be accepted for publication in a journal. Sixthly, we did not perform any experiments to validate the clinical role of miR-204-5p, including real-time quantitative polymerase chain reaction (RT-qPCR) or fluorescence *in situ* hybridization (FISH) with tissue or blood samples, especially for those cancers only containing miRNA-sequencing data from TCGA. Seventhly, no *in vitro* or *in vivo* functional experiments have been performed to test the predicting target genes and the possible molecular mechanism of miR-204-5p. Finally, different detecting methods of miR-204-5p may lead to potential heterogeneity among the combined studies, even though the random effect model was used to abate the effect of heterogeneity.

Most recently, a hypothesis termed competing endogenous RNA (ceRNA), which is closely related to miRNA, has grabbed researchers’ attention. The core of this hypothesis is that mRNA, pseudogenes, long noncoding RNA (lncRNA), or other molecules, which have the same miRNA response element (MRE), could ‘sponge’ the miRNA competitively, then perform their biological function [60, 61]. MiR-204-5p has also been studied in this hypothesis. For instance, MALAT1 could upregulate the expression of miR-204-5p target gene SLUG through competitively ‘sponging’ miR-204-5p to form a branch of the MALAT1/miR-204/SLUG pathway to regulate the progression of lung adenocarcinoma [60]. Similarly, another lncRNA, UCA1 could sponge endogenous miR-204-5p and suppress its activity via targeting CREB1 in CRC [62]. Furthermore, the axis of NEAT1/miR-204-5p/ZEB1 was confirmed in nasopharyngeal carcinoma [63]. In the future, further researches into the ceRNA network with miR-204-5p are required to be conducted in various cancers.

## MATERIALS AND METHODS

### TCGA data extraction

From the TCGA (http://cancergenome.nih.gov/), we downloaded and extracted the data of miR-204 expression from RNASeqV2 (level 3), as well as all clinical parameters, including survival status of all cancers in January 2016, through bulk download mode. MiR-204 expression data were presented as upper quartile normalized RSEM count estimates [39]. The miR-204 is immature pri-miRNA in TCGA data. Extracted data were used without further transformation.

### Meta-analyses for both TCGA data and literature

All TCGA data were used for meta-analysis and the detail was described below. We also performed a traditional meta-analysis with the information from publications.

### Search strategy and study selection of literature

Relevant studies were selected by comprehensive searching the online databases PubMed, Embase, web of science, Wiley Online Library, Cochrane library, Science Direct, Wan Fang, Chinese VIP, Chinese Biomedical Literature Database and CNKI up to January 1, 2017, independently. To achieve the maximum recall and precision of the articles, the keywords and entry words were as follows: (1) (miR-204 OR miRNA-204 OR microRNA-204 OR miR204 OR miRNA204 OR “miR 204” OR “miRNA 204” OR “microRNA 204” OR miR-204-5p OR miRNA-204-5p OR microRNA-204-5p); (2) (cancer OR tumor OR tumour OR neoplas* OR carcinoma OR sarcoma OR malignan*). In addition, some references of relevant articles were manually searched for further studies. If the articles adhered to the following criteria, they were included: (1) No language restriction of the publications was applied. (2) Patients with any type of cancers were included. (3) The relationships between miR-204-5p expression and prognosis were estimated. (4) The HRs and corresponding 95% CIs were provided from the original articles. If not provided, they could be calculated on the basis of the adequate data offered. (5) If the study of the same patient cohort was published twice or more, only the latest published and the most complete one was included. According to criteria below, some articles were excluded: (1) Reviews, letters, comments, case reports, editorials, expert opinions and conference abstracts without original data were excluded. (2) Articles of experimental *in vitro* or *in vivo* studies were excluded. (3) We also excluded the studies with no information on the relationship between miR-204-5p and survival. (4) Since we had downloaded and evaluated TCGA data by ourselves, those studies based on TCGA data were excluded. The detailed information of this meta were available in Supplementary Datas 1–4.

### Data extraction

Data were extracted in standardized data-collection forms individually. The following characteristics were recorded: (1) HR and corresponding 95% confidence interval (CI) of miR-204-5p for OS and DFS were collected directly from articles, calculated by SPSSv22.0 with raw data, or obtained from Kaplan-Meier survival curves with the method recommended by Parmar et al. and Tierney et al. [64, 65]. (2) Additional information: first author's surname, publication year, patient origin, sample size, tumor type, sampling site, risk evaluation method, assay of miR-204-5p measurement, cut off value, follow-up time.

### Statistical analysis

The median expression level was considered as cut-off to separate TCGA data into two groups-high level group and low level group. The survival was compared between the aforementioned two groups by using Kaplan-Meier survival curves, as well as the univariate cox regression analysis. In the current study, the data from TCGA and literature was firstly used for meta-analyses individually. Next, we further integrated the data from the literature with the TCGA to study the potential prognostic role of miR-204-5p based on all available studies using HRs. If 95% CI of HRs did not overlap 1, the relationship between miR-204-5p down-regulation and survival was identified as statistically significant. To investigate the outcomes of patients with various cancers or from different areas, we carried out subgroup analyses of prognosis based on the tumor types and patient origins. In terms of the statistical heterogeneity among the studies, Cochran's Q test as well as Higgins I^2^ statistic was applied. The heterogeneity of the pooled HRs was considered acceptable if *P* > 0.1, I^2^ < 50% (Cochrane Handbook for Systematic Reviews of Interventions Version 5.1.0). If a distinct heterogeneity was observed (*P* < 0.1, I^2^ > 50%), we employed the random-effects model to calculate pooled HRs. Otherwise the fixed-effects model would be selected. To assess the potential sources of heterogeneity, we conducted sensitivity analyses. After a single study was deleted and the rest studies were pooled again, sensitivity analysis was not only designed to examine the stability of outcomes, but also to further seek the sources of heterogeneity. Begg's funnel plot assessing the asymmetry qualitatively aimed at investigating the publication bias. Furthermore, Egger's linear regression test and “the trim and fill method” would offer the evidence for publication bias quantitatively [66, 67]. The meta-analysis was performed by Stata 12.0 (Stata Corp LP, College Station, USA).

### Targets prediction, functional enrichment and pathway enrichment analysis and protein-protein interaction (PPI) network

Targets of miR-204-5p were predicted by online target prediction tools, including DIANA-microTv4.0, DIANA-microT-CDS, miRanda-rel2010, mirBridge, miRDB4.0, miRmap, miRNAMap, doRiNA i.e., PicTar2, PITA, RNA22v2, RNAhybrid2.1 and Targetscan6.2. Functional enrichment (GO), Kyoto Encyclopedia of Genes and Genomes (KEGG) pathway enrichment analysis, as well as Panther pathway enrichment analysis were performed for the overlapping genes with the DAVID (https://david.ncifcrf.gov/). The statistical test of hypergeometric distribution to compute *P*-values was used to perform the current enrichment analysis. The analysis was further corrected by the Benjamini and Hochberg false discovery rate method for multiple hypothesis testing (α = 0.05). We only chose those terms with FDR value < 0.05 and ≥ 5 genes for analysis. Furthermore, we used STRING10.0 (http://string-db.org/, version 10.0) to construct a protein-protein interaction (PPI) network of the miR-204-5p target genes.

## CONCLUSIONS

Overall, miR-204-5p down-regulation could predict poorer survival of patients with several cancers based on TCGA data and publications. Further well-designed multi-institutional studies with larger population ought to be conducted to verify the exact prognostic significance of miR-204-5p in various cancers with more precise approaches like RT-qPCR and FISH. Moreover, the exact molecular mechanism of miR-204-5p also needs further investigation with *in vitro* and *in vivo* experiments.

## SUPPLEMENTARY MATERIALS FIGURES AND REFERENCES






