# Supplementary material for: The protective value of miR-204-5p for prognosis and its potential gene network in various malignancies: a comprehensive exploration based on RNA-seq high-throughput data and bioinformatics
Source: Oncotarget. 2017 Oct 23;8(62):104960–80. doi: 10.18632/oncotarget.21950 (PMC5739612; doi:10.18632/oncotarget.21950)
Supplement: Supplementary file 1 [file oncotarget-08-104960-s001.pdf]

The protective value of miR-204-5p for prognosis and its potential gene network in various malignancies: a comprehensive exploration based on RNA-seq high-throughput data and bioinformatics

SUPPLEMENTARY MATERIALS

Supplementary Data 1: The PRISMA Checklist of the meta-analysis. See Supplementary_Data_1

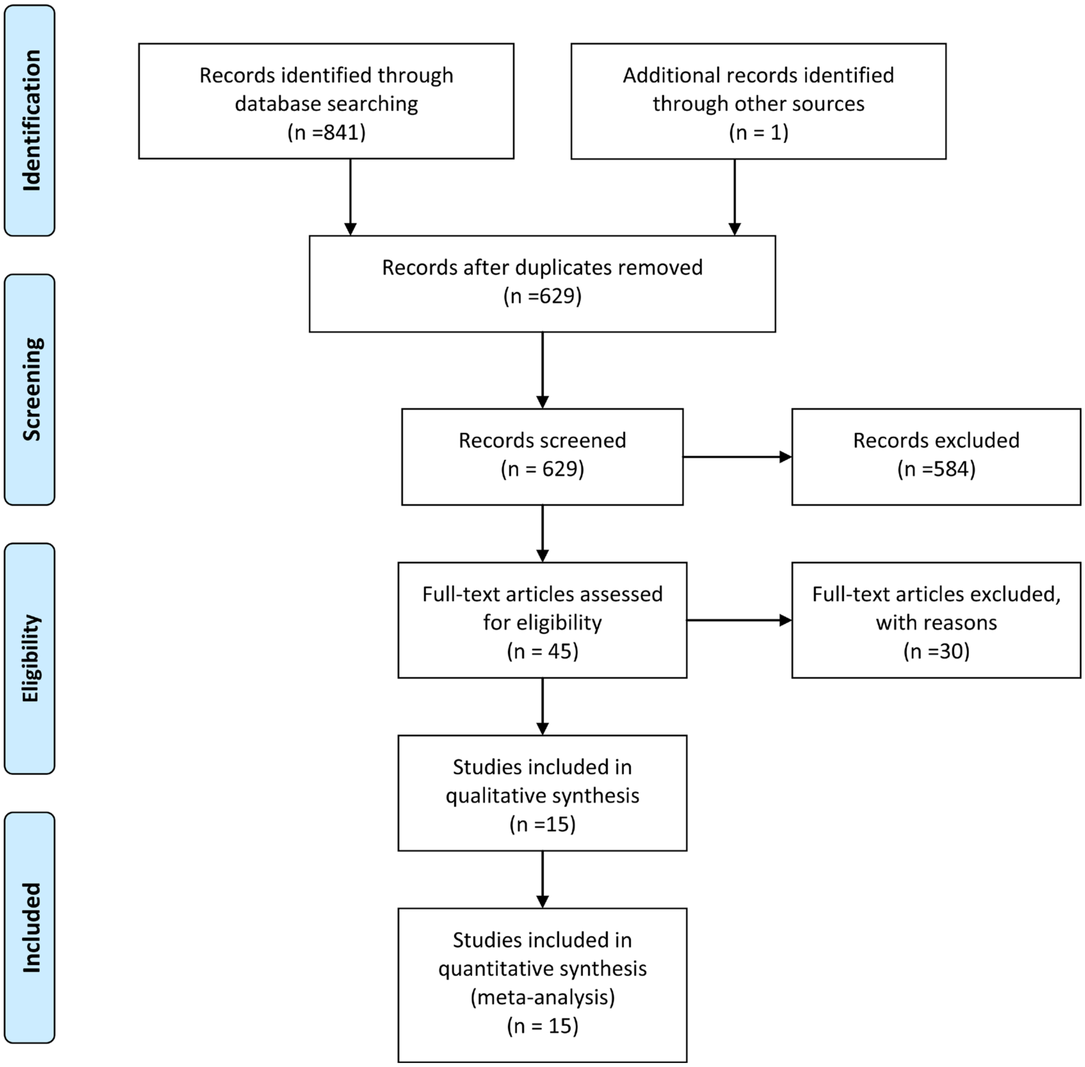

Supplementary Data 2: The PRISMA flow diagram of the meta-analysis.

Supplementary Data 3: Studies exclusion.

Studies didn't provide information for prognosis [1–20].

Trails using animals or cell lines or others but not samples of human [21–28].

Trails detected miR-204-5p combined other markers to estimate its prognostic significance [29].

Reviews [30].

REFERENCES

1. Shu X, Hildebrandt MA, Gu J, Tannir NM, Matin SF, Karam JA, Wood CG, Wu X. MicroRNA profiling in clear cell renal cell carcinoma tissues potentially links tumorigenesis and recurrence with obesity. Br J Cancer. 2016. https://doi.org/10.1038/bjc.2016.392.
2. Zhu J, Ma X, Zhang Y, Ni D, Ai Q, Li H, Zhang X. Establishment of a miRNA-mRNA regulatory network in metastatic renal cell carcinoma and screening of potential therapeutic targets. Tumour Biol. 2016. https://doi.org/10.1007/s13277-016-5135-6.
3. Wang X, Yang B, Ma B. The UCA1/miR-204/Sirt1 axis modulates docetaxel sensitivity of prostate cancer cells. Cancer Chemother Pharmacol. 2016; 78:1025–31. https://doi.org/10.1007/s00280-016-3158-8.
4. Lu Y, Li T, Wei G, Liu L, Chen Q, Xu L, Zhang K, Zeng D, Liao R. The long non-coding RNA NEAT1 regulates epithelial to mesenchymal transition and radioresistance in through miR-204/ZEB1 axis in nasopharyngeal carcinoma. Tumour Biol. 2016; 37:11733–41. https://doi.org/10.1007/s13277-015-4773-4.
5. Flores-Perez A, Marchat LA, Rodriguez-Cuevas S, Bautista-Pina V, Hidalgo-Miranda A, Ocampo EA, Martinez MS, Palma-Flores C, Fonseca-Sanchez MA, Astudillo-de la Vega H, Ruiz-Garcia E, Gonzalez-Barrios JA, Perez-Plasencia C, et al. Dual targeting of ANGPT1 and TGFBR2 genes by miR-204 controls angiogenesis in breast cancer. Sci Rep. 2016; 6:34504. https://doi.org/10.1038/srep34504.
6. Chattopadhyay E, Singh R, Ray A, Roy R, De Sarkar N, Paul RR, Pal M, Aich R, Roy B. Expression deregulation of mir31 and CXCL12 in two types of oral precancers and cancer: importance in progression of precancer and cancer. Sci Rep. 2016; 6:32735. https://doi.org/10.1038/srep32735.
7. Song S, Fajol A, Tu X, Ren B, Shi S. miR-204 suppresses the development and progression of human glioblastoma by targeting ATF2. Oncotarget. 2016. https://doi.org/10.18632/oncotarget.11732.
8. Wang X, Li F, Zhou X. miR-204-5p regulates cell proliferation and metastasis through inhibiting CXCR4 expression in OSCC. Biomed Pharmacother. 2016; 82:202–7. https://doi.org/10.1016/j.biopha.2016.04.060.
9. Toll A, Salgado R, Espinet B, Diaz-Lagares A, Hernandez-Ruiz E, Andrades E, Sandoval J, Esteller M, Pujol RM, Hernandez-Munoz I. MiR-204 silencing in intraepithelial to invasive cutaneous squamous cell carcinoma progression. Mol Cancer. 2016; 15:53. https://doi.org/10.1186/s12943-016-0537-z.
10. Zhang S, Gao L, Thakur A, Shi P, Liu F, Feng J, Wang T, Liang Y, Liu JJ, Chen M, Ren H. miRNA-204 suppresses human non-small cell lung cancer by targeting ATF2. Tumour Biol. 2016; 37:11177–86. https://doi.org/10.1007/s13277-016-4906-4.
11. Xia Z, Liu F, Zhang J, Liu L. Decreased Expression of MiRNA-204-5p Contributes to Glioma Progression and Promotes Glioma Cell Growth, Migration and Invasion. PLoS One. 2015; 10:e0132399. https://doi.org/10.1371/journal.pone.0132399.
12. Zhang B, Yin Y, Hu Y, Zhang J, Bian Z, Song M, Hua D, Huang Z. MicroRNA-204-5p inhibits gastric cancer cell proliferation by downregulating USP47 and RAB22A. Med Oncol. 2015; 32:331. https://doi.org/10.1007/s12032-014-0331-y.
13. Wang X, Qiu W, Zhang G, Xu S, Gao Q, Yang Z. MicroRNA-204 targets JAK2 in breast cancer and induces cell apoptosis through the STAT3/BCl-2/survivin pathway. Int J Clin Exp Pathol. 2015; 8:5017–25.
14. Wu ZY, Wang SM, Chen ZH, Huv SX, Huang K, Huang BJ, Du JL, Huang CM, Peng L, Jian ZX, Zhao G. MiR-204 regulates HMGA2 expression and inhibits cell proliferation in human thyroid cancer. Cancer Biomark. 2015; 15:535–42. https://doi.org/10.3233/CBM-150492.
15. Bachetti T, Di Zanni E, Ravazzolo R, Ceccherini I. miR-204 mediates post-transcriptional down-regulation of PHOX2B gene expression in neuroblastoma cells. Biochim Biophys Acta. 2015; 1849:1057–65. https://doi.org/10.1016/j.bbagrm.2015.06.008.
16. Mao J, Zhang M, Zhong M, Zhang Y, Lv K. MicroRNA-204, a direct negative regulator of ezrin gene expression, inhibits glioma cell migration and invasion. Mol Cell Biochem. 2014; 396:117–28. https://doi.org/10.1007/s11010-014-2148-6.

17. Chung TK, Lau TS, Cheung TH, Yim SF, Lo KW, Siu NS, Chan LK, Yu MY, Kwong J, Doran G, Barroilhet LM, Ng AS, Wong RR, et al. Dysregulation of microRNA-204 mediates migration and invasion of endometrial cancer by regulating FOXC1. Int J Cancer. 2012; 130:1036–45. https://doi.org/10.1002/ijc.26060.
18. Xiong F, Liu K, Zhang F, Sha K, Wang X, Guo X, Huang N. MiR-204 inhibits the proliferation and invasion of renal cell carcinoma by inhibiting RAB22A expression. Oncol Rep. 2016; 35:3000–8. https://doi.org/10.3892/or.2016.4624.
19. Todorova K, Metodiev MV, Metodieva G, Zasheva D, Mincheff M, Hayrabedyan S. miR-204 is dysregulated in metastatic prostate cancer in vitro. Mol Carcinog. 2016; 55:131–47. https://doi.org/10.1002/mc.22263.
20. Chen Z, Sangwan V, Banerjee S, Mackenzie T, Dudeja V, Li X, Wang H, Vickers SM, Saluja AK. miR-204 mediated loss of Myeloid cell leukemia-1 results in pancreatic cancer cell death. Mol Cancer. 2013; 12:105. https://doi.org/10.1186/1476-4598-12-105.
21. Jiang G, Wen L, Zheng H, Jian Z, Deng W. miR-204-5p targeting SIRT1 regulates hepatocellular carcinoma progression. Cell Biochem Funct. 2016; 34:505–10. https://doi.org/10.1002/cbf.3223.
22. Huang JY, Chen HL, Shih C. MicroRNA miR-204 and miR-1236 inhibit hepatitis B virus replication via two different mechanisms. Sci Rep. 2016; 6:34740. https://doi.org/10.1038/srep34740.
23. Lin YC, Lin JF, Tsai TF, Chou KY, Chen HE, Hwang TI. Tumor suppressor miRNA-204-5p promotes apoptosis by targeting BCL2 in prostate cancer cells. Asian J Surg. 2016. https://doi.org/10.1016/j.asjsur.2016.07.001.
24. Liu L, Wang J, Li X, Ma J, Shi C, Zhu H, Xi Q, Zhang J, Zhao X, Gu M. MiR-204-5p suppresses cell proliferation by inhibiting IGFBP5 in papillary thyroid carcinoma. Biochem Biophys Res Commun. 2015; 457:621–6. https://doi.org/10.1016/j.bbrc.2015.01.037.
25. Wu D, Pan H, Zhou Y, Zhang Z, Qu P, Zhou J, Wang W. Upregulation of microRNA-204 inhibits cell proliferation, migration and invasion in human renal cell carcinoma cells by downregulating SOX4. Mol Med Rep. 2015; 12:7059–64. https://doi.org/10.3892/mmr.2015.4259.
26. Sun Y, Yu X, Bai Q. miR-204 inhibits invasion and epithelial-mesenchymal transition by targeting FOXM1 in esophageal cancer. Int J Clin Exp Pathol. 2015; 8:12775–83.
27. Gong M, Ma J, Li M, Zhou M, Hock JM, Yu X. MicroRNA-204 critically regulates carcinogenesis in malignant peripheral nerve sheath tumors. Neuro Oncol. 2012; 14:1007–17. https://doi.org/10.1093/neuonc/nos124.
28. Wang P, Lv HY, Zhou DM, Zhang EN. miR-204 suppresses non-small-cell lung carcinoma (NSCLC) invasion and migration by targeting JAK2. Genet Mol Res. 2016; 15. https://doi.org/10.4238/gmr.15026415.
29. Hartz JM, Engelmann D, Furst K, Marquardt S, Spitschak A, Goody D, Protzel C, Hakenberg OW, Putzer BM. Integrated Loss of miR-1/miR-101/miR-204 Discriminates Metastatic from Nonmetastatic Penile Carcinomas and Can Predict Patient Outcome. J Urol. 2016; 196:570–8. https://doi.org/10.1016/j.juro.2016.01.115.
30. Li T, Pan H, Li R. The dual regulatory role of miR-204 in cancer. Tumour Biol. 2016; 37:11667–77. https://doi.org/10.1007/s13277-016-5144-5.

Supplementary Data 4: The process of search, data extraction and combination.
See Supplementary_Data_4

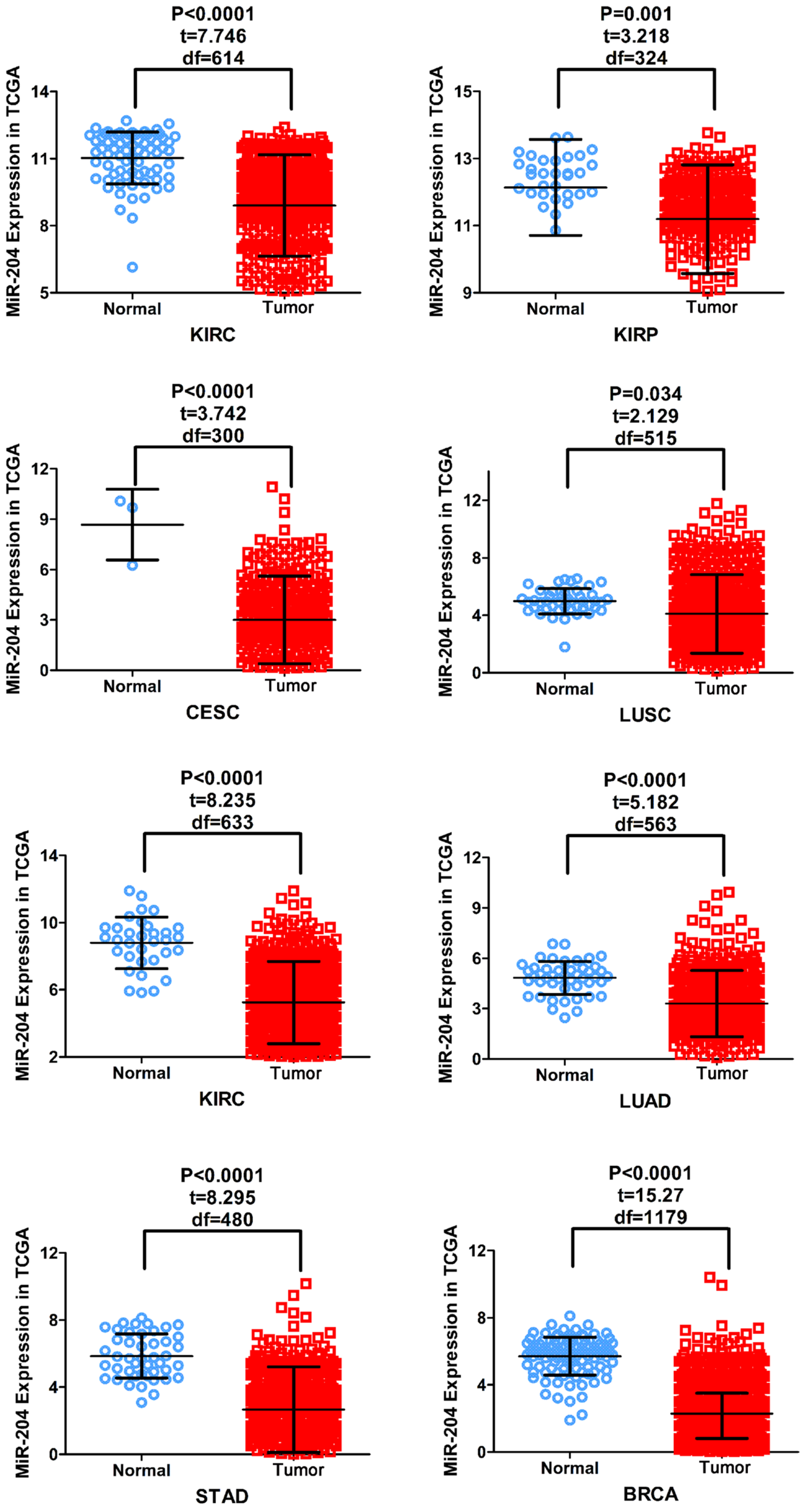

Supplementary Figure 1: The expression of miR-204-5p in cancers in TCGA. Down-regulation of miR-204-5p was detected in KIRC, KIRP, CESC, LUSC, UCEC, LUAD, STAD and BRCA compared with corresponding non-cancerous tissues. KIRC (kidney renal clear cell carcinoma); KIRP (kidney renal papillary cell carcinoma); CESC (cervical squamous cell carcinoma and endocervical adenocarcinoma); LUSC (lung squamous cell carcinoma); UCEC (uterine corpus endometrial carcinoma); LUAD (lung adenocarcinoma); STAD (stomach adenocarcinoma); BRCA (breast invasive carcinoma).

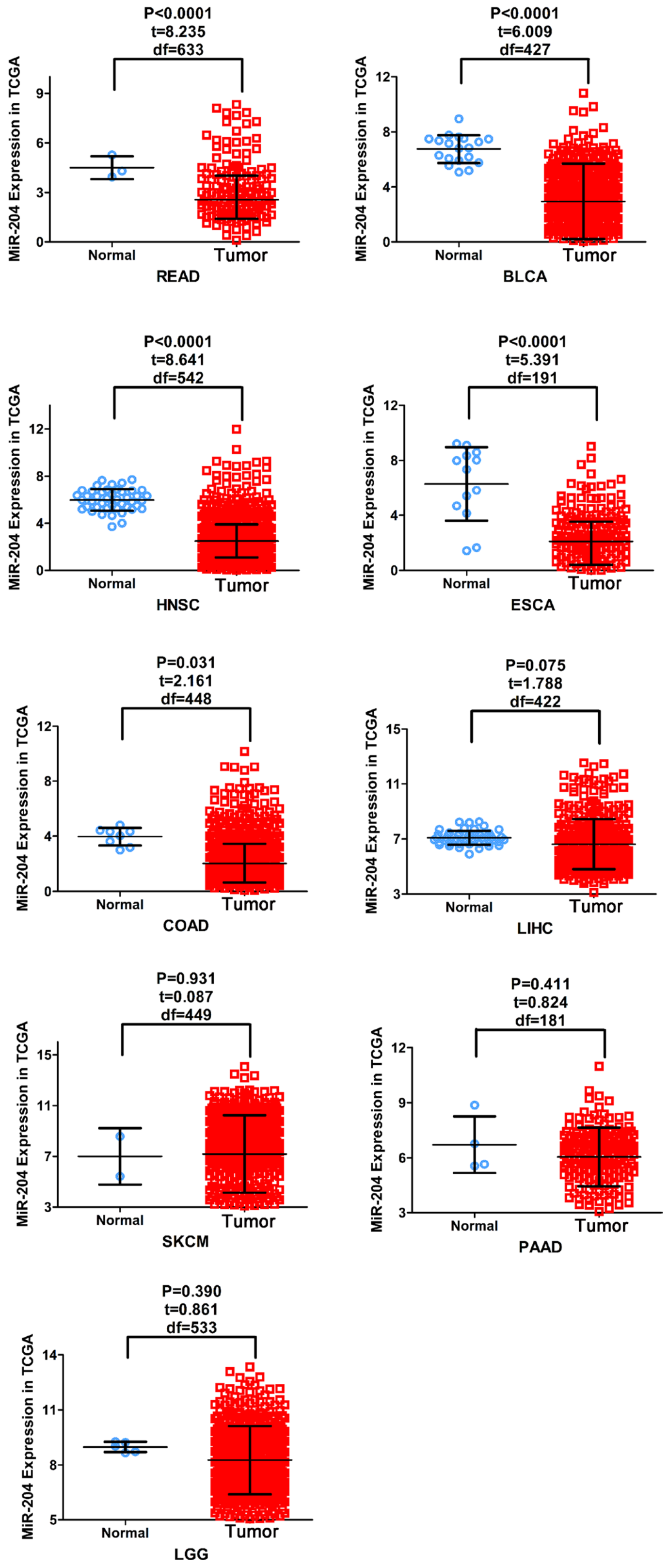

Supplementary Figure 2: The expression of miR-204-5p in cancers in TCGA. Down-regulation of miR-204-5p was detected in READ, BLCA, HNSC, ESCA and COAD compared with corresponding non-cancerous tissues. READ (rectum adenocarcinoma); BLCA (bladder urothelial carcinoma); HNSC (head and neck squamous cell carcinoma); ESCA (esophageal carcinoma); COAD (colon adenocarcinoma).

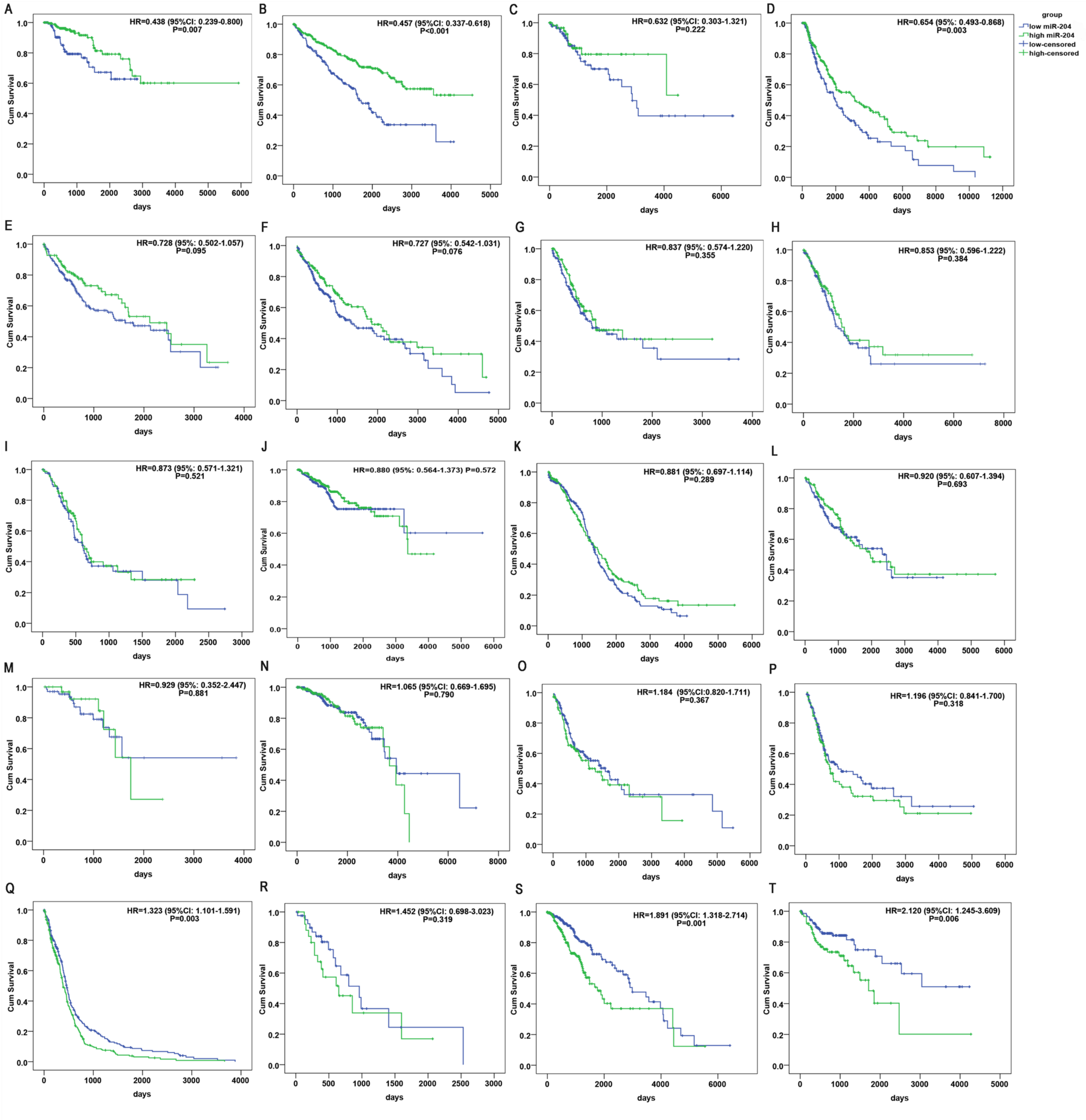

Supplementary Figure 3: The survival curves of miR-204-5p in 20 cancers in TCGA. The survival curves were arranged by the HR value. HR: hazard ratio; CI: confidence interval. (**A**) KIRP (kidney renal papillary cell carcinoma); (**B**) KIRC (kidney renal clear cell carcinoma); (**C**) CESC (cervical squamous cell carcinama and endocervical adenocarcinoma); (**D**) SKCM (skin cutaneous melanoma); (**E**) LIHC (liver hepatocellular carcinoma); (**F**) LUSC (lung squamous cell carcinoma); (**G**) STAD (stomach adenocarcinoma); (**H**) LUAD (lung adenocarcinoma); (**I**) PAAD (pancreatic adenocarcinoma); (**J**) UCEC (uterine corpus endometrial carcinoma); (**K**) OV (ovarian serous cystadenocarcinoma); (**L**) SARC (sarcoma); (**M**) READ (rectum adenocarcinoma); (**N**) BRCA (breast invasive carcinoma); (**O**) HNSC (head and neck squamous cell carcinoma); (**P**) BLCA (bladder urothelial carcinoma); (**Q**) GBM (glioblastoma multiforme); (**R**) ESCA (esophageal carcinoma); (**S**) LGG (brain lower grade glioma); (**T**) COAD (colon adenocarcinoma).

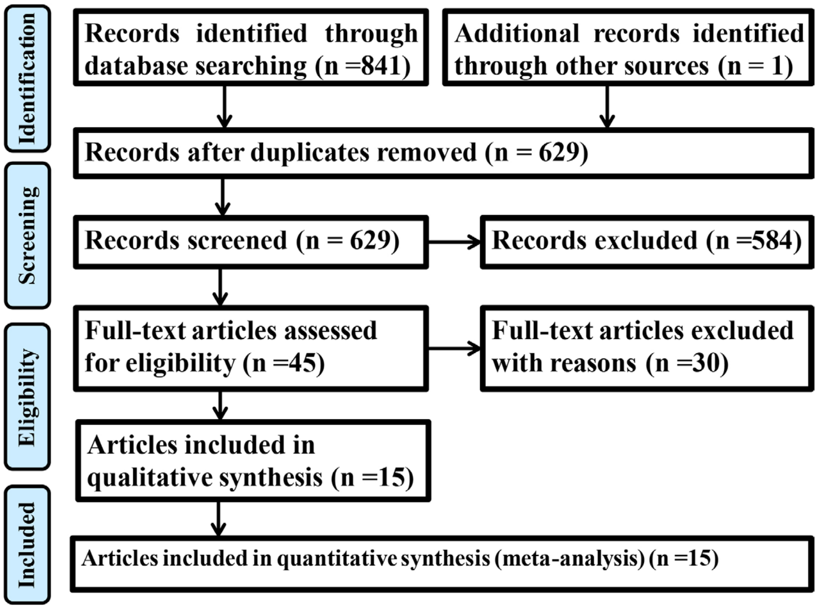

Supplementary Figure 4: The flow chart of meta-analysis for literature search.

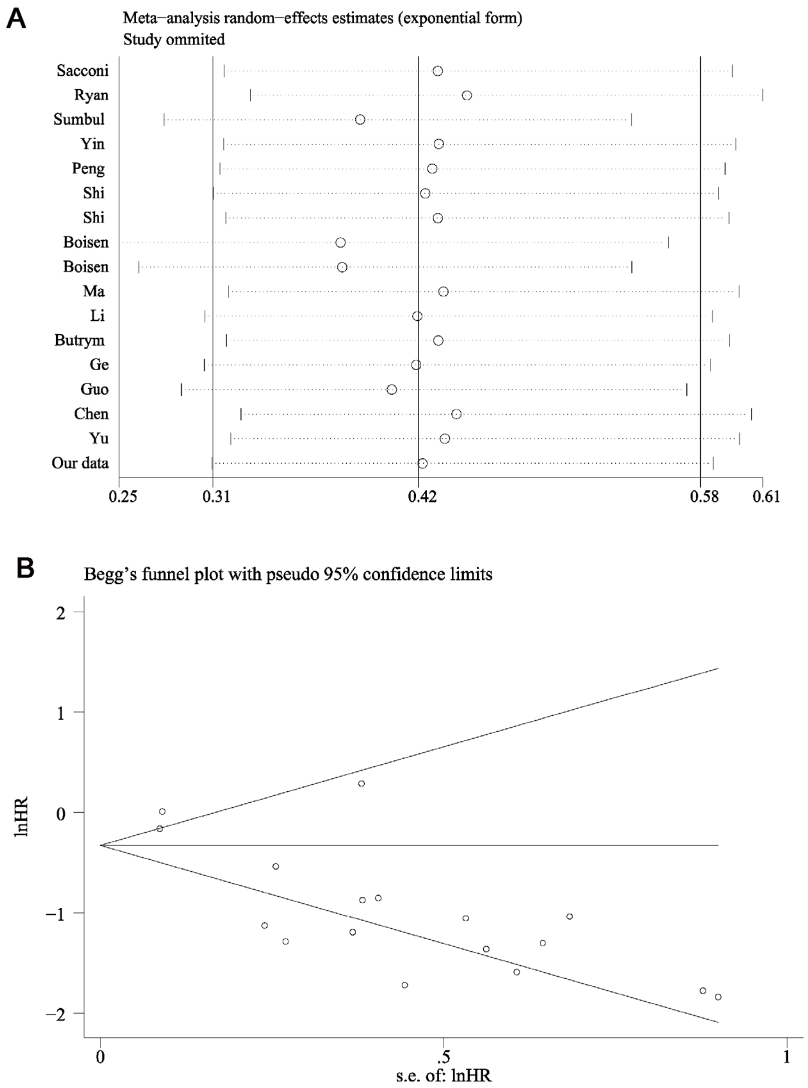

Supplementary Figure 5: The sensitivity analysis of included studies and funnel plots for publication bias. (**A**) Sensitivity analysis; (**B**) Funnel plots of 15 included studies (17 cohorts, 1783 cases), Begg’s test: $P = 0.434$.
