# Supplementary material for: The protective value of miR-204-5p for prognosis and its potential gene network in various malignancies: a comprehensive exploration based on RNA-seq high-throughput data and bioinformatics
Source: Oncotarget. 2017 Oct 23;8(62):104960–80. doi: 10.18632/oncotarget.21950 (PMC5739612; doi:10.18632/oncotarget.21950)
Supplement: Supplementary file 3 [file oncotarget-08-104960-s003.pptx]

## Slide 1
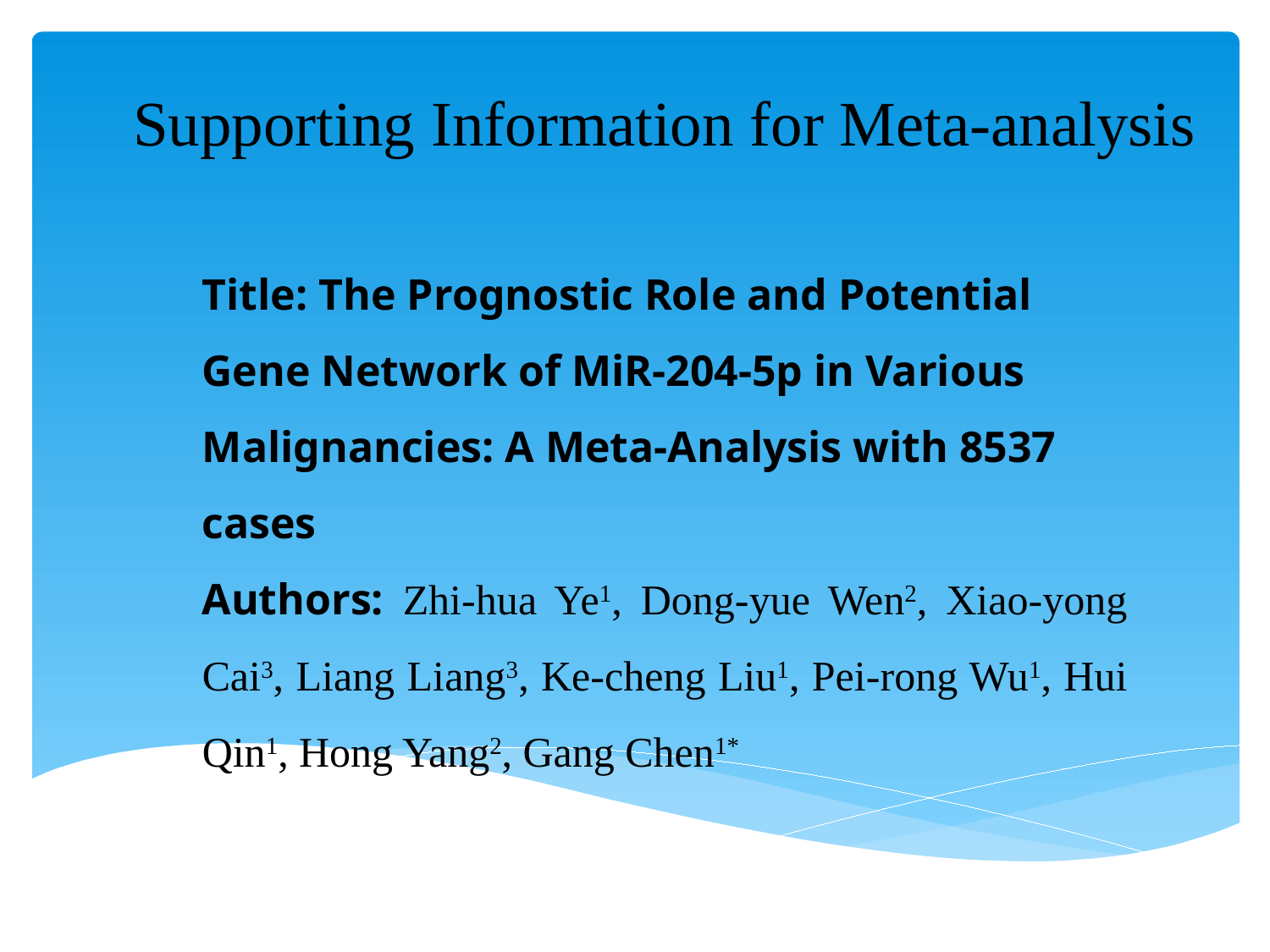

Supporting Information for Meta-analysis
Title: The Prognostic Role and Potential Gene Network of MiR-204-5p in Various Malignancies: A Meta-Analysis with 8537 cases
Authors: Zhi-hua Ye1, Dong-yue Wen2, Xiao-yong Cai3, Liang Liang3, Ke-cheng Liu1, Pei-rong Wu1, Hui Qin1, Hong Yang2, Gang Chen1*

## Slide 2
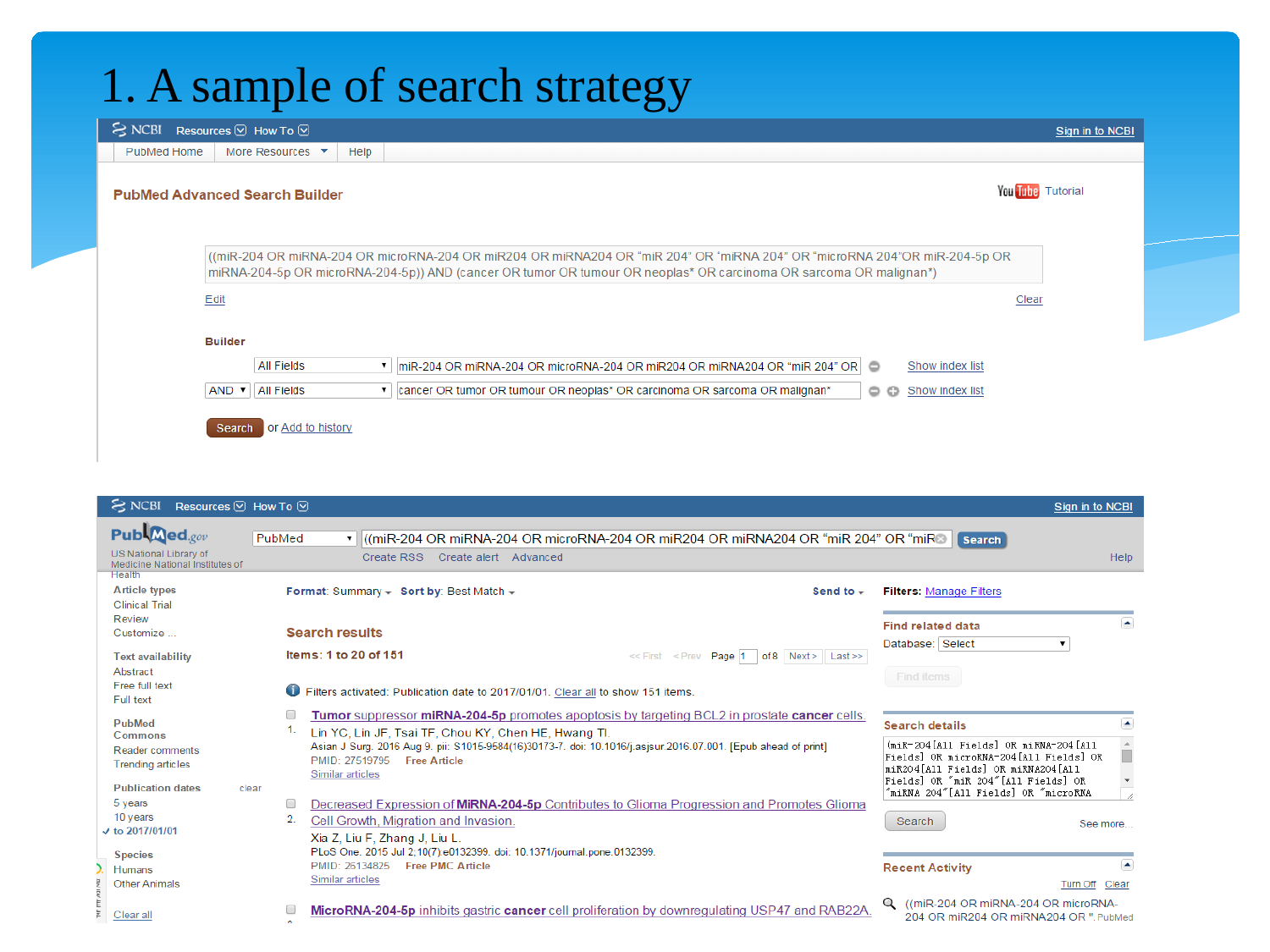

1. A sample of search strategy

## Slide 3
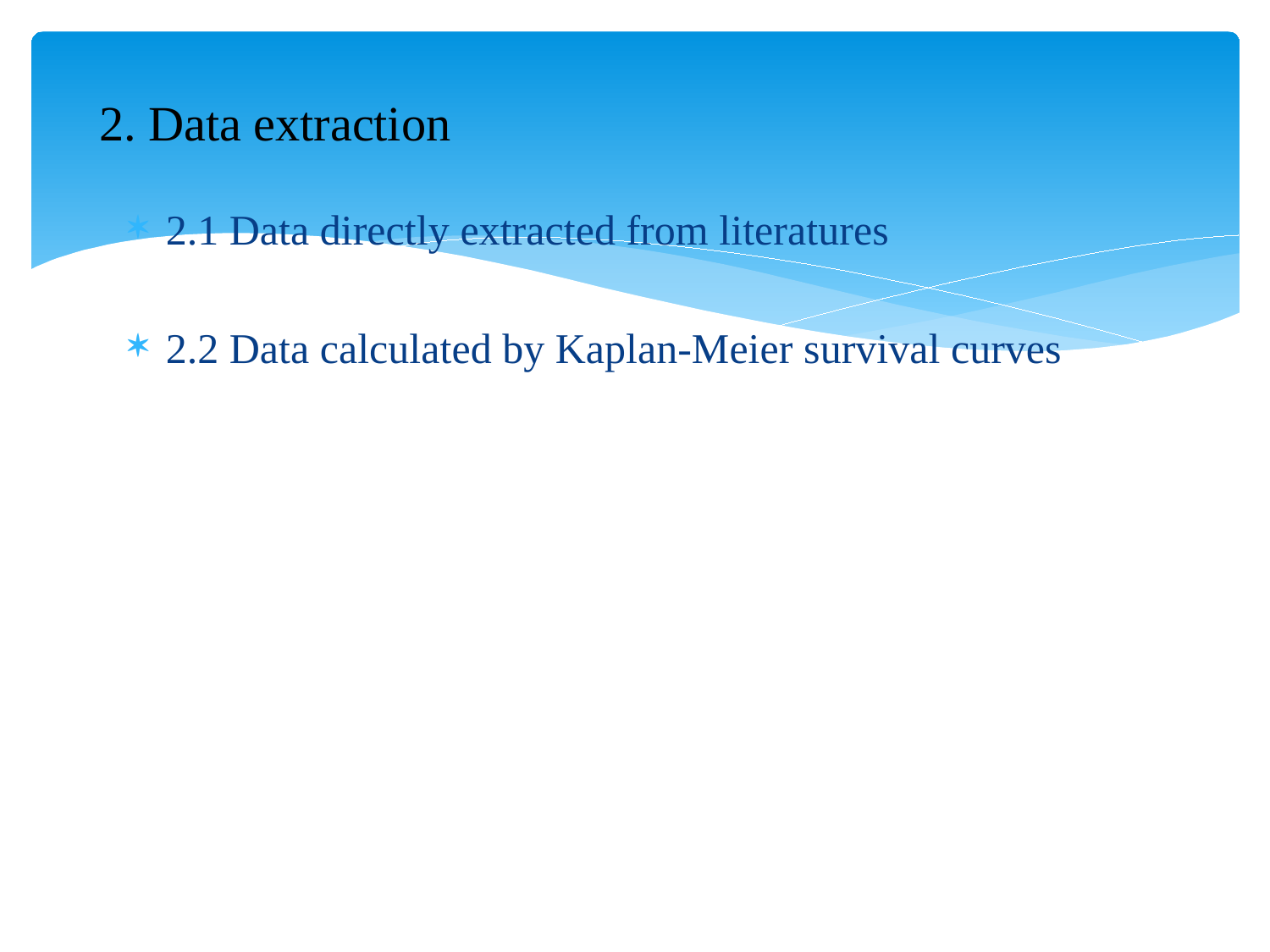

2. Data extraction
2.1 Data directly extracted from literatures
2.2 Data calculated by Kaplan-Meier survival curves

## Slide 4
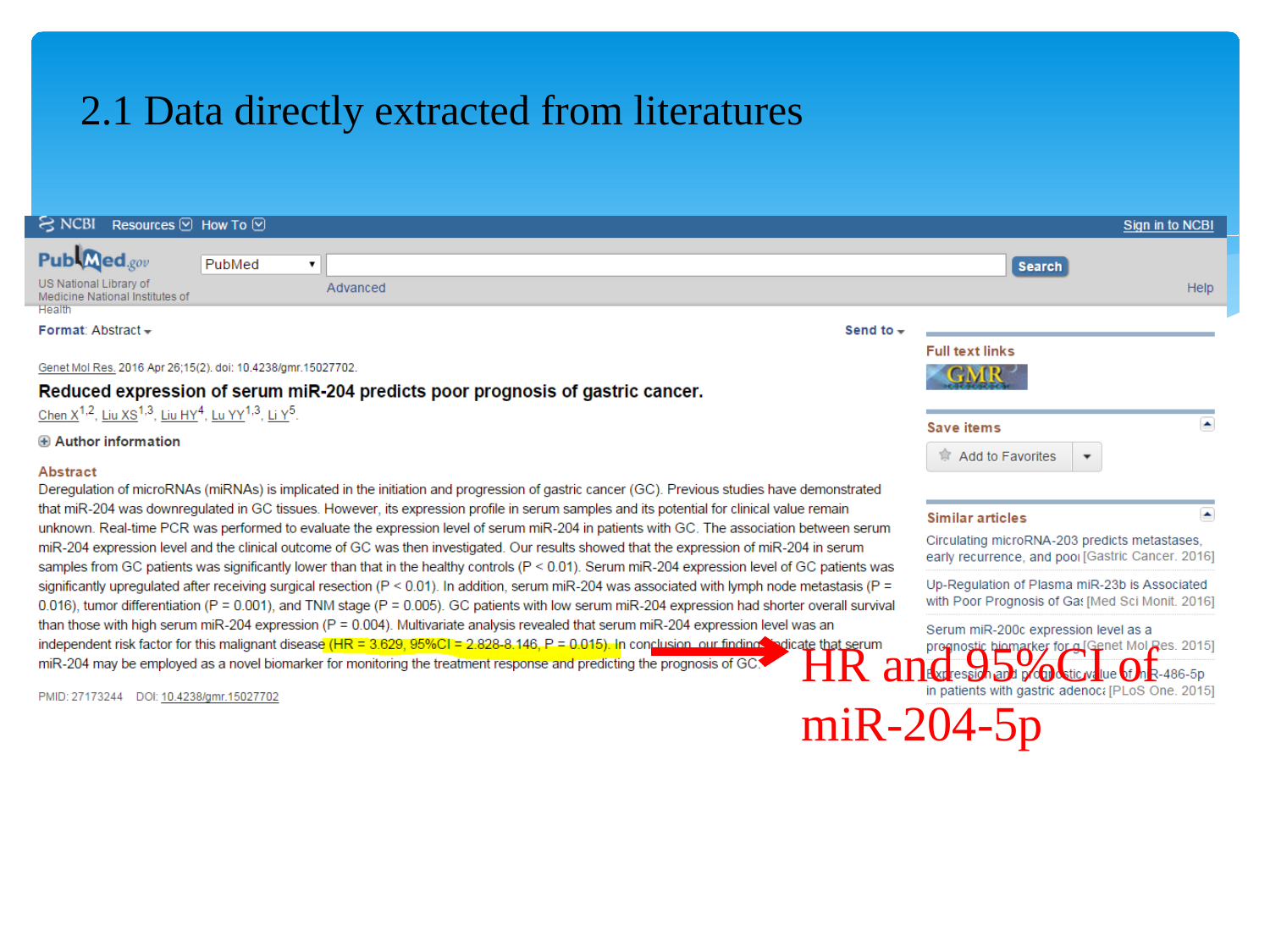

2.1 Data directly extracted from literatures
HR and 95%CI of miR-204-5p

## Slide 5
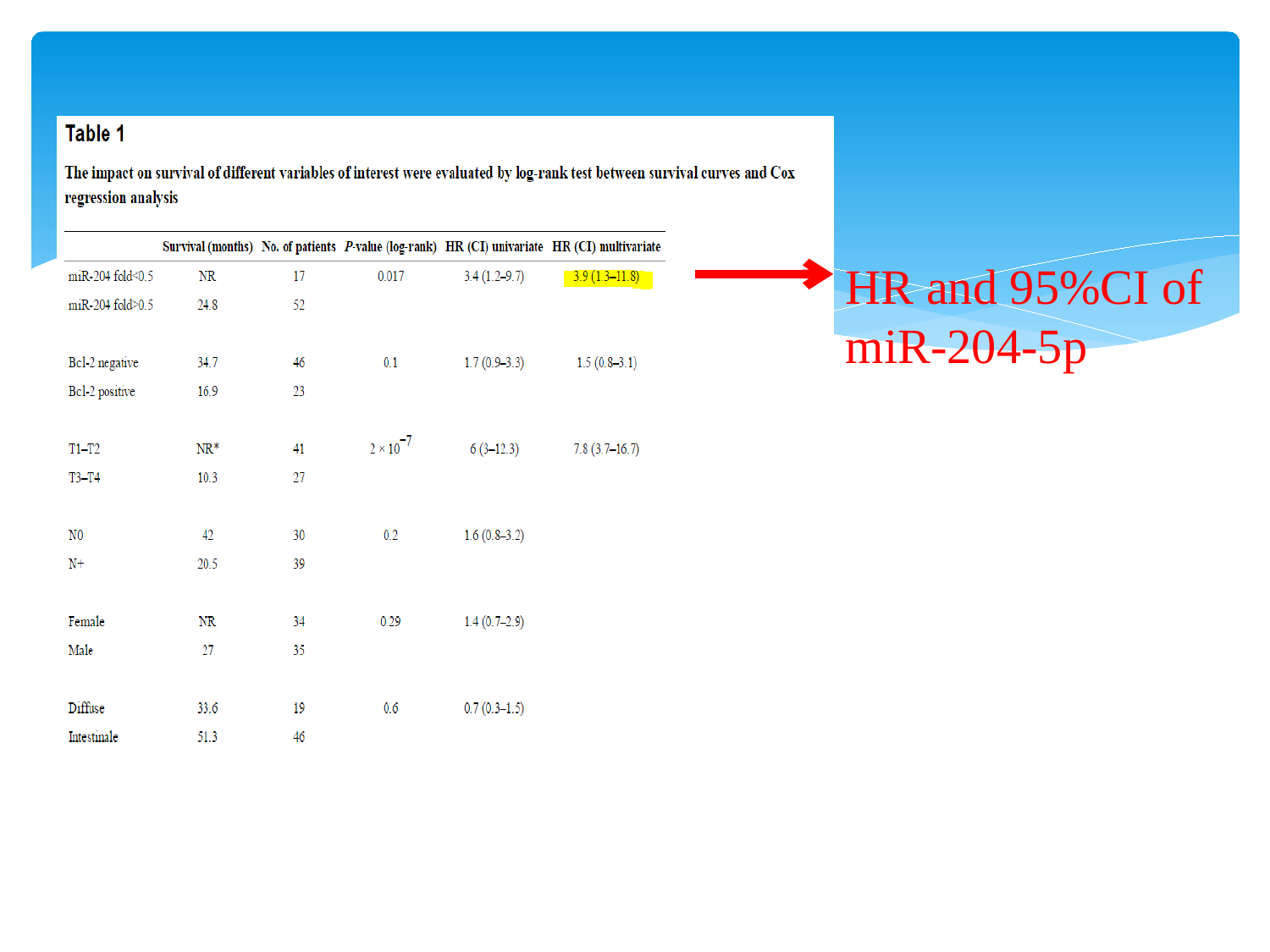

HR and 95%CI of miR-204-5p

## Slide 6
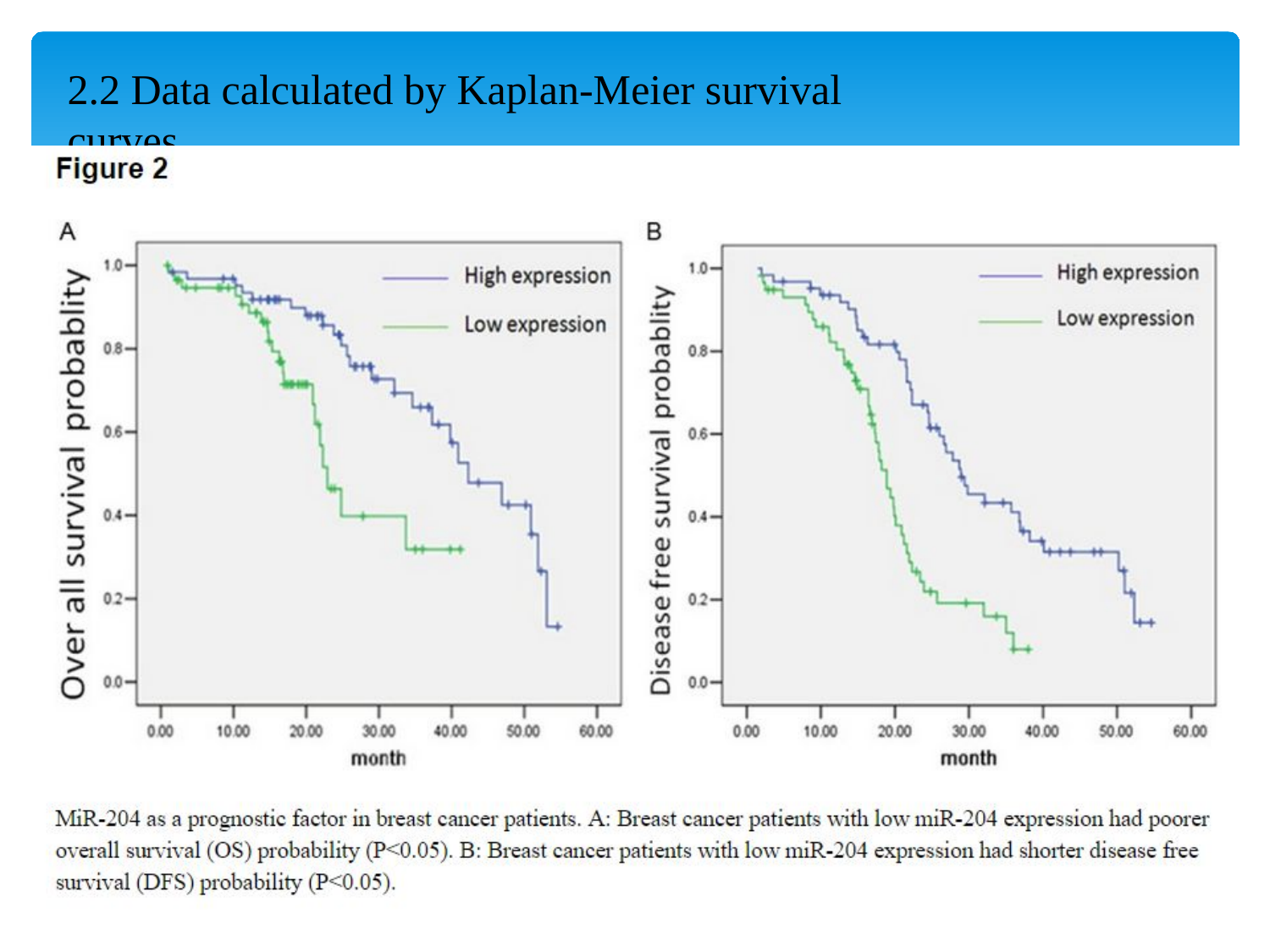

2.2 Data calculated by Kaplan-Meier survival curves
No 4. survival curve
 offered in the study

## Slide 7
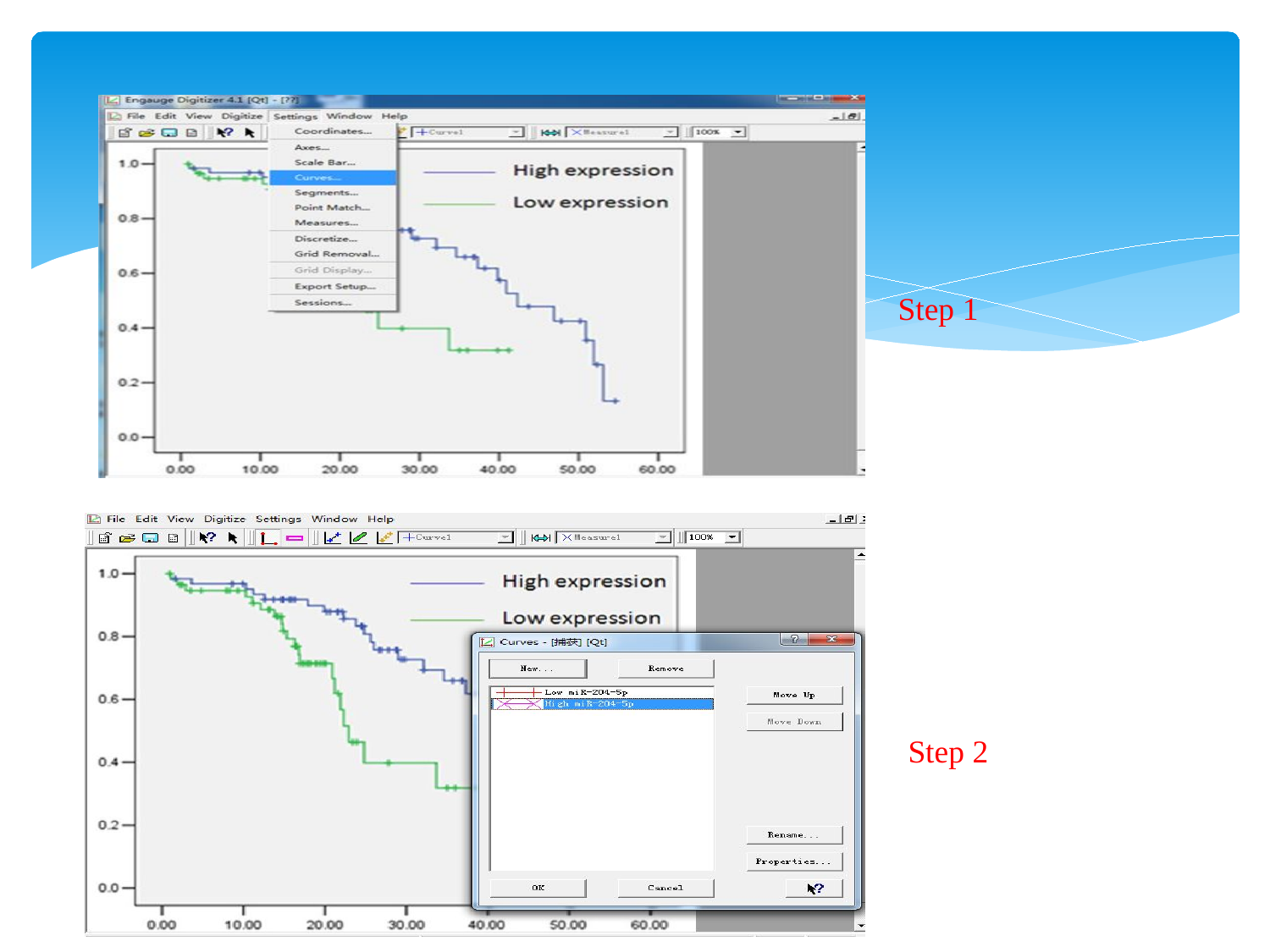

Step 1
Step 2

## Slide 8
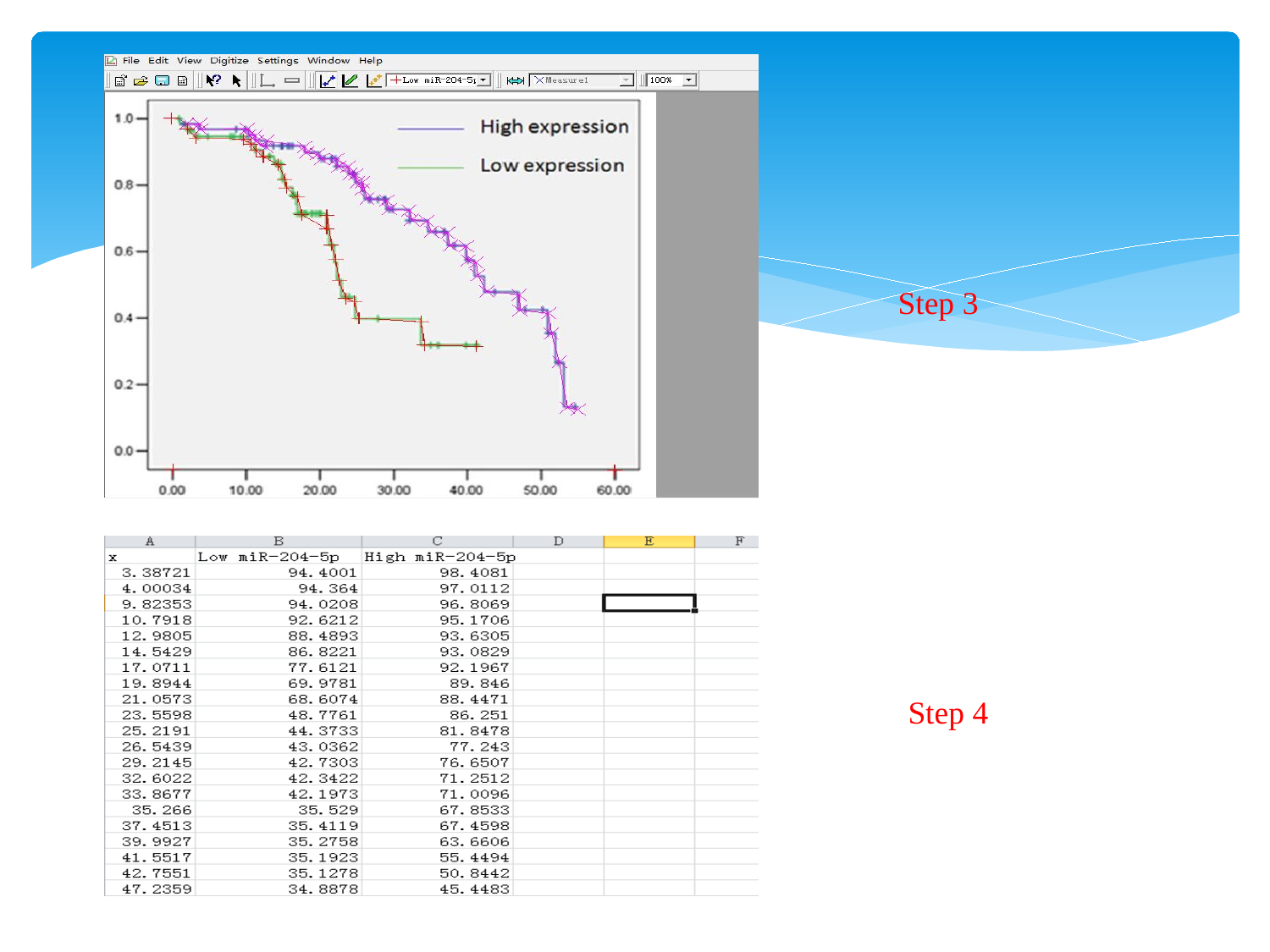

Step 3
Step 4

## Slide 9
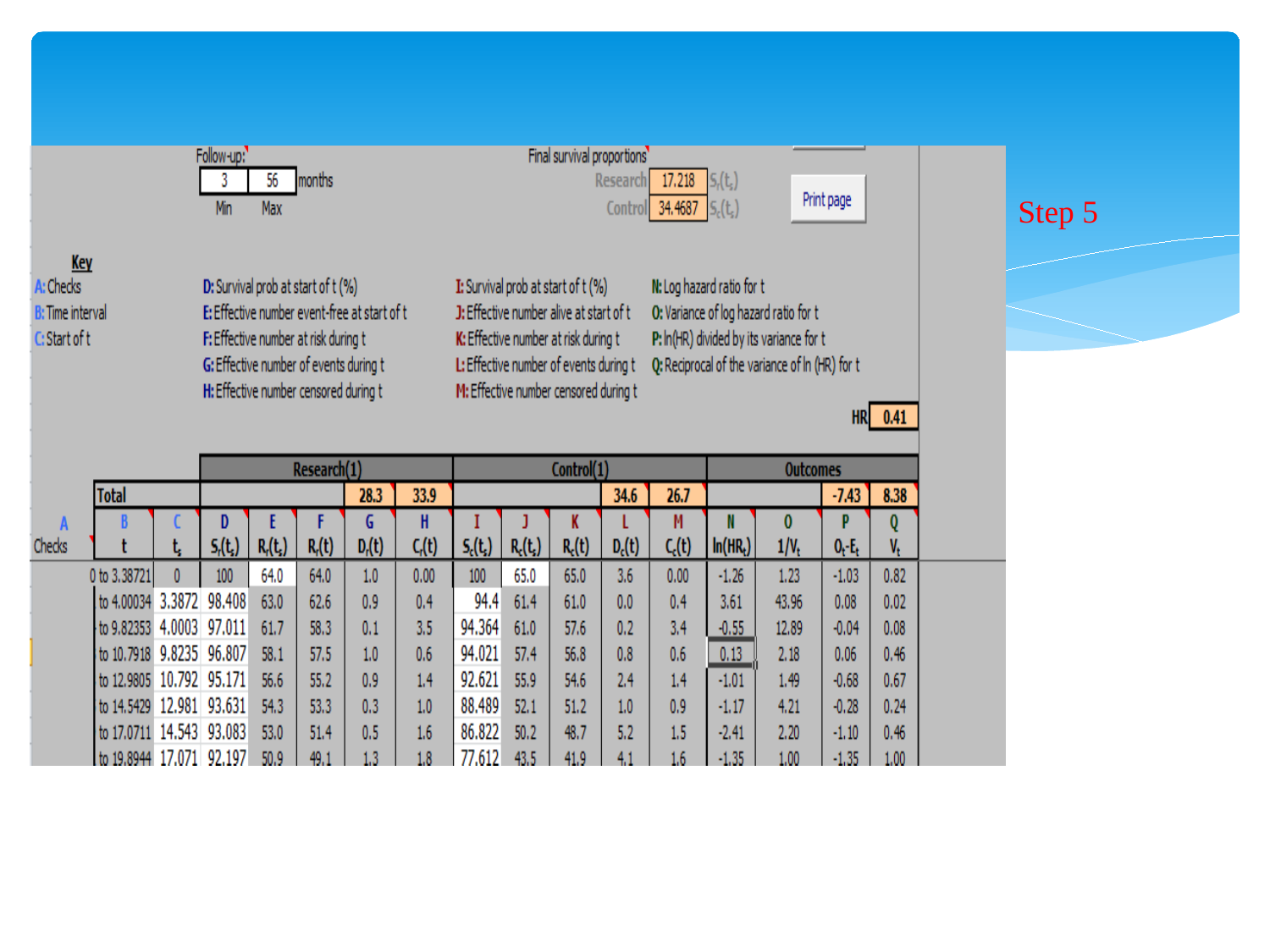

Step 5

## Slide 10
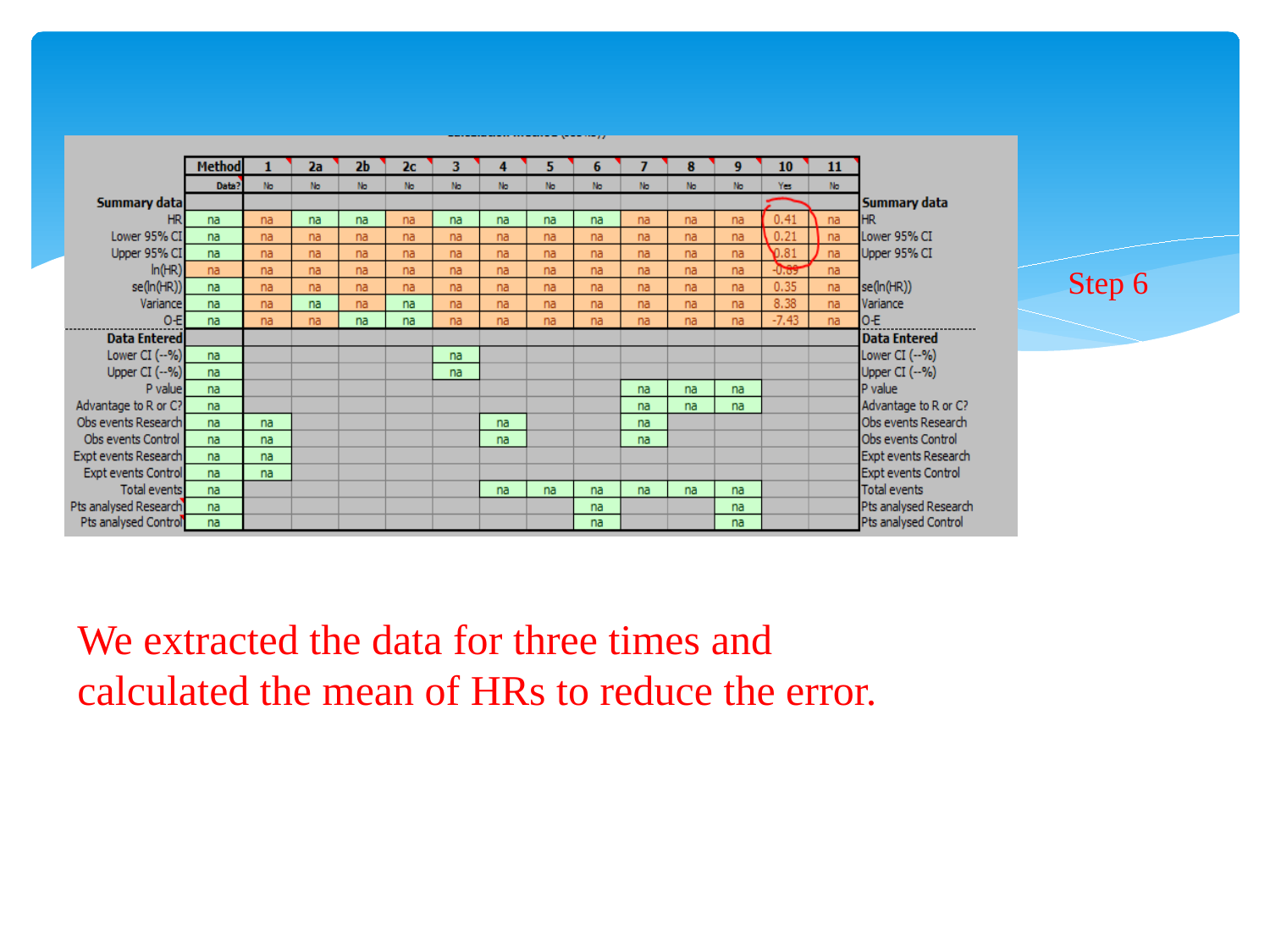

Step 6
We extracted the data for three times and calculated the mean of HRs to reduce the error.

## Slide 11
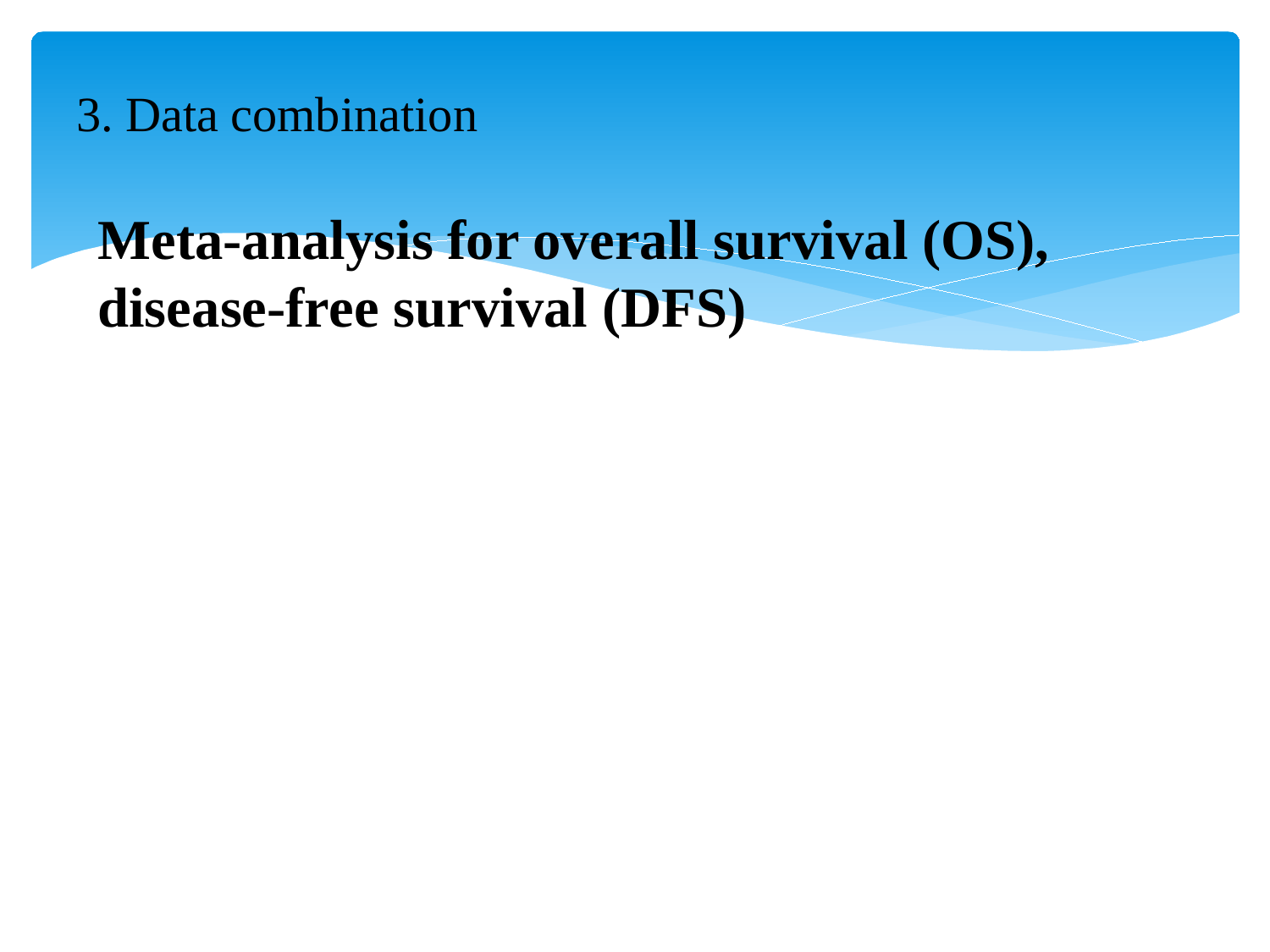

3. Data combination
Meta-analysis for overall survival (OS), disease-free survival (DFS)

## Slide 12
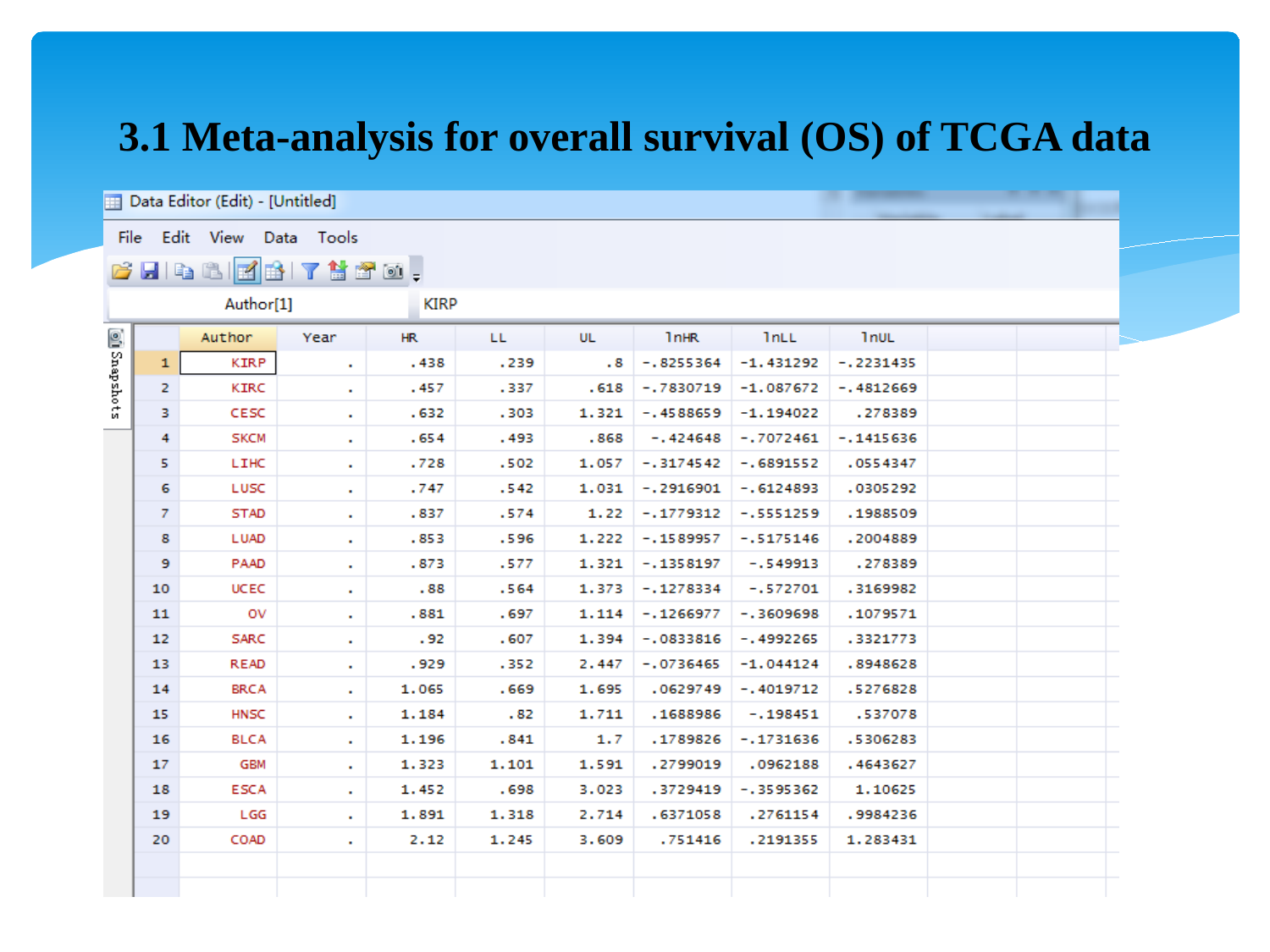

# 3.1 Meta-analysis for overall survival (OS) of TCGA data

## Slide 13
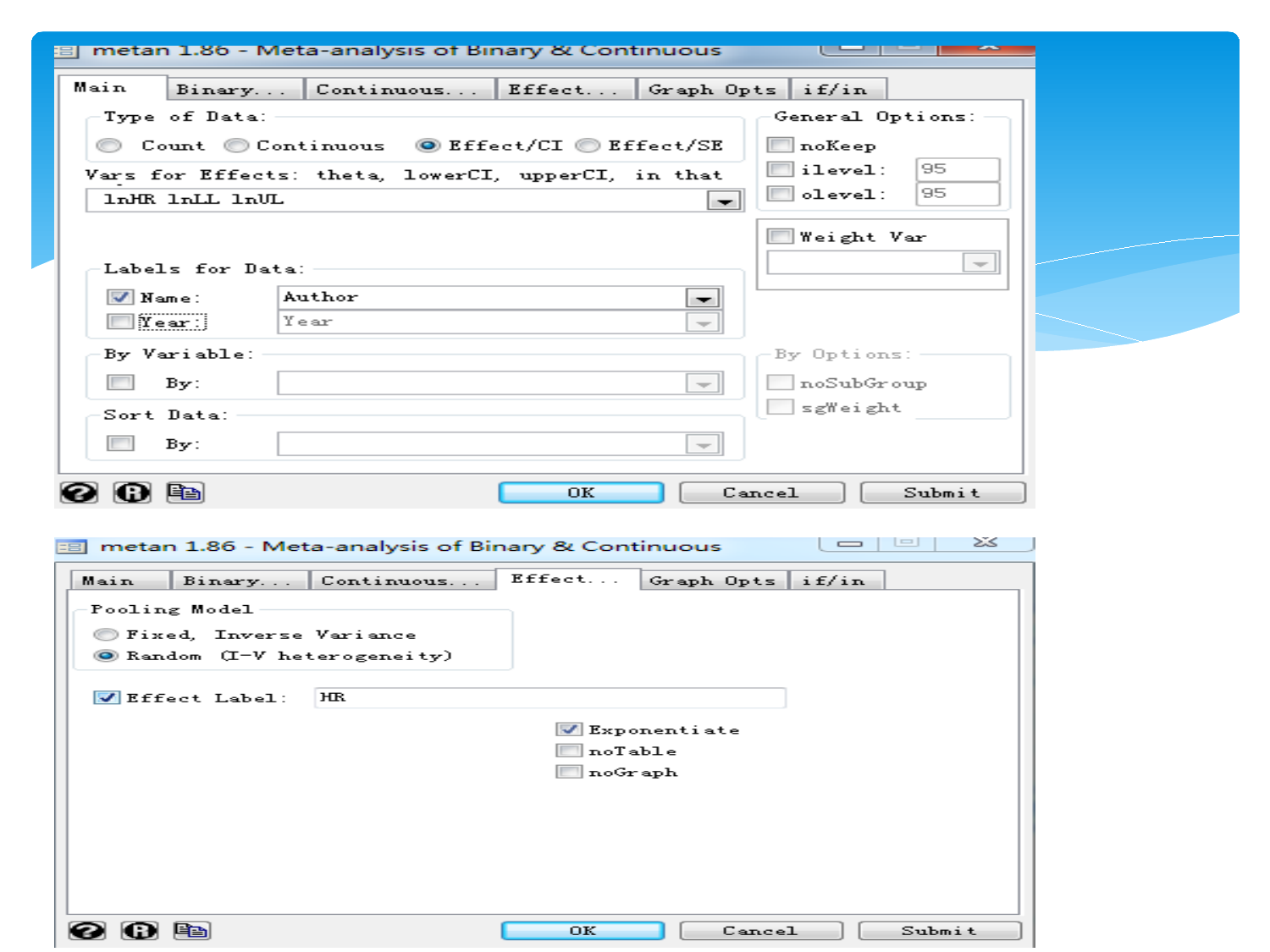

#

## Slide 14
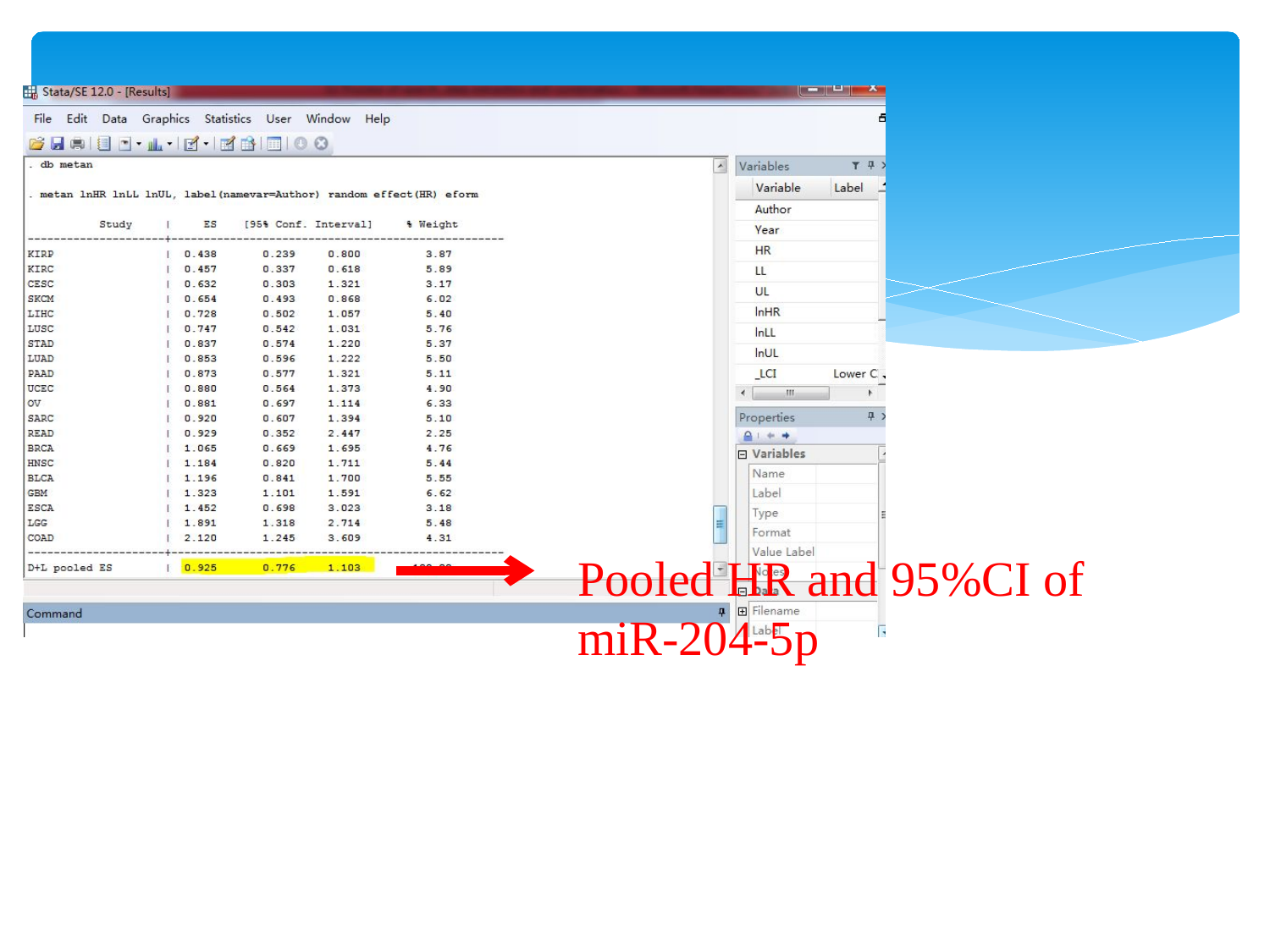

Pooled HR and 95%CI of miR-204-5p

## Slide 15
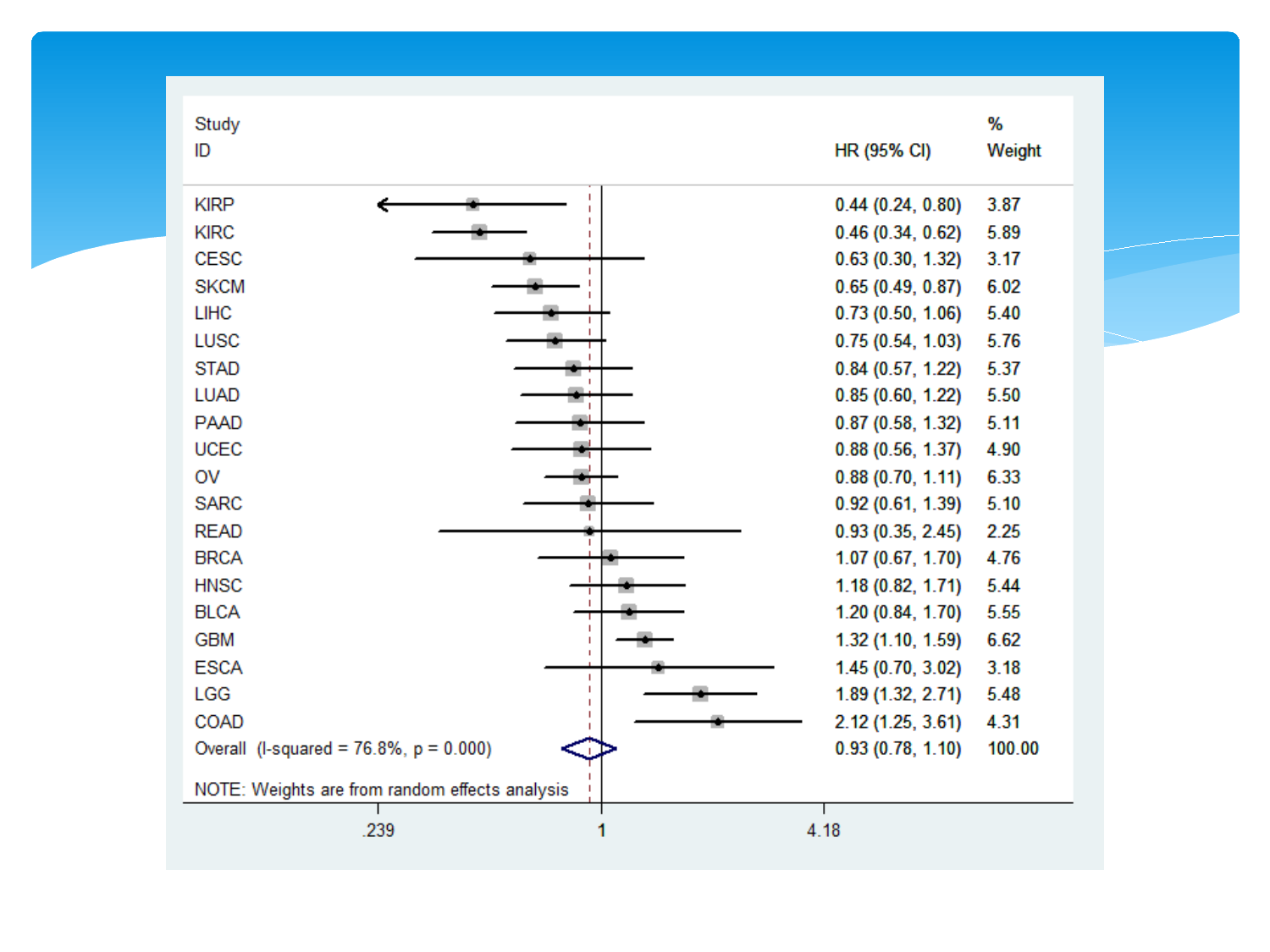

## Slide 16
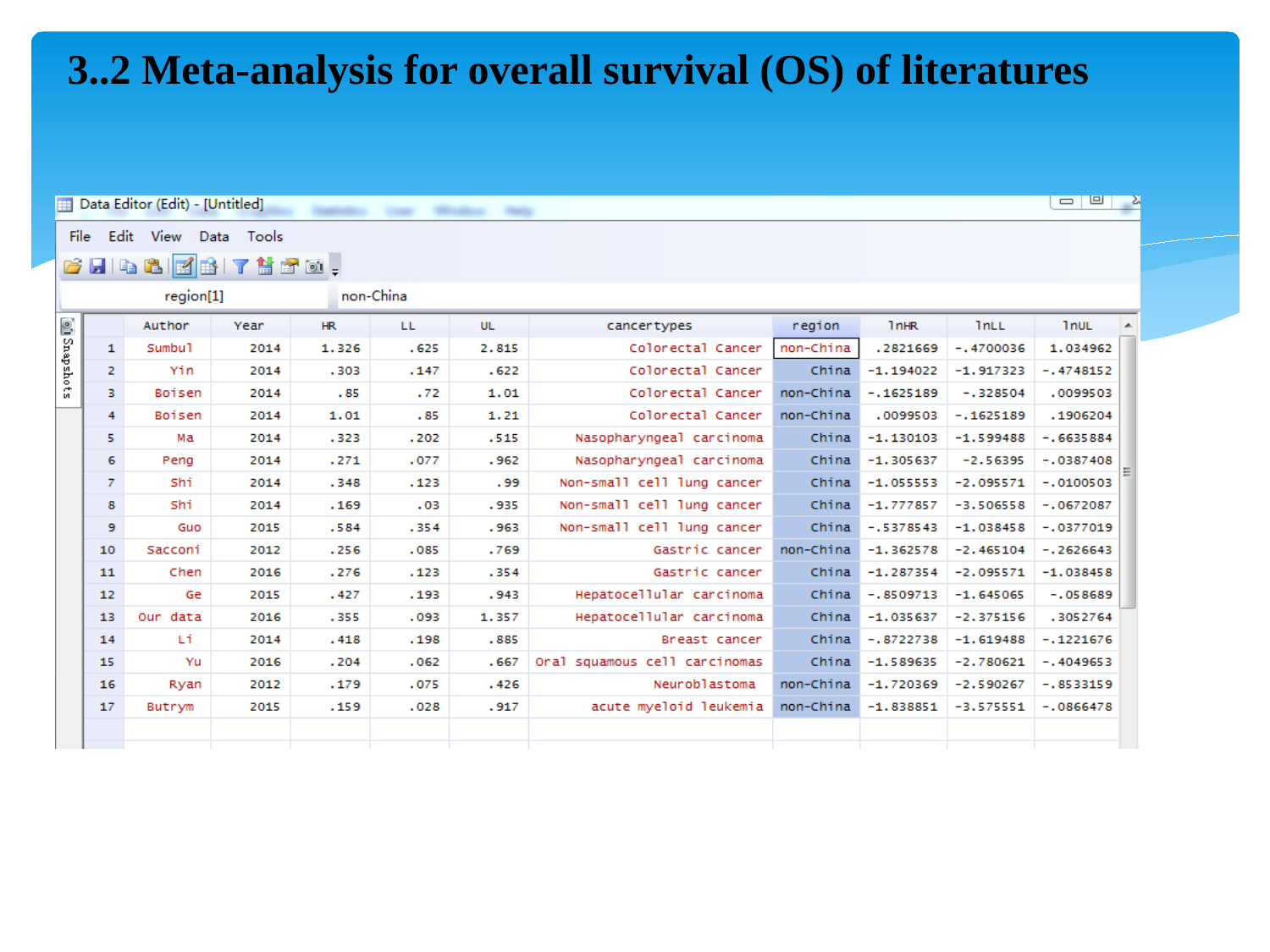

3..2 Meta-analysis for overall survival (OS) of literatures

## Slide 17
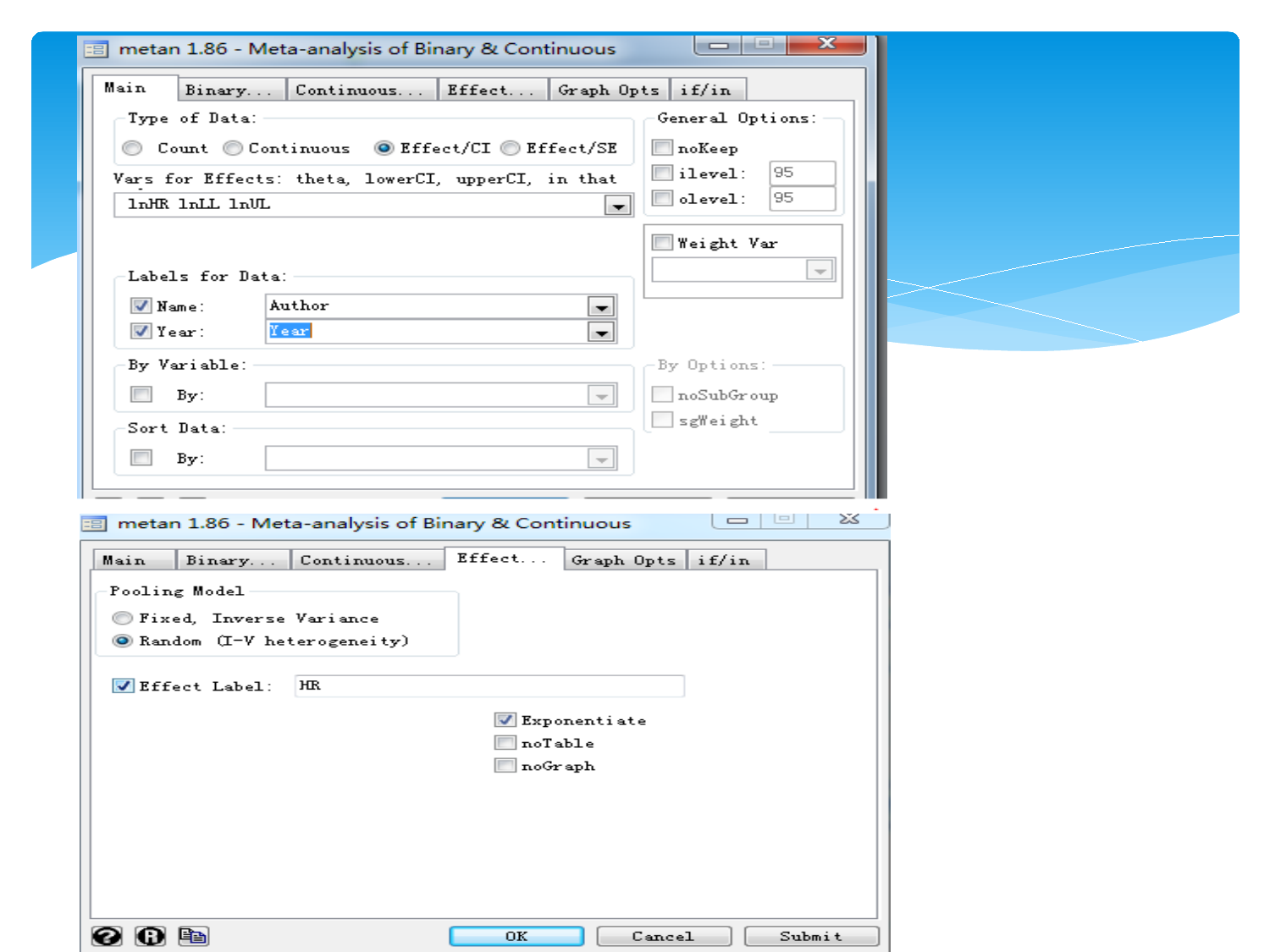

## Slide 18
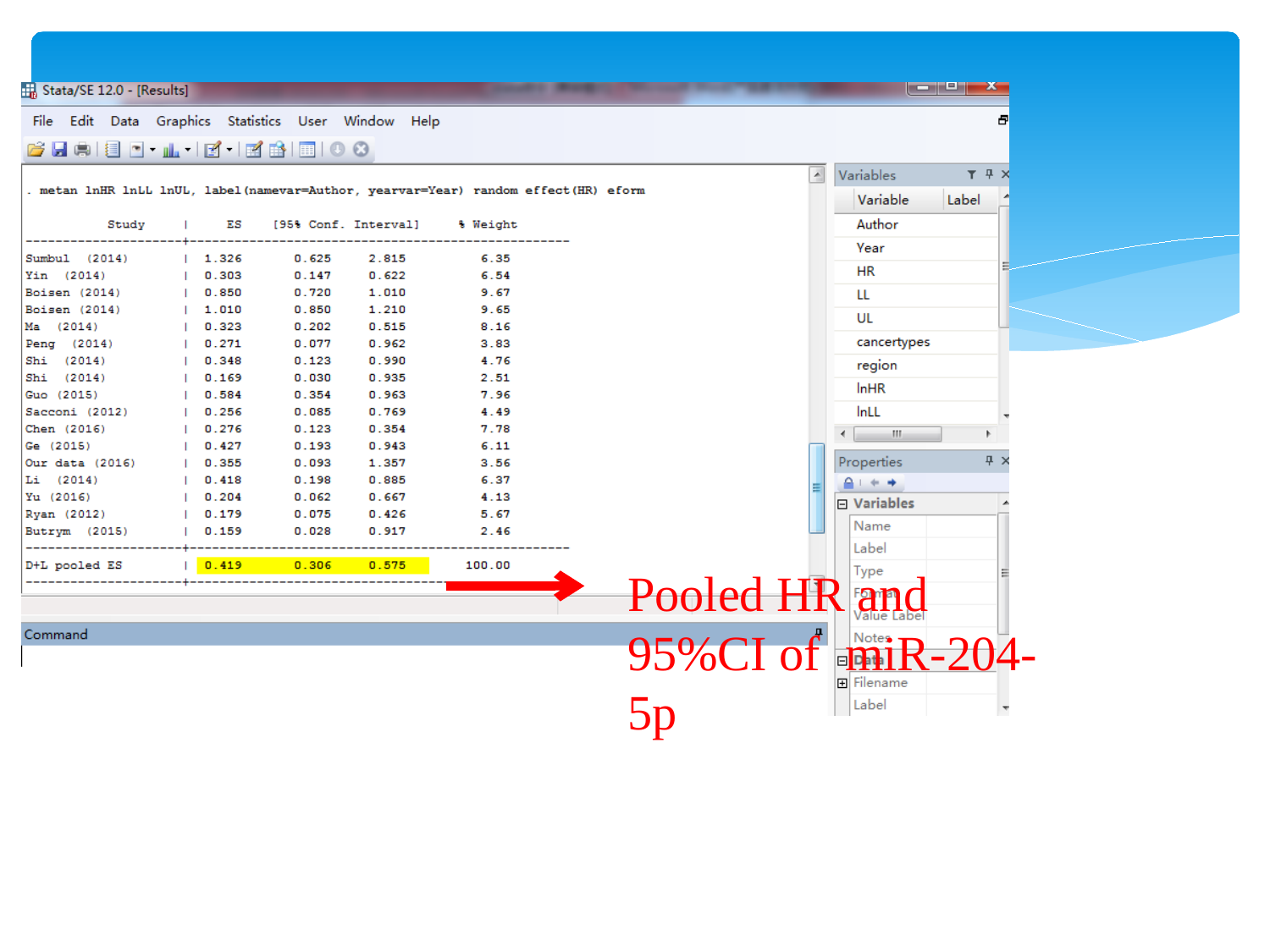

Pooled HR and 95%CI of miR-204-5p

## Slide 19
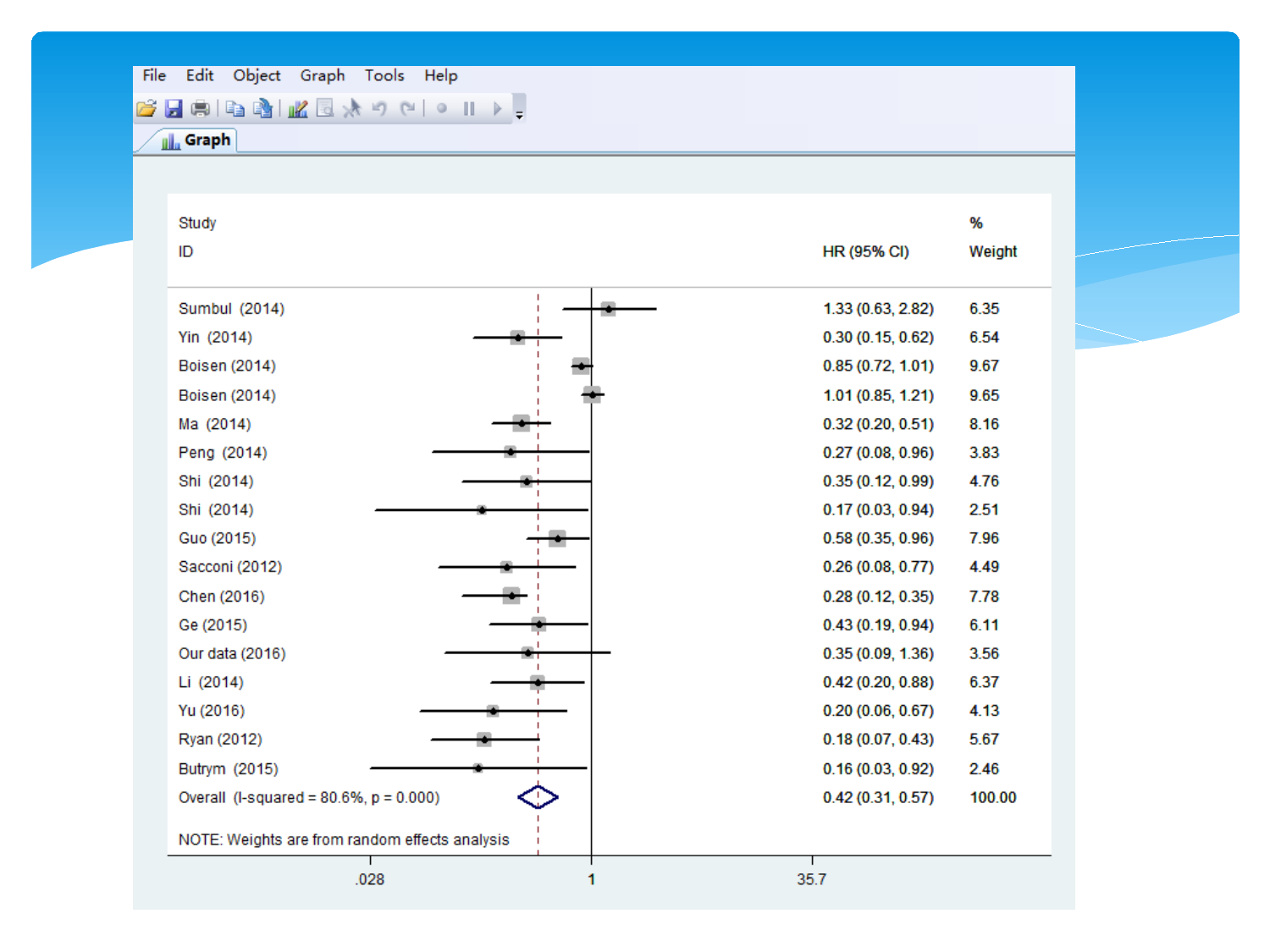

## Slide 20
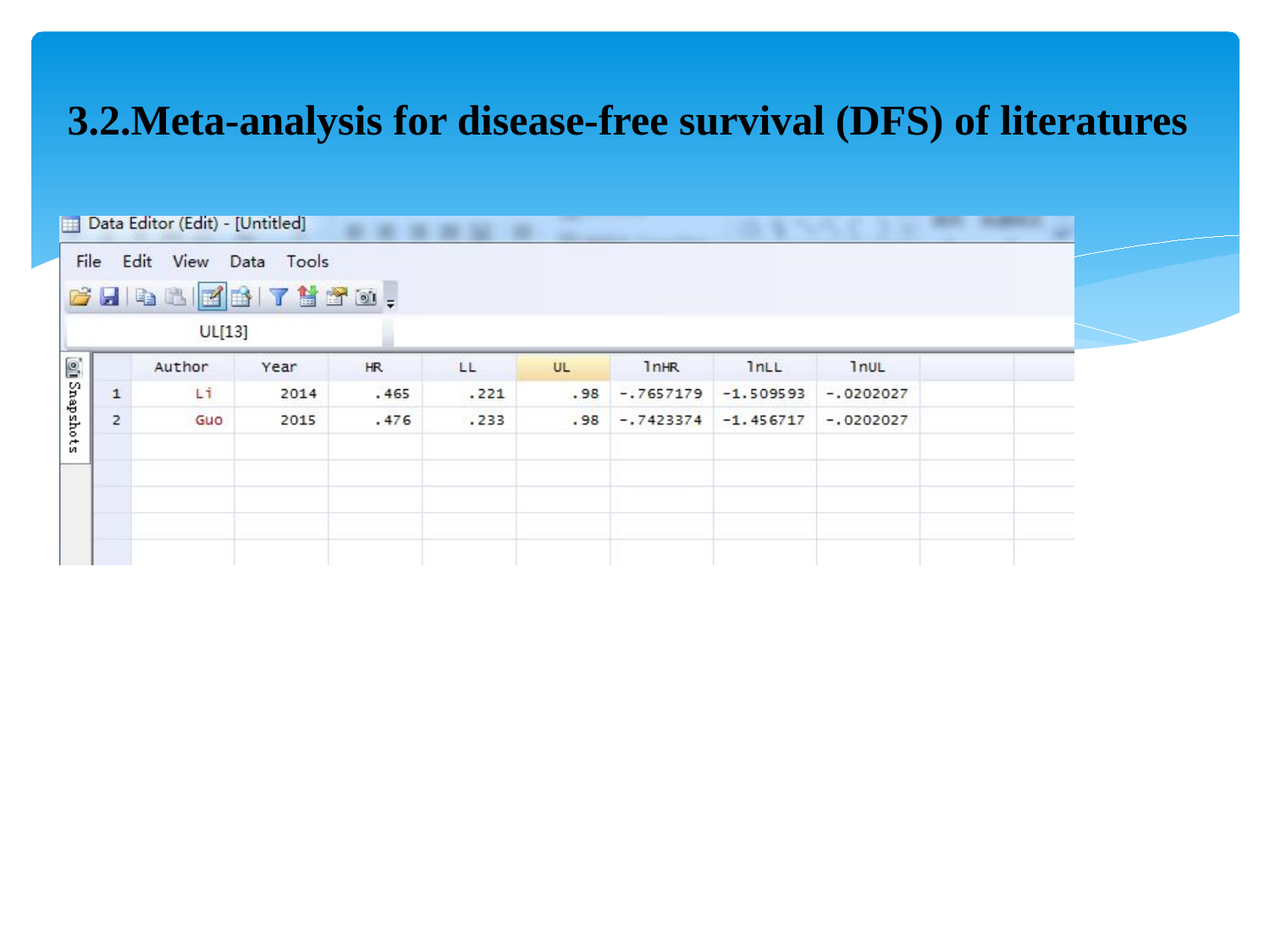

3.2.Meta-analysis for disease-free survival (DFS) of literatures

## Slide 21
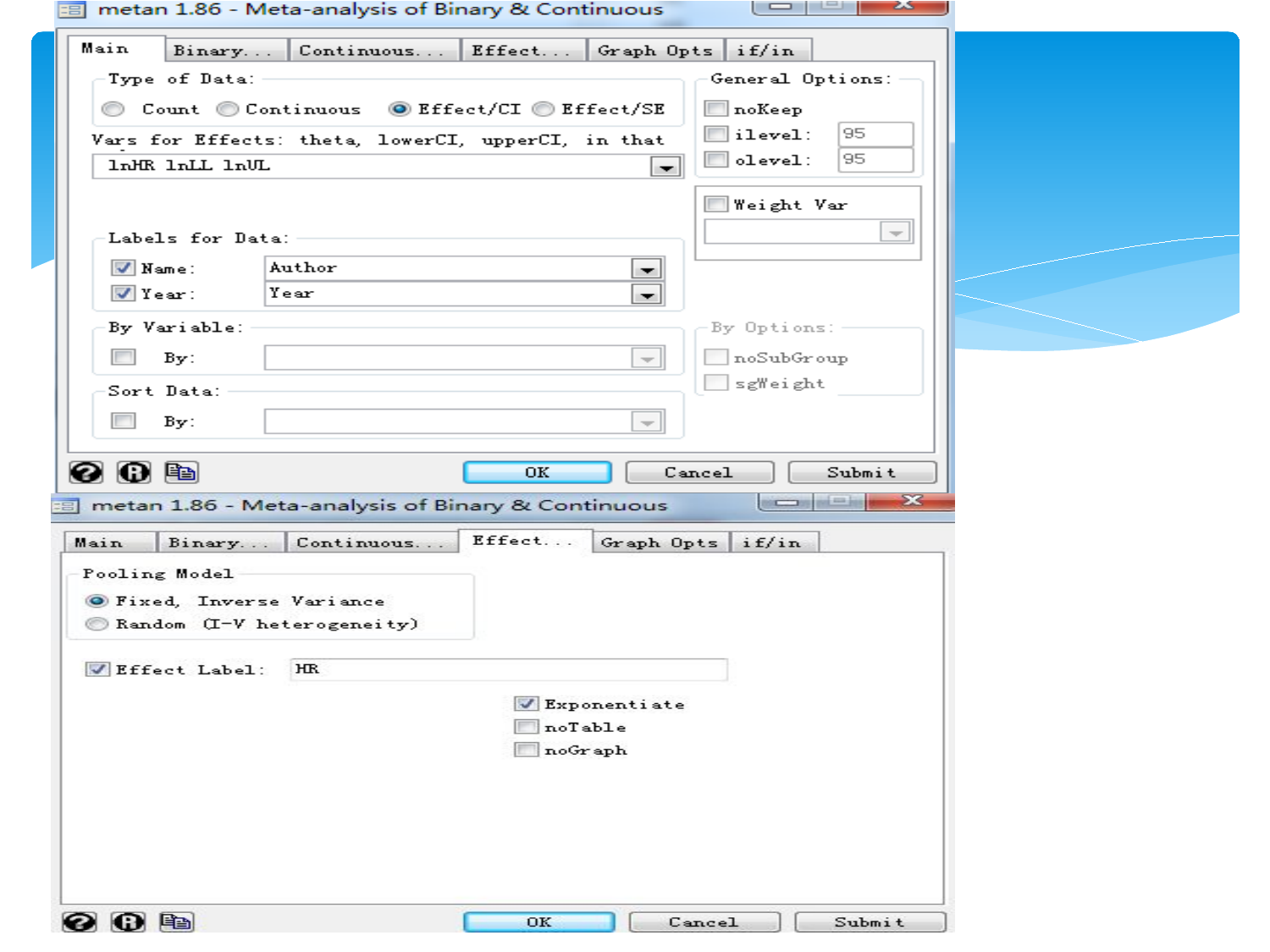

## Slide 22
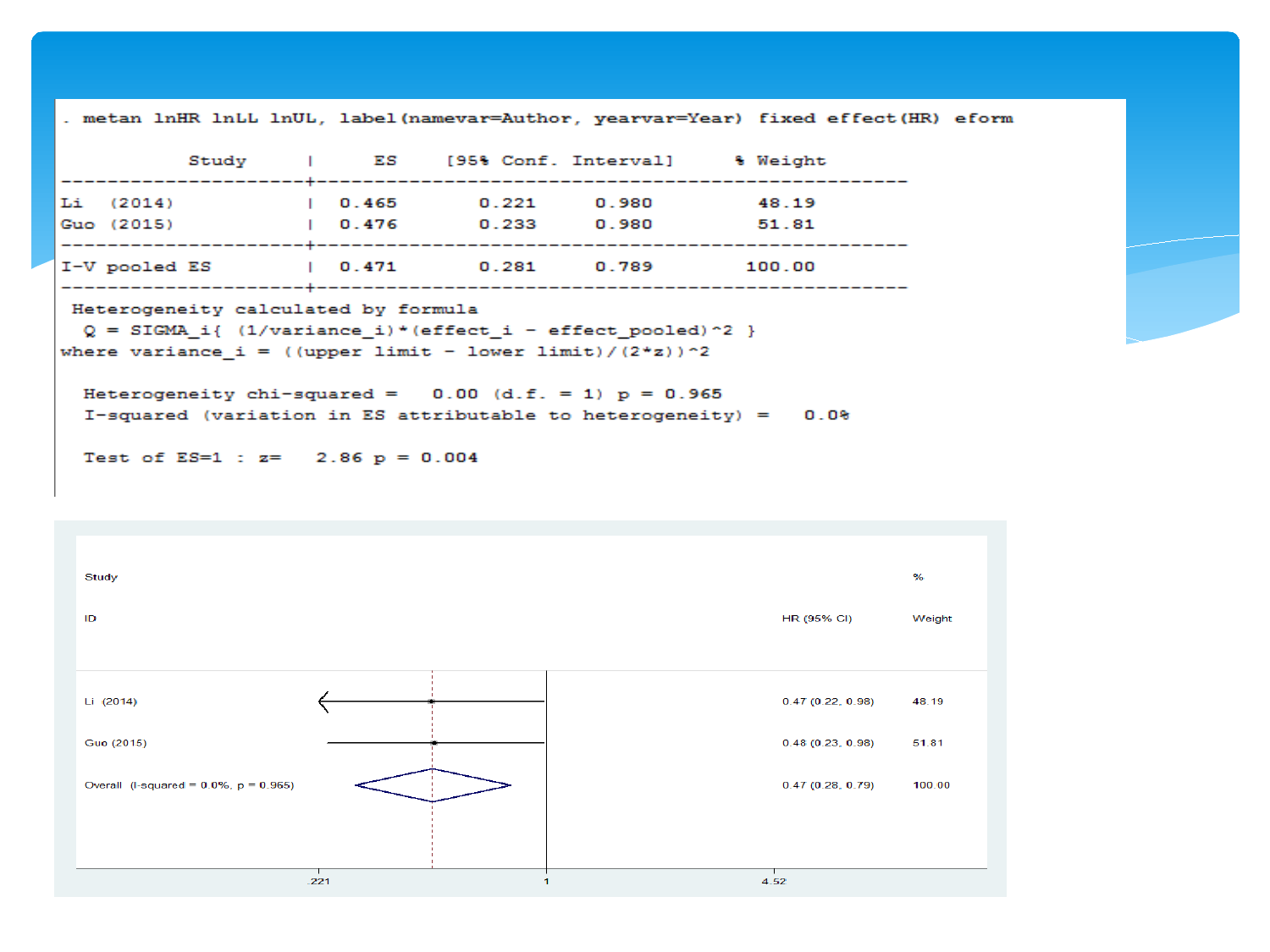

## Slide 23
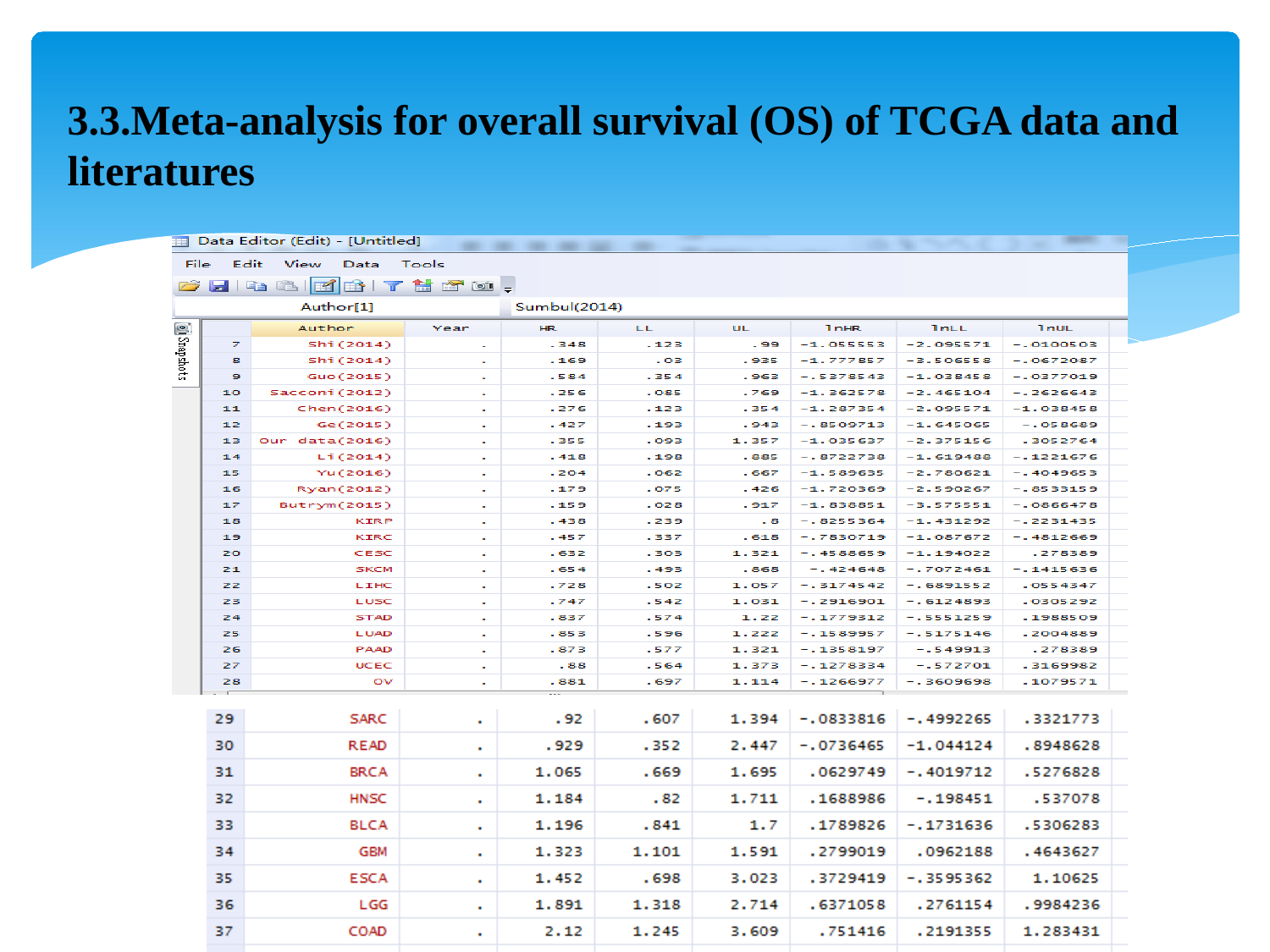

3.3.Meta-analysis for overall survival (OS) of TCGA data and literatures

## Slide 24
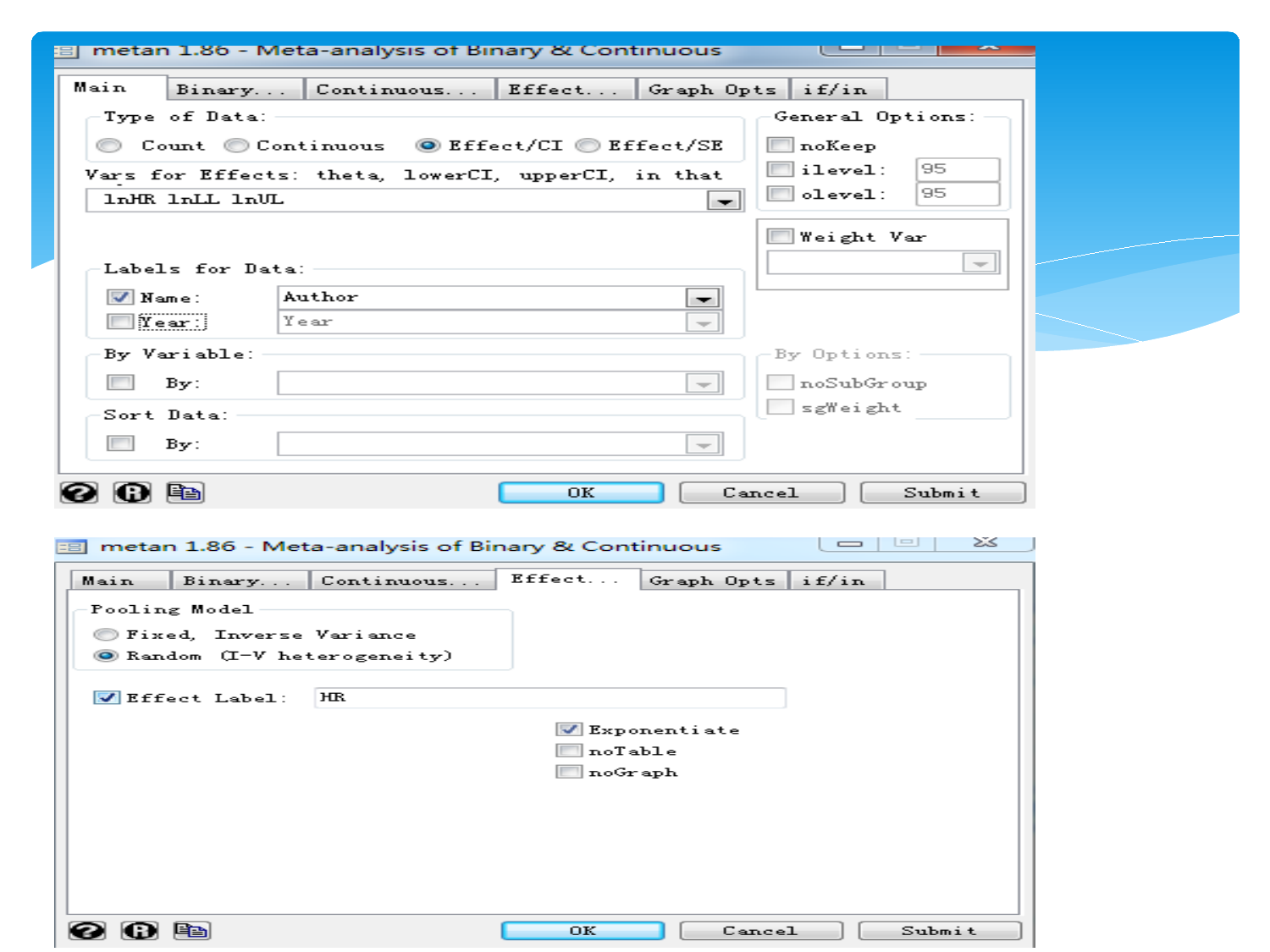

## Slide 25
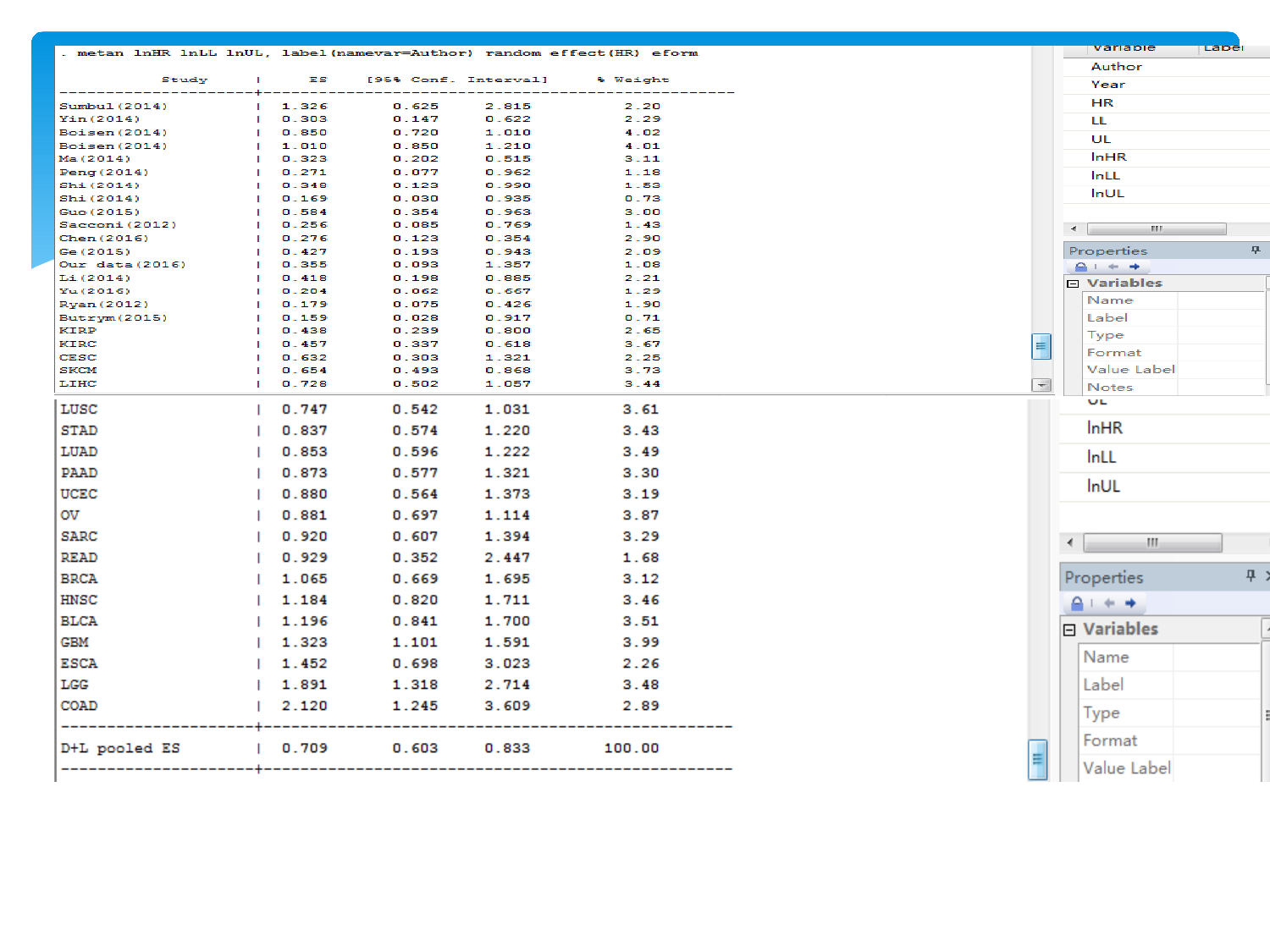

## Slide 26
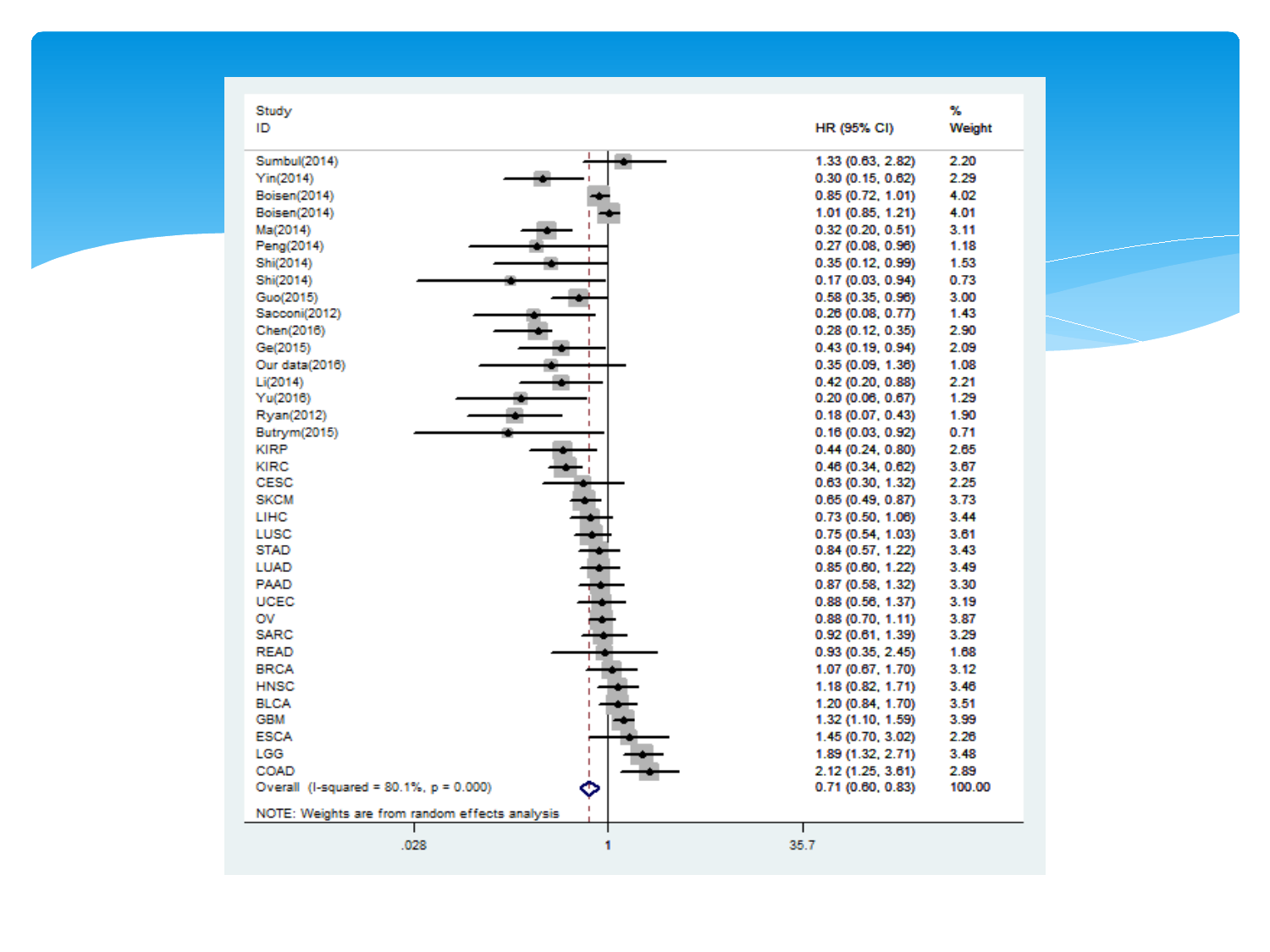

## Slide 27
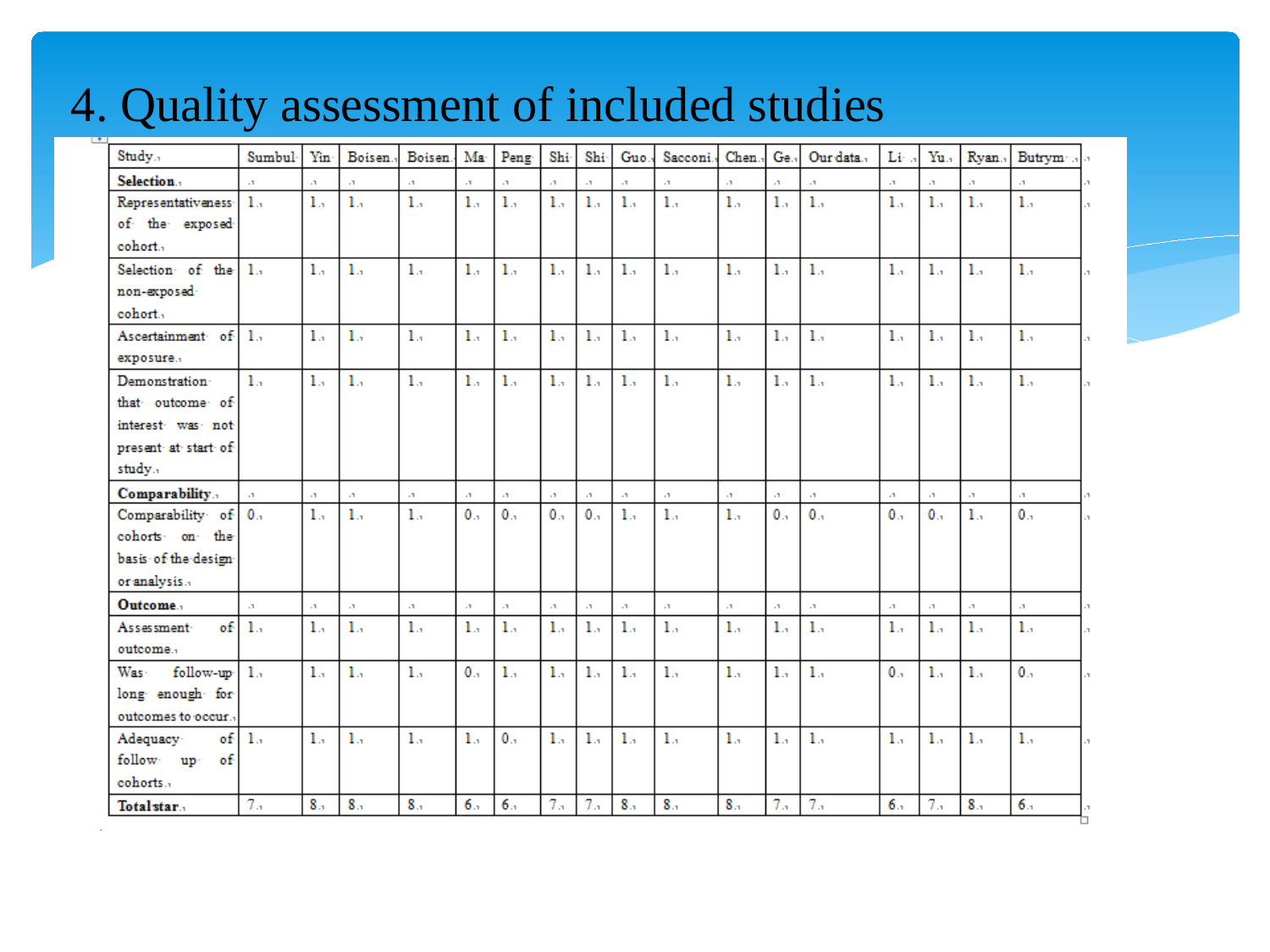

4. Quality assessment of included studies

## Slide 28
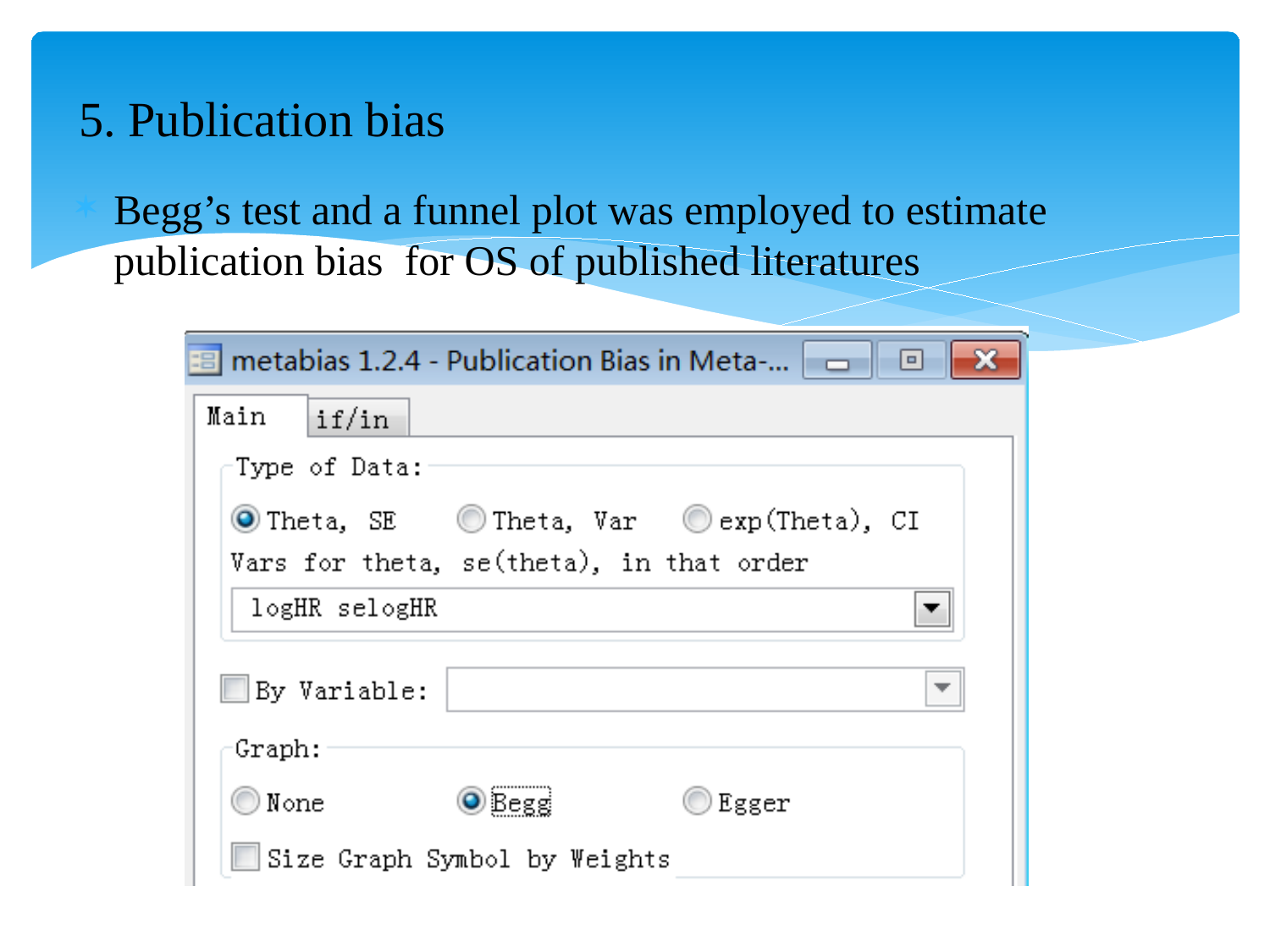

5. Publication bias
Begg’s test and a funnel plot was employed to estimate publication bias for OS of published literatures

## Slide 29
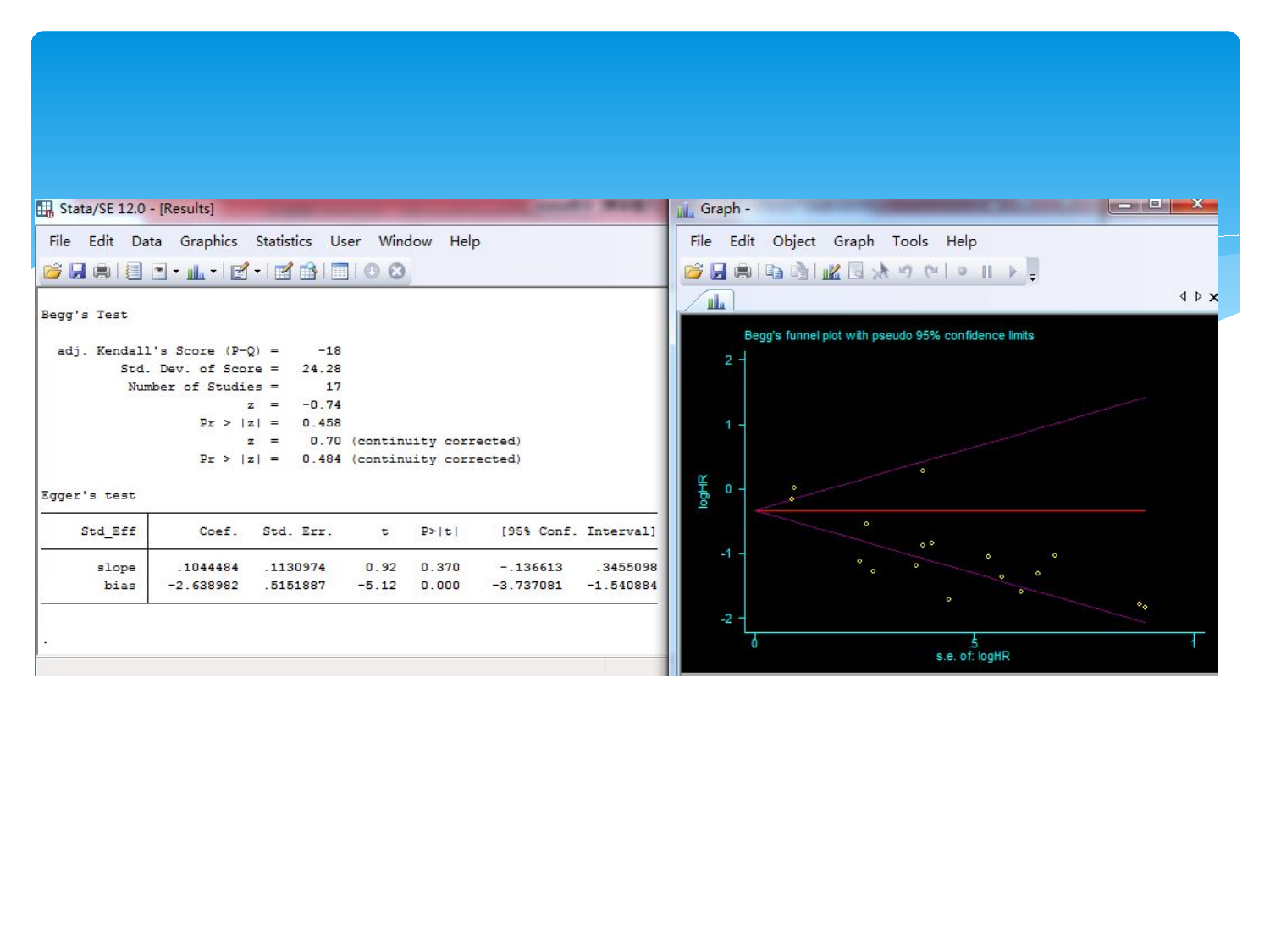

## Slide 30
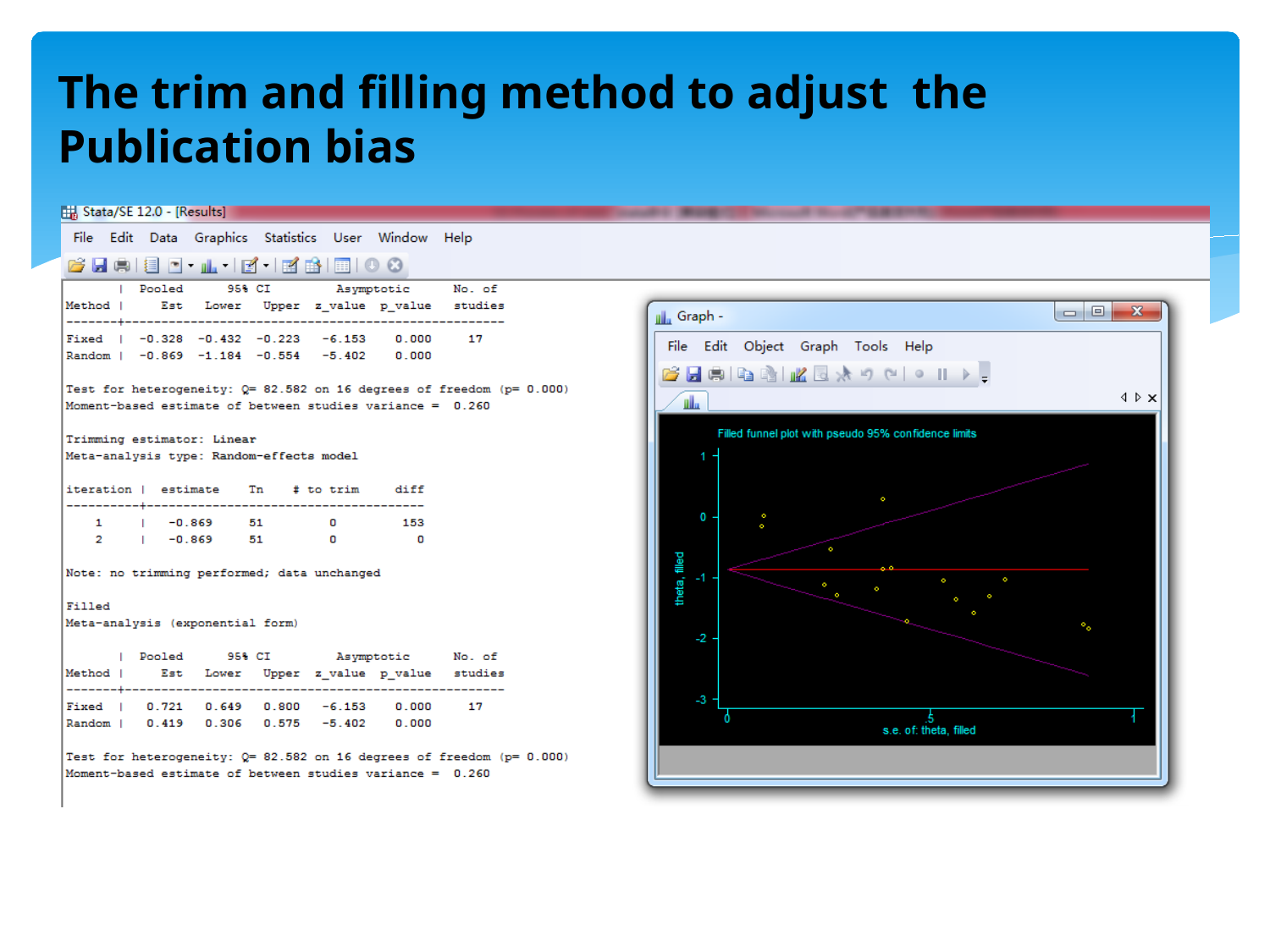

# The trim and filling method to adjust the Publication bias

## Slide 31
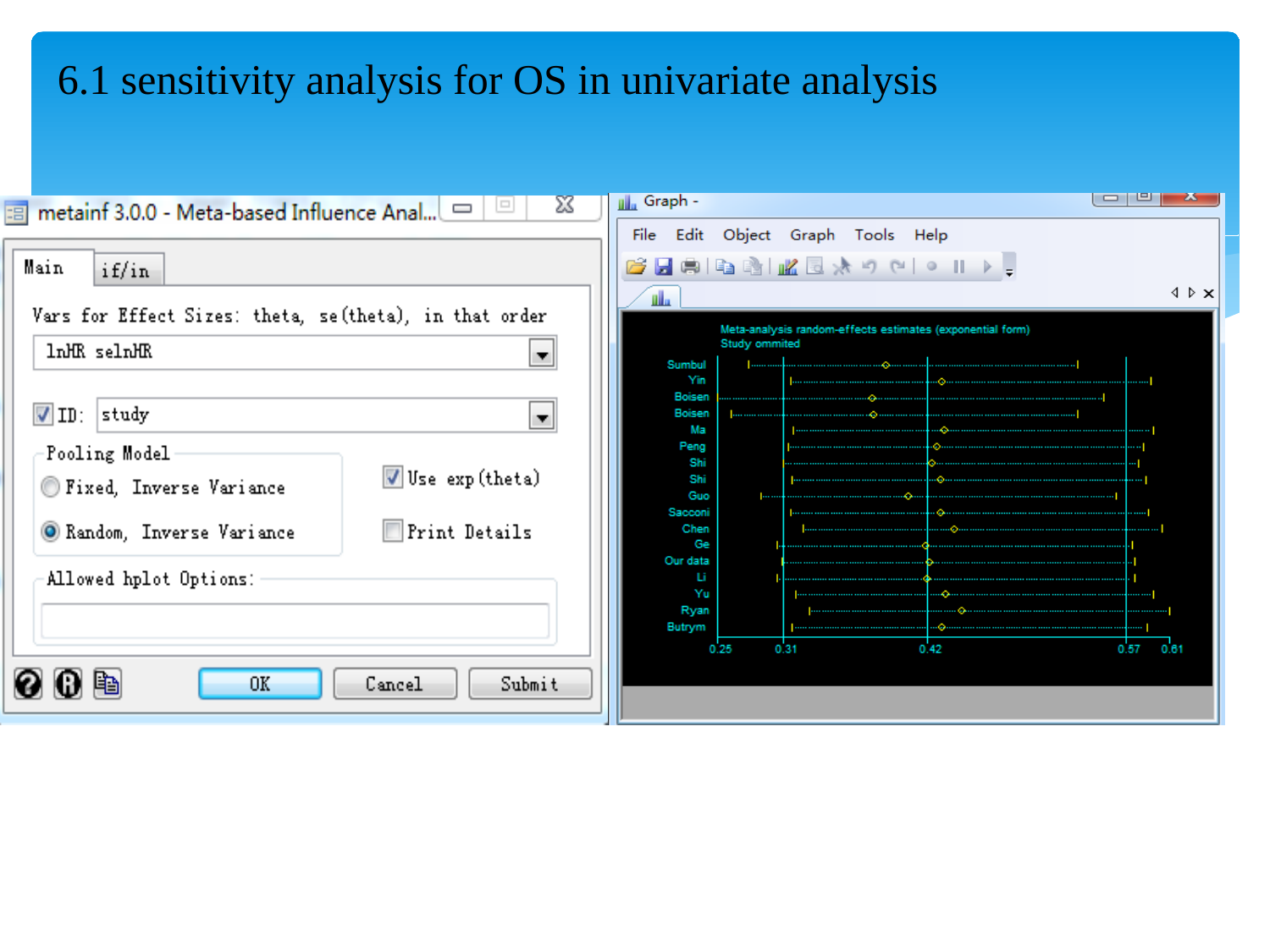

6.1 sensitivity analysis for OS in univariate analysis

## Slide 32
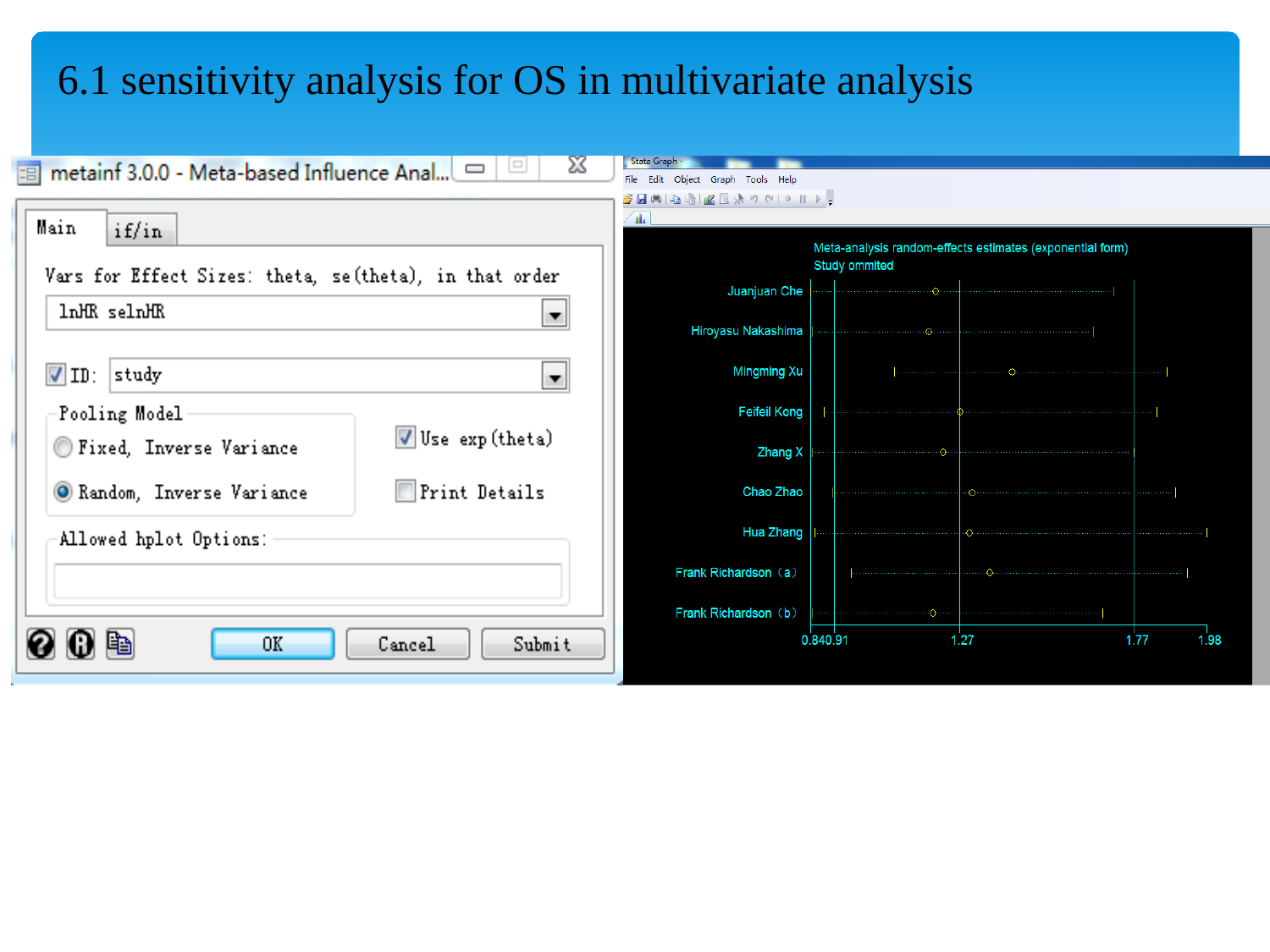

6.1 sensitivity analysis for OS in multivariate analysis

## Slide 33
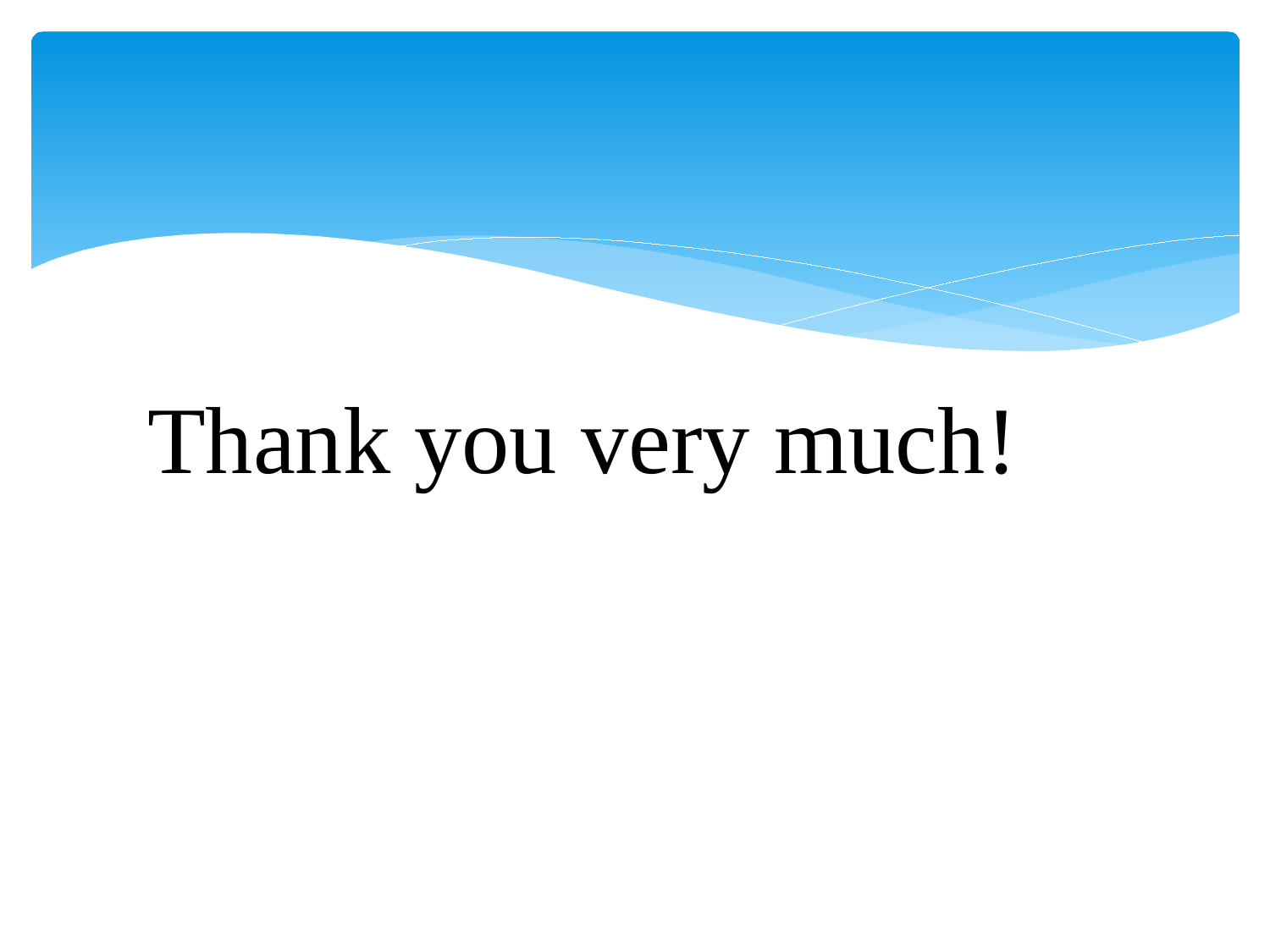

Thank you very much!
